# Stereotactic radiotherapy for neovascular age related macular degeneration: year 3 and 4 extended follow up results of a randomised, double masked, sham controlled, device trial (STAR)

**DOI:** 10.1136/bmj-2026-729694

**Published:** 2026-06-18

**Authors:** Timothy L Jackson, Riti Desai, Hatem A Wafa, Chan Ning Lee, Yanzhong Wang, Janet L Peacock, Tunde Peto, Usha Chakravarthy, Xuemin Zhu, Helen Dakin, Sarah Wordsworth, Patricia Clinch, Lisa Ramazzotto, James E Neffendorf, Joe M O’Sullivan, Barnaby C Reeves, on behalf of the STAR study group, Salwa Abugreen, Rashi Arora, Mandeep Bindra, Ben Burton, Indra Dias, Christiana Dinah, Ravikiran Gandhewar, Athanasios Georgas, Sheena George, Srinivas Goverdhan, Ansari Gulrez, Richard Haynes, Edward Hughes, Shahrnaz Izadi, Afsar Jafree, Sobha Joseph, Tarek Kashab, Silvana Mady, Luke Membrey, Geeta Menon, Aseema Misra, Niro Narendran, Douglas Newman, Jignesh Patel, Sudeshna Patra, Robert Petrarca, Priya Prakash, Ramiro Salom, Paritosh Shah, Marianne Shiew, Paul Tesha, Eleni Vrizidou

**Affiliations:** 1 Faculty of Life Sciences and Medicine, King’s College London, London, UK; 2 King’s Ophthalmology Research Unit (KORU), King’s College Hospital, London SE5 9RS, UK; 3 Department of Population Health Sciences, King’s College London, London, UK; 4 Department of Epidemiology, Geisel School of Medicine at Dartmouth, Dartmouth College, NH, USA; 5 Centre for Public Health, Queen’s University Belfast, Belfast, UK; 6 Network of Ophthalmic Reading Centres UK: London (Moorfields), Liverpool, and Belfast Ophthalmic Reading Centres, UK; 7 Nuffield Department of Population Health, University of Oxford, Oxford, UK; 8 Oxford NIHR Biomedical Research Centre, Oxford, UK; 9 Medical Engineering and Physics, King’s College Hospital, London, UK; 10 Bristol Medical School, University of Bristol, Bristol, UK

## Abstract

**Objective:**

To assess the effects of stereotactic radiotherapy (SRT) for neovascular age related macular degeneration (AMD) beyond the two year primary outcome of the StereoTactic radiotherapy for wet Age-Related macular degeneration (STAR) trial.

**Design:**

Randomised, double masked, sham controlled, device trial involving preplanned recall from standard care.

**Setting:**

30 NHS hospitals in the UK.

**Participants:**

411 participants aged at least 50 years with chronic, pretreated, active AMD.

**Intervention:**

Participants received one-off 16 Gray SRT or sham SRT delivered using a robotically controlled device. After two years of monthly study visits, participants reverted to routine care, with anti-VEGF drug selection and dosing intervals based on local practice, but with masking maintained, and repeat data collection at years 3 and 4 study visits.

**Main outcome measures:**

The main efficacy outcome at year 4 was the number of anti-VEGF injections, tested for superiority (fewer injections). The other main outcome was visual acuity, tested for non-inferiority (five letter margin). Safety outcomes included adverse events, serious adverse events, and microvascular abnormalities. The same analyses were undertaken at years 2, 3, and 4. A within trial costing analysis was undertaken for participants with four years’ follow-up.

**Results:**

Of 411 participants (204 (58%) women), 274 were allocated to SRT and 137 to sham SRT. The year 4 intention-to-treat efficacy analysis included 222 (81%) participants in the SRT group and 106 (77%) in the sham SRT group. The SRT group received a mean of 19.1 (standard deviation 10.9) injections over four years versus 21.6 (11.3) with sham SRT, an adjusted decrease of 3.2 injections (95% confidence interval (CI) of difference -5.7 to -0.7). During years 3 and 4, the SRT group received a mean cumulative 8.4 injections versus 8.3 injections in the sham SRT group. The final change in visual acuity in the SRT group was 8.3 letters worse than in the sham group (95% CI of difference -12.7 to -4.0). Adverse event rates were similar between groups, but reading centre-detected microvascular abnormalities occurred in 126/218 SRT treated eyes (58%) and 16/102 (16%) sham SRT treated eyes.

**Conclusion:**

Although the overall reduction in intravitreal therapy was maintained to year 4, the inferior vision in SRT treated eyes effectively reversed the conclusions of the year 2 primary outcome analysis and no longer supports the use of SRT to treat neovascular AMD. Including standard care, masked, extended follow-up within a clinical trial may provide useful clinical insight.

**Trial registration:**

ClinicalTrials.gov NCT02243878.

## Introduction

Neovascular age related macular degeneration (AMD) is a leading cause of blindness.^1^ Loss of vision occurs when abnormal new vessels grow into and beneath the neuroretina, usually from the choroidal circulation. These fragile vessels leak fluid and blood and untreated lead to secondary scarring with severe and permanent loss of central vision.

Neovascular AMD is treated with drugs directed against vascular endothelial growth factor (VEGF), a potent mediator of neovascularisation and vessel leakage. Anti-VEGF drugs are delivered by long term intravitreal injections, which are the most common eye procedure in many developed nations^2^ and carry financial burdens costing about $11bn (£8bn; €9bn) yearly in the US alone.^3^ Treatment regimens are burdensome, and assessments of patient priorities have identified the need for treatments that reduce the frequency of intravitreal injections for neovascular AMD.^4^ ^5^ Such injections require regular hospital attendance, can cause discomfort during and after the procedure, are associated with varying levels of patient anxiety, and have a small but cumulative risk of severe vision loss from intraocular infection.^4^ ^5^

As radiation mitigates many processes thought to underlie neovascular AMD, such as inflammation, neovascularisation, and fibrosis, it should have therapeutic potential.^6^ Consequently, researchers have attempted to use radiation as a treatment for neovascular AMD with varying success.^7^ ^8^ ^9^ Two randomised controlled trials investigated epimacular brachytherapy to treat neovascular AMD—a surgical technique that delivers radiation through a strontium source held over the macula. These trials failed to show efficacy,^8^ ^9^ with no reduction in the frequency of anti-VEGF injections.^8^

In contrast, two more recent randomised controlled trials investigating stereotactic radiotherapy (SRT), using a non-invasive, robotically controlled technique that delivers radiation via three overlapping radiation beams directed at the macula through the inferior sclera, met their primary endpoint of reducing the number of anti-VEGF injections required.^10^ ^11^ The IRay in conjunction with anti-VEGF treatment for patients with wet AMD (INTREPID) study recruited 230 participants and found a 29% and 37% reduction in anti-VEGF therapy at the year 1 primary endpoint, using 16 Gray (Gy) and 24 Gy SRT, respectively.^10^ An exploratory analysis found that a best responder subgroup had a 45% reduction in injection frequency at year 2.^12^ ^13^ Given that disease activity of neovascular AMD tends to fluctuate over weeks or sometimes a few months, a persisting benefit at year 2 suggests radiotherapy has a disease modifying effect.

The current STAR (StereoTactic radiotherapy for wet Age-Related macular degeneration) trial hypothesised that 16 Gy SRT reduces anti-VEGF injections while maintaining non-inferior vision versus sham SRT, and recruited 411 participants.^14^ The trial showed 2.9 (22%) fewer injections in the two years after SRT, and best corrected visual acuity that was non-inferior to sham SRT, at the prespecified five letter margin.^11^ The SRT group required progressively fewer anti-VEGF injections over time, relative to the sham SRT group, consistent with a disease modifying effect and suggesting longer follow-up may show even greater benefit.

After the two year primary endpoint, participants in STAR reverted to routine care, with local practice determining anti-VEGF drug selection and dosing intervals, but with full study evaluations at years 3 and 4 and masking maintained. The main aim was to assess the long term safety (particularly looking for delayed radiation retinopathy), and in so doing this afforded the opportunity to explore the durability of any therapeutic effect and to obtain more generalisable results for standard care. This report details STAR’s year 3 and 4 results.

## Methods

### Trial design

Details of the STAR trial^11^ and protocol^14^ have been published and are also included in the supplementary appendix. Briefly, STAR was a non-commercial, randomised, double masked, sham controlled device trial undertaken in 30 NHS hospitals in the UK. Participants provided written informed consent.

### Participants

Eligible participants were aged at least 50 years with chronic, pretreated, active neovascular AMD and at least three previous anti-VEGF injections, one within four months of randomisation, and a macular volume ≥8.15 mm^3^ (varying by machine) at the time of enrolment. We excluded people with diabetes, or study eyes with previous non-anti-VEGF therapy, foveal fibrosis, vision <24 letters according to the Early Treatment of Diabetic Retinopathy Study (ETDRS) vision chart, and lesions >4 mm or extending >2 mm from the foveal centre. Full eligibility criteria have been described^11^ ^14^ and are also provided in the supplementary appendix.

### Intervention, follow-up, and procedures

Briefly, randomisation was with variable block sizes ensuring 2:1 allocation of SRT and sham SRT, stratified by national treatment centre and centrally administered through an online randomisation system that generated an alphanumerical code to be entered into the SRT device.^11^ ^14^ The device then delivered either 16 Gy or sham dosing, following the same alignment checks, treatment cues, tracking, and gating algorithm protocols irrespective of allocation. Participants then returned to recruiting sites for study team review every four weeks up to 96 weeks (two years), receiving ranibizumab as required if prespecified retreatment criteria were met.^15^ Thereafter, participants returned to standard of care eye clinic review with anti-VEGF choice (aflibercept, bevacizumab, faricimab, or ranibizumab), retreatment criteria, dosing regimen, and follow-up intervals determined by local practice.

Except for the device technician who inputted the alphanumerical code into the device before trial commencement, and one statistician (HW), all participants, trial team, and treating investigators were masked to allocation. Masking was maintained throughout the four years of follow-up.

Participants returned to study teams for full study evaluations at weeks 144 (year 3) and 192 (year 4), comprising manifest refraction and best corrected visual acuity testing using the ETDRS chart and Age Related Eye Disease protocol^16^ with trial certified equipment and staff, slit-lamp examination, lens grading, spectral domain optical coherence tomography, digital fundus photography, fluorescein angiography, and five level EuroQol health related quality of life (EQ-5D-5L)^17^ and National Eye Institute 25 item visual function (NEI-VFQ-25)^18^ questionnaires. Angiography, fundus photography, and optical coherence tomography images were collected using trial certified equipment and trial staff, and transmitted to an independent reading centre (Central Administrative Research Facility, CARF, Belfast, Ireland) for masked analysis, using the same reading protocol as year 2.^11^ Data on the number and choice of intravitreal anti-VEGF drugs injected during years 3 and 4 were collected from injection logs, concomitant drug logs, and health service utilisation questionnaires.

### Outcome measures

As originally planned, the same efficacy and safety outcome measures reported at year 2 were repeated for years 3 and 4.^11^ The main efficacy evaluation comprised an exploratory analysis of the year 2 primary outcome measure (number of anti-VEGF injections, tested for superiority at years 3 and 4). Other exploratory secondary outcomes were described, including the percentage of participants losing <15 ETDRS letters, gaining ≥0 letters, gaining ≥15 letters, angiographic total lesion and choroidal neovascularisation size (mm^2^), foveal thickness (µm), EQ-5D-5L utility responses, and NEI-VFQ-25 composite score. Key safety outcome measures were adverse events and serious adverse events, with particular focus on investigator and reading centre detected microvascular abnormalities and radiation retinopathy. ETDRS best corrected visual acuity was both a key safety and a secondary efficacy outcome, tested for non-inferiority at a five letter margin.

### Adherence and covid-19 pandemic

Details of the STAR trial sensitivity analyses and depiction of trial adherence using a novel “compliance cube” have been reported.^11^ This cube was updated to include visits at weeks 144 and 192. Sensitivity analyses undertaken with the weeks 48 and 96 data were repeated for weeks 144 and 192 for several overlapping populations: participants completing specific timepoints both before and after lockdown in the UK during the covid-19 pandemic, and participants whose adherence was sufficient (adherent group) and compromised (reduced adherence group). Supplementary appendix table S7 shows the distinction between adherent and reduced adherence groups, with the criteria agreed by the majority independent trial steering committee, and as specified in the protocol and statistical analysis plan before data lock (see supplementary appendix).

### Statistical analysis

The predefined efficacy analysis at year 2 has been described elsewhere.^11^ Briefly, the primary outcome was the mean number of ranibizumab injections over 96 weeks (two years) tested for superiority, and the main secondary outcome was change in ETDRS best corrected visual acuity from baseline to week 96, tested for non-inferiority at a five letter margin.

Multiple linear regression was used to estimate treatment effects for these outcomes, including national treatment centre as a covariate, and other secondary outcomes were summarised descriptively as mean (standard deviation, SD), median (interquartile range, IQR), or count (percentage). The same prespecified analysis plan was applied to the extended follow-up data with a primary focus on long term safety. Ongoing prospective data collection of all the year 2 efficacy measures allowed exploratory analysis of efficacy in a standard clinical setting at years 3 and 4. Analyses followed the intention-to-treat principle, with safety assessed per protocol on available data. To address missing data, multiple imputation with chained equations was applied to generate 20 datasets, using baseline characteristics, ophthalmic history, and recruitment site to impute the incomplete values in the outcome variable, with the resulting estimates combined using Rubin’s principles. Sensitivity analyses assessed the findings’ robustness, including to non-adherence.

#### Subgroup and post hoc analyses

Predefined subgroup analyses undertaken at year 2 were repeated for years 3 and 4, with the addition of a post hoc subgroup encompassing participants who switched anti-VEGF agents (from ranibizumab to another agent) after year 2. The year 2 analysis included a post hoc analysis evaluating the effect of microvascular abnormalities on visual acuity,^11^ which was repeated for years 3 and 4. A post hoc analysis explored whether incongruous best corrected visual acuity and NEI-VFQ-25 scores might be explained by whether the study eye was the better or worse seeing eye. Another post hoc analysis looked for variables that might explain reduced best corrected visual acuity in the SRT group (supplementary appendix table S1).

### Costing analysis

A costing analysis was undertaken for participants with follow-up for four years, using the same methods as previously described (see supplementary appendix for details).^11^ Briefly, we compared neovascular AMD treatment costs for SRT plus ranibizumab as needed versus ranibizumab monotherapy as needed. After two years, because participants received differing follow-up intervals, regimens, and anti-VEGF drugs, a validation process estimated resource utilisation and monitoring costs (supplementary appendix). The costing analysis included the cost of SRT, monitoring consultations, and anti-VEGF injections in patients with 192 weeks’ follow-up.

### Patient and public involvement

The Macular Society provided feedback on the bid for funding, project plan, protocol, and patient facing documents, including feedback from groups of patients with AMD. A Macular Society member represented patients’ interests on the trial steering committee.

## Results

**Fig 1:**
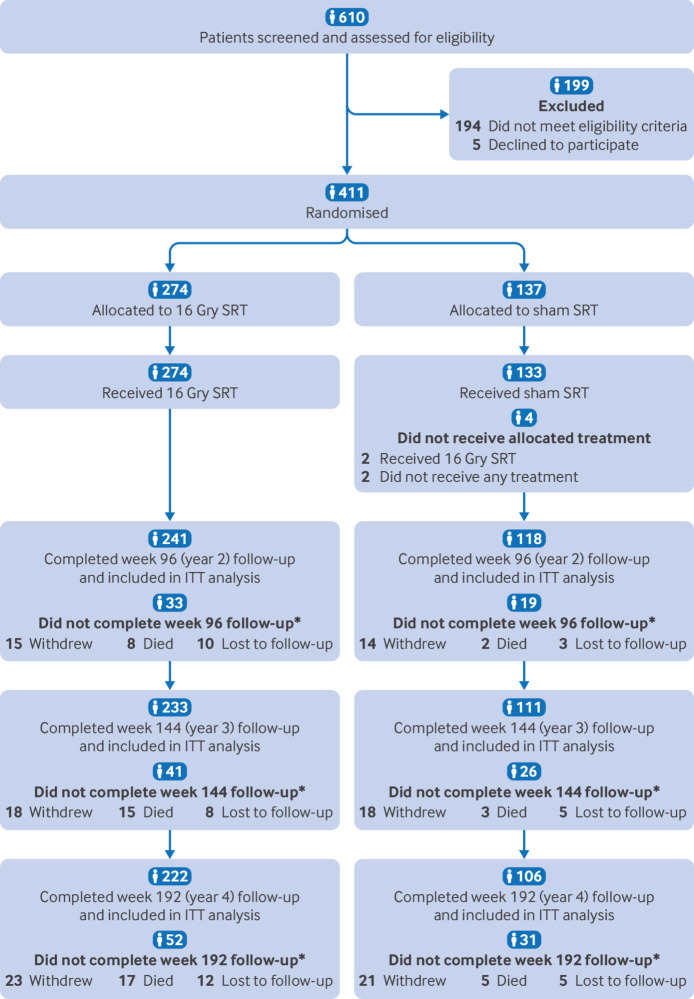
CONSORT diagram. *Numbers are cumulative. Gry=Gray; ITT=intention to treat; SRT=stereotactic radiotherapy

Of the 411 randomised participants recruited between 1 January 2015 and 27 December 2019, 359 (87%), 344 (84%), and 328 (80%) completed follow-up at years 2, 3, and 4, respectively (fig 1). Supplementary appendix tables S5 and S6 provide details on missingness and the reasons for missingness in participants. Table 1 shows the baseline characteristics for all participants. Of participants completing years 3 and 4 follow-up, those lost to follow-up were slightly more likely to have an artificial lens and to report mobility issues on EQ-5D-5L indices but were otherwise similar (supplementary appendix table S28).

**Table 1:** Baseline characteristics of participants by allocated treatment. Values are number (percentage) unless stated otherwise

Characteristics	SRT group (n=274)	Sham SRT group (n=137)
**Personal characteristics**		
Mean (SD) age (years)	78 (7.0)	78 (7.4)
Women	158 (58)	82 (60)
Ethnicity:		
White	268 (98)	129 (94)
Black or black British	1 (<1)	0
Asian or Asian British	3 (1)	7 (5)
Other	2 (1)	1 (<1)
Smoking status:		
Current smoker	27 (10)	15 (11)
Former smoker	114 (42)	61 (44)
Non-smoker	133 (49)	61 (45)
**Ophthalmic characteristics**		
Angiographic lesion subtype:		
Classic	24 (9)	4 (3)
Minimally classic	20 (7)	10 (7)
Occult	181 (66)	96 (70)
RAP	13 (5)	6 (4)
IPCV	21 (8)	14 (10)
Ungradable	15 (6)	7 (5)
Median (IQR) duration of neovascular AMD (months)	22 (11-45)	22 (12-42)
Median (IQR) No of previous anti-VEGF injections	7 (5-9)	7 (5-10)
ETDRS visual acuity (letter score)	68.4 (12.9)	69.1 (13.7)
Lens status:		
Aphakic	1 (<1)	2 (1)
Pseudophakic	92 (34)	45 (33)
Phakic	181 (66)	90 (66)
Central subfield thickness (μm)	349 (115)	343 (130)
Median (IQR) total lesion size (mm^2^)	6.9 (3.8-11)	7.1 (4.1-11)
Median (IQR) total active lesion size (mm^2^)	6.5 (3.7-10)	6 (4-10)
Mean (SD) total macular volume (mm^3^)	8.8 (1.2)	8.9 (1.3)
Patient reported outcome measures		
Median (IQR) NEI VFQ-25 composite score	87 (77-94)	87 (70-93)
Median (IQR) EQ-5D-5L (VAS)	90 (80-95)	85 (75-95)
**EQ-5D-5L dimension**		
Mobility:		
No problems	181 (68)	83 (61)
Slight problems	37 (14)	30 (22)
Moderate problems	37 (14)	19 (14)
Severe problems	10 (4)	4 (3)
Extreme problems	2 (<1)	0
Self-care:		
No problems	247 (93)	126 (93)
Slight problems	12 (4)	8 (6)
Moderate problems	5 (2)	2 (1)
Severe problems	2 (<1)	0
Extreme problems	1 (<1)	0
Usual activities:		
No problems	191 (72)	94 (69)
Slight problems	48 (18)	23 (17)
Moderate problems	22 (8)	17 (12)
Severe problems	5 (2)	1 (<1)
Extreme problems	1 (<1)	1 (<1)
Pain/discomfort:		
No problems	144 (54)	76 (56)
Slight problems	74 (28)	31 (23)
Moderate problems	38 (14)	21 (15)
Severe problems	9 (3)	8 (6)
Extreme problems	2 (<1)	0
Anxiety/depression:		
No problems	221 (83)	109 (80)
Slight problems	30 (11)	21 (15)
Moderate problems	15 (6)	6 (4)
Severe problems	1 (<1)	0
Extreme problems	0	0

All ophthalmic history variables relate to the study eye. Supplementary tables S5 and S6 provide details on missingness.

AMD=age related macular degeneration; anti-VEGF=anti-vascular endothelial growth factor; EQ-5D-5L (VAS)=EuroQol-5D questionnaire with visual analogue scale; ETDRS=Early Treatment Diabetic Retinopathy Study; IQR=interquartile range; IPCV=idiopathic polypoidal choroidal vasculopathy; NEI-VFQ-25=National Eye Institute (USA) 25 item visual function questionnaire; RAP=retinal angiomatous proliferation; SD=standard deviation; SRT=stereotactic radiotherapy.

**Fig 2:**
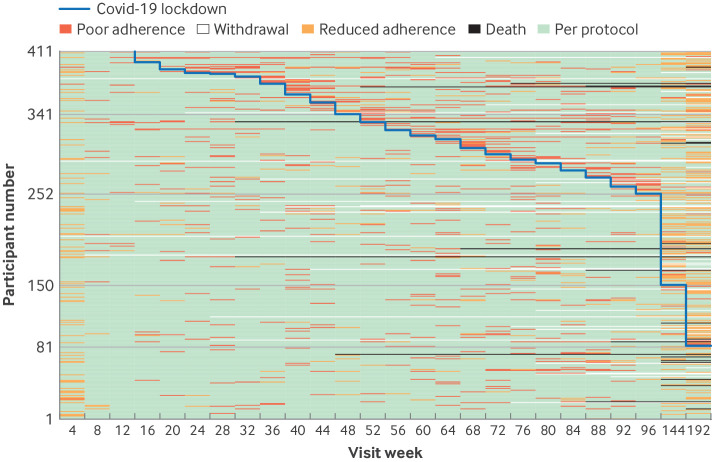
“Compliance cube” showing participants’ overall adherence to the trial protocol. Each participant’s sequential visits are shown in a single row from left to right, with green showing an adherent visit, amber showing reduced adherence for that visit, but not one likely to affect the main outcome, and red showing reduced adherence that might affect the main outcome (see supplementary table S7 for definitions). Participant’s timelines are then stacked bottom to top, from the first to last participant recruited. Hence each participant’s adherence over time can be viewed from left to right, and overall adherence to the trial can be viewed from bottom to top. The blue line shows the onset of the covid-19 lockdown in the UK (onset 23 March 2020), to enable comparisons before and after lockdown

Figure 2 is a novel means of depicting adherence.^11^ The compliance cube illustrates participants’ deviations during the trial, classifying each visit as adherent (green), a deviation not likely to affect the main outcome (amber), and a deviation that might affect the main outcome (red). The allocation to these categories utilised predefined rules agreed by the majority independent trial steering committee, that were finalised before data lock (supplementary appendix table S7). The diagram shows each participant’s consecutive visits (coded green, amber, or red) arranged from left to right in a single row. As further participants enrol, they are stacked on top of these rows, from first to last recruited. Thus, the diagram shows each participant’s adherence over time (left to right) and overall adherence to the trial over time (bottom to top). More deviations occurred in participants who had at least one visit after the covid-19 lockdown (80%, 330/411). Most, however, were amber visits, representing a deviation that was unlikely to affect the main outcome.

**Fig 3:**
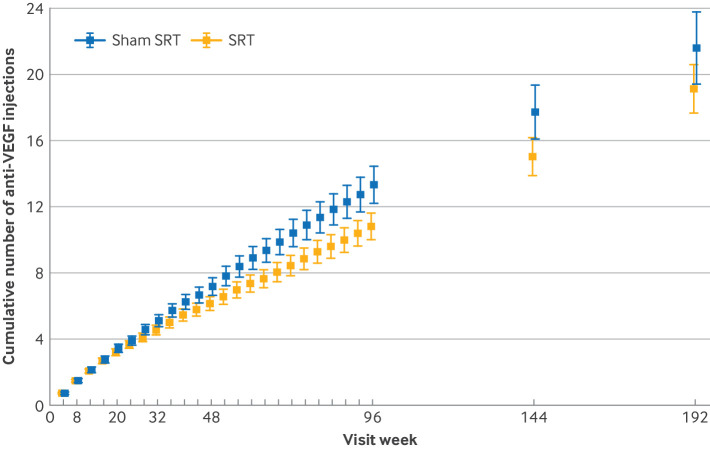
Mean cumulative number of anti-VEGF injections in the SRT and sham SRT groups by visit week. Error bars show the 95% confidence intervals. Anti-VEGF=anti-vascular endothelial growth factor; SRT=stereotactic radiotherapy

Table 2 and supplementary table S8 show the main and other efficacy outcomes. At year 2, the SRT group received a cumulative mean of 15.0 (SD 8.8) anti-VEGF injections versus 17.7 (8.6) in the sham SRT group; 3.1 fewer injections (95% confidence interval (CI) of difference -5 to -1.2) after adjusting for national treatment centre (table 2). This difference was maintained at year 4 (fig 3), with the SRT group receiving a cumulative mean of 19.1 (10.9) injections versus 21.6 (11.3) in the sham SRT group, an adjusted 3.2 fewer injections (95% CI of difference -5.7 to -0.7). Supplementary figures S1 and S2 provide a breakdown of injection frequency, and supplementary table S14 shows cumulative injections by month. In year 3 (weeks 96 to 144) and year 4 (weeks 144 to 192), participants in the SRT group received a mean cumulative 4.3 and 4.1 injections each year, respectively, versus 4.4 and 3.9 injections in the sham SRT group. Across both years (weeks 96 to 192), participants in the SRT group received a mean cumulative 8.4 injections compared with 8.3 injections in the sham SRT group.

**Table 2:** Main and other efficacy outcomes at weeks 48, 96, 144, and 192. Values are number (percentage) unless stated otherwise

	SRT group	Sham SRT group	Adjusted regression coefficient of difference (95% CI), P value
**Mean (SD) No of anti-VEGF injections**			
Week 48	6.1 (3.4)	7.1 (3·0)	-1.1 (-1.8 to -0.4), 0.001
Week 96	10.7 (6.3)	13.3 (5.8)	-2.9 (-4.2 to -1.6), <0.001
Week 144	15.0 (8.8)	17.7 (8.6)	-3.1 (-5 to -1.2), 0.002
Week 192	19.1 (10.9)	21.6 (11.3)	-3.2 (-5.7 to -0.7), 0.011
**Mean (SD) change in ETDRS visual acuity**			
Week 48	0.0 (8.8)	0.0 (8.8)	-0.7 (-2.6 to 1.1), 0.44
Week 96	-2.9 (11.1)	-1.5 (11.2)	-1.7 (-4.2 to 0.8), 0.17
Week 144	-9.8 (16.0)	-3.4 (14.5)	-6.5 (-10.2 to -2.8), <0.001
Week 192	-15.8 (19.5)	-7.0 (15.7)	-8.3 (-12.7 to -4.0), <0.001
**Mean (SD) ETDRS visual acuity**			
Week 48	68.2 (14.4)	69.8 (14.5)	-
Week 96	65.4 (15.0)	68.5 (15.6)	-
Week 144	58.8 (18.6)	65.8 (18.3)	-
Week 192	53.1 (21.1)	62.8 (19.7)	-
**Losing <15 ETDRS letters**			
Week 48	245 (95)	120 (95)	-
Week 96	209 (87)	112 (93)	-
Week 144	160 (70)	95 (89)	-
Week 192	132 (59)	80 (78)	-
**Gaining ≥0 ETDRS letters**			
Week 48	140 (54)	69 (55)	-
Week 96	107 (45)	58 (48)	-
Week 144	73 (32)	49 (46)	-
Week 192	44 (20)	34 (33)	-
**Gaining ≥15 ETDRS letters**			
Week 48	9 (4)	3 (2)	-
Week 96	7 (3)	3 (3)	-
Week 144	7 (3)	4 (4)	-
Week 192	2 (<1)	4 (4)	-
**Median (IQR) total lesion size (mm^2^)**			
Week 48	7.2 (4.4-12)	7.1 (4.2-12)	-
Week 96	8.2 (4.5-12)	7.3 (4.1-11)	-
Week 144	8.1 (5·1-12)	8.6 (4.9-14)	-
Week 192	7·7 (4·3-12)	8.5 (4.6-14)	-
**Median (IQR) total active lesion size (mm^2^)**			
Week 48	6.9 (4.2-11)	6.6 (3.9-11)	-
Week 96	7.1 (4.4-12)	6.4 (3.6-11)	-
Week 144	8.0 (4.6-13)	6.5 (3.6-12)	-
Week 192	5.6 (3.6-11)	6.8 (3.3-11)	-
**Mean (SD) central subfield thickness (μm)**			
Week 48	296.6 (112.1)	320.6 (119.5)	-
Week 96	305.1 (130.5)	307.3 (100.6)	-
Week 144	289.0 (125.6)	327.8 (245.6)	-
Week 192	303.4 (195.5)	277.4 (94.8)	-
**Median (IQR) NEI VFQ-25 composite score**			
Week 48	88 (76-95)	87 (73-94)	-
Week 96	88 (73-94)	86 (66-94)	-
Week 144	87 (67-93)	83 (62-94)	-
Week 192	82 (66-92)	81 (65-92)	-
**Median (IQR) EQ-5D-5L (VAS)**			
Week 48	85 (75-95)	82 (75-92)	-
Week 96	85 (75-95)	80 (70-90)	-
Week 144	85 (75-90)	80 (70-90)	-
Week 192	80 (70-90)	80 (70-90)	-

Values are unadjusted, except for the regression coefficients, which are adjusted for the national treatment centre in the case of the main outcome, and for both the national treatment centre and the baseline visual acuity in the case of the change in visual acuity score. Structural outcomes (lesion size, active lesion size, and central subfield thickness) are those from the independent reading centre.

CI=confidence interval; EQ-5D-5L (VAS)=EuroQol questionnaire with visual analogue scale and five level vision bolt-on; ETDRS=Early Treatment Diabetic Retinopathy Study; IQR=interquartile range; NEI VFQ-25=National Eye Institute (USA) 25 item visual function questionnaire; SD=standard deviation; SRT=stereotactic radiotherapy.

Sensitivity analyses supported the main finding (supplementary tables S9-S12), with similar results in those who completed year 4 before the covid-19 lockdown (2.9 fewer injections in the SRT group, 95% CI -8.3 to 2.5) and those who were fully adherent (2.9 fewer injections, -5.6 to -0.1).

**Fig 4:**
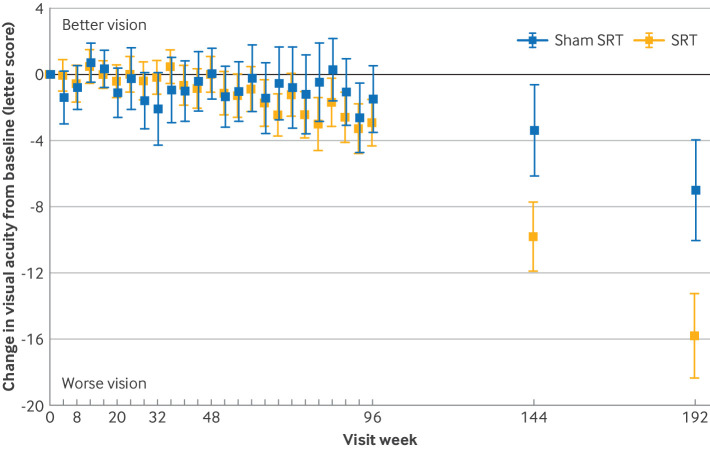
Mean change in Early Treatment Diabetic Retinopathy Study best corrected visual acuity in the SRT and sham SRT groups by visit week. Errors bars show the 95% confidence intervals. SRT=stereotactic radiotherapy

The SRT group had a greater decrease in mean change in ETDRS best corrected visual acuity compared with the sham SRT group, with a difference of -6.5 letters (95% CI -10.2 to -2.8) at year 3 and -8.3 letters (-12.7 to -4.0) at year 4, after adjusting for national treatment centre (table 2, fig 4). This was despite smaller median lesion area (7.7 *v* 8.5 mm^2^) and smaller active lesion area (5.6 *v* 6.8 mm^2^) in the SRT group at year 4 (table 2).

Despite worse vision in the SRT group, the median NEI-VFQ-25 composite scores (82 (IQR 66-92) *v* 81 (65-92)) and EQ-5D-5L visual analogue scale (80 (70-90) both groups) responses were similar between groups at year 4 (table 2, and supplementary table S8). Post hoc analysis (supplementary table S16) showed that 63% (260/411) of study eyes were the worse seeing eye at baseline. At year 4, median NEI-VFQ-25 composite scores in the SRT group were higher among participants whose study eye was the worse seeing eye at baseline than among those whose study eye was the better seeing eye (87 (IQR 72-94) *v* 70 (50-84)). The finding was similar to the sham SRT group (88 (75-94) *v* 75 (52-84)). A gradual decline also occurred in median NEI-VFQ-25 composite score from year 1 onwards in all participants whose study eye was the better seeing eye, whereas participants whose study eye was the worse seeing eye retained steady composite scores throughout the trial (supplementary table S16).

Supplementary tables S13 and S29 respectively show secondary outcomes and baseline characteristics disaggregated by sex. Men treated with SRT had a greater benefit than women treated with SRT at year 4, with 5.6 versus 1.9 fewer injections than with sham SRT (95% CI of difference -9.5 to -1.7 and -5.3 to 1.4, respectively). At year 4, men in the SRT group lost 5.0 more letters than men in the sham SRT group (95% CI of difference -11.7 to 1.6), whereas women in the SRT group lost 11.2 more letters than women in the sham SRT group (-17.2 to -5.1).

**Fig 5:**
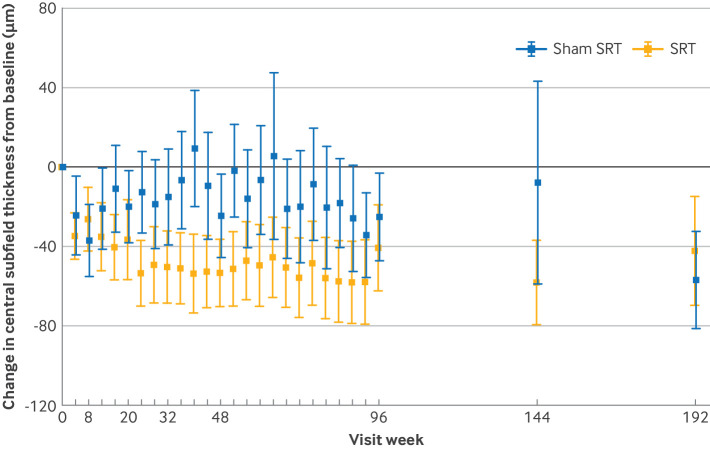
Mean change in the macular central subfield thickness measurement from baseline to week 144 (year 3) and week 192 (year 4). The automated measurements were taken from each site’s spectral domain optical coherence tomography device, but with manual correction of any segmentation errors by site investigators, if required. Errors bars show the 95% confidence intervals. SRT=stereotactic radiotherapy

At year 4, the SRT group tended to have slightly greater (38 µm) neurosensory thickness, driven mainly by greater (34 µm) subretinal fluid volume (fig 5, and supplementary table S15). Conversely, the pigment epithelial detachment height in the SRT group tended to be lower (-19 µm) than in the sham SRT group.

Supplementary figures S5-S10 present the prespecified subgroup analyses, with the post hoc analyses of participants who switched anti-VEGF from ranibizumab. All analyses of anti-VEGF frequency favoured SRT over sham SRT at years 3 and 4, except for a small number of participants with classic, retinal angiomatous proliferation, mixed, or ungradable neovascular AMD. In all subgroups, best corrected visual acuity favoured sham SRT over SRT, except for a few participants with ungradable lesions at year 4.

Supplementary tables S17-S22 and figures S11 and S12 present post hoc analyses investigating the cause of greater best corrected visual acuity loss in the SRT group at years 3 and 4. Year 4 follow-up occurred for most participants during covid-19 lockdown (80%, 330/411), but best corrected visual acuity was only slightly worse in these participants (supplementary table S18), suggesting the lockdown did not explain the group differences. At year 4, the presence of reading centre determined subfoveal fibrosis, atrophy, ellipsoid zone disruption, other co-existing macular disease, or larger total and active lesion areas (using the median as a discriminator) appeared to be associated with greater best corrected visual acuity loss in SRT treated eyes, and supplementary figures S11 and S12 suggest the presence of atrophy and ellipsoid zone disruption at year 4 had a greater impact in the SRT group than in the sham SRT group. But even in those participants without these features, the results still favoured the sham SRT group, suggesting none of these individual factors could fully explain the reduced best corrected visual acuity in the SRT group. The vision outcome in participants with artificial intraocular lenses at baseline also favoured the sham SRT group (supplementary figure S8), and those without substantial lens opacity at year 4 still did worse with SRT treatment, suggesting subclinical lens opacity could not explain the difference between groups.

Systemic safety events were similar between groups. Slightly more deaths, non-serious blood and lymphatic disorders, and vascular disorders occurred in the SRT group, but these differences were small (supplementary table S24). Of the adverse events of special interest, systemic arterial thrombotic events occurred in 4% (11/276) of the SRT group and 5% (6/133) of the sham SRT group. Microvascular abnormalities or suspected radiation retinopathy were reported by investigators in 13% (35/276) of SRT treated eyes, and in no sham SRT treated eyes (supplementary table S23).

Overall, 24% (66/276) of SRT treated eyes had a serious adverse event, compared with 14% (18/133) of sham SRT treated eyes (supplementary table S23). This difference was mainly due to the microvascular abnormalities detailed previously, and slightly more cataracts in the SRT group eyes (14% (38/276) *v* 10% (14/133)). Of eyes treated in the SRT group, 5% (13/276) underwent laser capsulotomy and 2% (6/276) had macular haemorrhage, versus none of the eyes in the sham SRT group.

The reading centre determination of microvascular abnormalities and their effect on change in ETDRS best corrected visual acuity from baseline showed a trend to greater mean ETDRS best corrected visual acuity loss in SRT treated eyes with microvascular abnormalities compared with sham SRT treated eyes with microvascular abnormalities. However, there were low numbers of microvascular abnormalities in sham SRT treated eyes (16%, 16/102) versus SRT treated eyes (58%, 126/218). Only 24% (52/218) of microvascular abnormalities in SRT treated eyes and 5% (5/102) in sham SRT treated eyes involved the fovea (supplementary table S27).

### Costing analysis

Over the four year follow-up, the SRT group had lower costs for anti-VEGF and its administration (£13 819 *v* £15 093; supplementary table S3): a difference of -£1274 (95% CI -£3246 to £580; P=0.15). Monitoring consultation costs were similar between treatment groups (P=0.63). In the four year analysis population, the total treatment costs (inclusive of SRT, anti-VEGF, and monitoring) were £62 lower in the SRT group (95% CI -£2221 to £1980; P=0.95).

## Discussion

Despite exploratory analyses showing a persistent reduction in the frequency of anti-VEGF injections favouring SRT to year 4, the finding of worse vision with SRT effectively reverses the clinical conclusions of the year 2 analysis. Consequently, the longer term findings no longer support the use of SRT for chronic, active neovascular AMD.

At year 2, STAR showed a statistically significant reduction in anti-VEGF therapy, with 2.9 fewer injections than sham SRT.^11^ This benefit increased only slightly by year 4, to 3.2 fewer injections, indicating that most of the benefit occurred on trial within the first two years and not during routine clinic follow-up.

Even after the return of participants to standard of care, a cost saving of £62 per participant remained over four years.

At year 2, vision in the SRT group was non-inferior to vision in the sham SRT group at the prespecified five letter margin (-1.7 letters, 95% CI -4.2 to 0.8), but by year 4 it was 8.3 letters worse than with sham SRT (95% CI -12.7 to -4.0). This difference is less than the natural variability that occurs when people with neovascular AMD have their vision tested with the ETDRS eye chart, in that the 95% CI of test-retest variability is reported as 9-17 letters.^19^ However, it is greater than the reported minimum clinically important difference of 7.5 letters,^20^ suggesting this difference is enough to have an impact on patients if it occurs in their better seeing eye. The year 4 results reverse the positive best corrected visual acuity conclusion of our year 2 findings, which suggested SRT offered the prospect of fewer injections without disadvantaging vision.

### Possible reasons for vision loss during long-term follow-up

The reason for the worse vision with longer follow-up in a standard care setting is not clear. Subretinal fluid volume was greater in the SRT group at year 4, and it is possible that relative undertreatment in the SRT group during standard care might be contributory, as under-treatment has been linked to worse vision outcomes.^21^ Under-treatment might be more likely in the SRT group if participants and clinicians had built up an expectation of fewer injections during years 1 and 2. Arguing against this possibility, at year 3 the SRT group had marginally less subretinal fluid than the sham SRT group, but best corrected visual acuity was already 6.5 letters worse than with sham SRT at that stage (95% CI of difference -10.2 to -2.8). Another structural explanation might relate to macular atrophy, yet the neuroretina tended to be thicker in the SRT group at year 4 (supplementary table S15).

It is possible that the greater occurrence of microvascular abnormalities in the SRT group may partly explain the difference in best corrected visual acuity. Contrary to the year 2 findings, SRT treated participants with microvascular abnormalities at year 4 tended to do worse than SRT treated participants without microvascular abnormalities (supplementary table S27). But even in the group without microvascular abnormalities, best corrected visual acuity tended to favour the control group (albeit to a lesser extent), suggesting microvascular abnormalities cannot fully explain the difference. This is similar to the findings of the Macular EpiRetinal brachytherapy versus ranibizumab (Lucentis) Only Treatment (MERLOT) randomised controlled trial of epimacular brachytherapy for chronic active neovascular AMD.^8^ With extended standard care follow-up to year 3, the radiotherapy group had worse best corrected visual acuity than the sham treated group, but there was no material difference between participants with or without microvascular abnormalities in the radiotherapy group (-21.8 *v* -19.4 letters, P=0.65).^22^

Another possibility is that SRT did not fully encompass larger lesions, which tended to do worse in the SRT compared with sham SRT group. This was also observed in the INTREPID study,^23^ and this finding underpinned our decision to only include lesions <4 mm in diameter, as these were thought to fit fully within the 4 mm macular treatment zone (which receives at least 90% of the intended radiation dose). It is possible, however, that subtle eye malposition occurred despite the suction coupled contact lens and eye tracking software, resulting in incomplete irradiation at the margin of larger lesions, or that the 90% radiation isodose area was smaller than calculated. Alternatively, an unidentified biological reason might explain why larger lesions respond differently to SRT.

A final possibility is that radiation caused subclinical lens opacity that reduced vision in the SRT group, but this seems unlikely as the year 4 vision outcome in participants with implanted lenses at baseline still favoured the sham treated group (supplementary figure S8).

### Relation between visual acuity and patient reported outcome measures

There are several potential reasons why the difference in best corrected visual acuity between groups did not result in differences in the NEI-VFQ-25 composite score or the EQ-5D-5L utility responses, either with or without an additional (bolt-on) question concerning vision. The most likely is that these instruments are relatively insensitive to the observed differences in visual function. In two earlier trials on anti-VEGF therapy in neovascular AMD, the least squares mean change in NEI-VFQ-25 composite score for participants who lost 15 or more letters was between 1.2 (95% CI -2.1 to 4.5) and 6.3 points (95% CI 3.9 to 8.6).^24^ No published responsiveness studies have been undertaken for the EQ-5D-5L with vision bolt-on in neovascular AMD to the authors’ knowledge; an analysis of responsiveness of both VFQ-25 and EQ-5D-5L to visual acuity in the STAR trial is reported in a separate, full health economic analysis publication.^25^ Another reason may be because most study eyes (63%, 260/411) were the worse seeing eye at baseline, and despite both groups losing vision, these participants only experienced minimal reductions in NEI-VFQ-25 composite score throughout the trial, compared with greater reductions in participants whose study eye was the better seeing eye (supplementary table S16). This could suggest that better seeing, fellow, non-study eyes were supporting overall visual function. This is consistent with the common clinical observation that patients may be asymptomatic when one eye loses vision, if the other retains good vision, as the overall vision with both eyes open remains satisfactory. An alternative explanation might be that the difference in best corrected visual acuity between groups is not of sufficient magnitude to materially reduce visual function when both eyes are being used. Studies designed to validate the VFQ-25 found that the correlation between the composite score and best corrected visual acuity while statistically significant was still only moderately well correlated with the better seeing eye, and only weakly correlated with the worse seeing eye (correlation coefficient 0.49 and 0.21, respectively), and meta-analyses of submacular surgery trials found that change of visual acuity in the worse seeing eyes of participants did not correlate with any meaningful change in NEI-VFQ-25 scores.^18^ ^26^

### Dose selection

In this trial we selected 16 Gry SRT, as that provided similar efficacy to 24 Gry in the INTREPID study, but the lower dose may provide a greater radiation safety margin. It is not known if repeat dosing at, for example, year 2 might extend the reduction in anti-VEGF dosing frequency, but repeat dosing needs to consider the risk of radiation retinopathy, which is more common above about 45 Gry.^27^

### Results in men versus women

The reason for the better response to SRT in men than women is uncertain. If men respond worse to anti-VEGF therapy, an alternative treatment may have a relative advantage. Some retrospective evidence suggests that men do respond worse to anti-VEGF monotherapy than women, in terms of both macular anatomy^28^ and vision,^29^ but the effect size may be as little as 0.33 letters.^30^ Women have better survival after radiotherapy for cancer, but they also experience greater toxicity.^31^ Ischaemia may contribute to AMD,^32^ and men have worse cardiovascular disease,^33^ but if anything this predicts an adverse response to SRT, if SRT causes capillary drop-out that adds to pre-existing vascular compromise. It is possible,^34^ ^35^ but not certain,^35^ ^36^ ^37^ that men have a thicker choroid, and a thicker choroid may be associated with a better response to SRT for neovascular AMD.^38^ Men’s sex hormone levels decrease later in life than women’s, which may explain why the age related decline in choroidal thickness lags by about 10 years compared with women,^35^ and women live longer. Men may also have more vortex veins,^39^ a difference in the lumen:interstitial ratio, and a different topographic distribution of choroidal thickness.^40^ Inflammation contributes to AMD and radiation can be anti-inflammatory. In general, women tend to have greater antibody production through the B cell humoral response, and a greater helper T cell 2 response, compared with men.^41^ Additionally, sex hormones are known to modulate immune function and gene expression, with androgens reducing immunocompetence.^41^ Radiation can be antifibrotic, but the female immune response is more generally associated with chronic fibrosis in other body organs.^41^ Despite these speculations, there may be important confounding factors, as not all of these observations predict a better response in men, and the findings in other diseases may not be translatable to neovascular AMD.

### Strengths and limitations of this trial

Strengths of this trial include its randomised, double masked, sham controlled design, with long term, masked, standard care follow-up. The real world data collected after the primary outcome, when participants reverted to standard care, offer more generalisable findings than on trial results. The compliance cube, designed in the context of the covid-19 pandemic, presents a novel way to report trial adherence. It used predefined rules, agreed by an independent trial steering committee, to categorise each participant’s visit deviations on their likelihood of affecting the main outcome. Hence the diagram offers an objective way to report adherence across each participant’s journey through the trial, and across the course of the trial. Even in the highest ranked medical journals, adherence to the protocol is often poorly reported,^42^ yet it underpins the results. The compliance cube may be useful for other trials.

Limitations include the 20% loss to follow-up, but an average of 5% attrition yearly might be considered favourable, particularly as the covid-19 lockdown affected the trial. The primary outcome was at year 2, therefore the year 4 efficacy results are exploratory. It is possible that results may differ with longer follow-up, but four years is already a long study relative to others on this topic,^9^ ^12^ ^22^ and in studies of higher dose radiation, retinopathy was most likely to emerge within about 2-3 years, so four years provides a reasonable chance of detecting damage. The covid-19 pandemic had the potential to alter the primary outcome and best corrected visual acuity if there was under-treatment, but our relatively detailed sensitivity analyses supported the main conclusion. The better result in men than women is interesting and may point to a biological difference in response that deserves further investigation, but this was a post hoc analysis prompted by the peer review process of the primary outcome paper and therefore needs to be interpreted cautiously. The proposed explanations for the worse long term best corrected visual acuity in the SRT group and in women are speculative, and further research may yet provide greater mechanistic insight, but further interventional trials of this particular SRT device do not seem justified.

### Policy implications

This trial shows the advantage of both long term and standard care masked follow-up after the primary outcome, as part of the intentional trial design. This design is not included in many randomised controlled trials of novel drugs and devices, in favour of post-market surveillance studies, yet the flaws of post-market research are well known, including under-reporting of adverse events and limited or biased efficacy reporting.^43^ ^44^ ^45^ While not appropriate for all studies, for some it would be feasible to require sponsors to fund standard care masked follow-up if the primary outcome was positive, as that would often lead to a marketing authorisation and revenue stream, and the added cost of a late recall visit is small relative to the total cost of a trial.

### Conclusion

The results of this trial in a standard care setting do not support the use of SRT to treat neovascular AMD, as the positive year 2 findings were not replicated at year 4. The reason for worse best corrected visual acuity after SRT is uncertain and warrants further investigation.

**What is already known on this topic**

Neovascular age related macular degeneration (AMD) is a leading cause of blindness

Intravitreal injections of anti-vascular endothelial growth factor (anti-VEGF) drugs are used to treat neovascular AMD

Stereotactic radiotherapy (SRT) precisely delivers radiation to the macula and reduces the number of anti-VEGF injections needed to treat neovascular AMD over two years, without disadvantaging vision

**What this study adds**

This trial found that over four years, SRT treated eyes still require fewer injections, but the main benefit was derived in the first two years of treatment

SRT treated eyes lost more vision over time, exceeding the non-inferiority margin of five letters

This difference would not have been observed had the trial stopped at its primary outcome of two years, therefore masked, long term, standard care follow-up is important for some trials

## Data Availability

Data sharing: The code used to analyse the data in the trial is openly available at: https://github.com/HatemWafa/star-trial.git. The data underlying the findings in this paper are openly and publicly available from the UK Data Service repository at: https://reshare.ukdataservice.ac.uk/858332/. Use of the data is restricted to non-commercial purposes in accordance with the repository license. If you encounter problems accessing the data, please contact the corresponding author.

## Web extra

: Extra material supplied by authors

## Notes

Contributors: TLJ was the chief investigator, led the bid for funding, wrote the protocol, conceived the compliance cube, and co-prepared the first draft of the manuscript. RD was the trial manager throughout. LR is the current associate trial manager and trial monitor. YW and JP were co-applicants on the bid for funding and designed the statistical analysis plan. HAW executed the statistical analysis plan and additional sensitivity analyses and post hoc analyses under the supervision of YW and JP. HAW constructed the compliance cube. UC was a clinical co-applicant on the bid for funding and directed the reading centre. TP led the reading centre image analysis. XZ, HD, and SW designed the costing analysis. PC was the lead medical physicist who took over from the preceding lead medical physicist (Cornelius Lewis). JEN was the trial’s clinical research fellow and led the source document design and preparation, followed by CNL as clinical research fellow, who co-prepared the first draft of the manuscript, led on post hoc analyses, and coordinated and executed the manuscript revisions. JMOS was the clinical radiation expert and co-applicant on the bid for funding. BCR was the deputy chief investigator, trial methodologist, and co-applicant on the bid for funding. All authors reviewed and critiqued the manuscript and were responsible for the decision to submit the manuscript for publication. HAW and YW had access to and verified the data, following data cleaning by LR, RD, and CNL. XZ undertook the costing analysis under supervision of SW and HD, who had access to the data. TLJ is the guarantor. The corresponding author attests that all listed authors meet authorship criteria and that no others meeting the criteria have been omitted.

Transparency: The lead author (the manuscript’s guarantor) affirms that the manuscript is an honest, accurate, and transparent account of the study being reported; that no important aspects of the study have been omitted; and that any discrepancies from the study as planned (and, if relevant, registered) have been explained.

Dissemination to participants and related patient and public communities: A lay summary of the trial results has been provided to principal investigators at each site, to distribute to trial participants who would like to know the trial outcomes.

Provenance and peer review: Not commissioned; externally peer reviewed.
