# Supplementary material for: Stereotactic radiotherapy for neovascular age related macular degeneration: year 3 and 4 extended follow up results of a randomised, double masked, sham controlled, device trial (STAR)
Source: BMJ. 2026 Jun 18;393:e729694. doi: 10.1136/bmj-2026-729694 (PMC13327491; doi:10.1136/bmj-2026-729694)
Supplement: Supplementary file 1 — Supplementary information: Appendix [file jact-2026-05-27-729694-datasupp1.pdf]

Appendix

Supplementary material for *Stereotactic radiotherapyy for neovascular age-related macular degeneration (STAR): year 3 and 4 results of a pivotal, randomised, double-masked, sham-controlled device trial.*

Contents page

1. Eligibility criteria 4-5
2. Schedule of assessments 6
3. Statistical analysis plan (SAP)* 7-28
4. Post-hoc analysis plan 29
5. Table S1: Explanatory causes of vision loss 29

Health economic analysis

6. Additional methods of costing analyses 30-31
7. Table S2: Mean cost of neovascular age-related macular degeneration treatment (UK 2021-22 pounds) 31-32
8. Costing analysis: Results 32
9. Table S3: STAR-study health economic unit costs 32
10. Table S4: Number of participants selecting each anti-VEGF drug in years 3 and 4 (available cases) 32
11. Health economic analysis: References 33

Missingness and adherence

Missingness

12. Table S5: Missingness 34
13. Table S6: Reasons for missingness 35

Adherence

14. Table S7: Classification of visit adherence (called 'compliance' in the finalised SAP version) 35-36

Other secondary outcomes

15. Table S8: Outcomes of EQ-5D-5L domains by treatment groups at different visit weeks 37

Sensitivity outcomes

16. Table S9: Sensitivity analyses of missing data and COVID pandemic impact: primary outcome at week 48 38
17. Table S10: Sensitivity analyses of missing data and COVID pandemic impact: primary outcome at week 96 39

18. Table S11: Sensitivity analyses of missing data and COVID pandemic impact: primary outcome at week 144 40

19. Table S12: Sensitivity analyses of missing data and COVID pandemic impact: primary outcome at week 192 41

Clinical efficacy outcomes

Primary and secondary outcomes by sex

20. Table S13: Primary and secondary efficacy outcomes at week 144 and week 192 by sex 42-43

Number of injections

21. Table S14: Mean and cumulative number of anti-VEGF injections per 4-weekly visit 43

22. Figure S1: Mean number of anti-VEGF injections for each 4-weekly visit 44

23. Figure S2: Number of anti-VEGF injections by participants 45

Visual acuity

24. Figure S3: Mean visual acuity over time 46

Macular thickness

25. Figure S4: Optical coherence tomography central subfield thickness over time 47

Reading-centre imaging outcomes

26. Table S15: Reading centre-determined optical coherence tomography analysis 48

Subgroup and post-hoc analyses

27. Figure S5: Difference in number of injections by subgroup at week 144 49

28. Figure S6: Difference in number of injections by subgroup at week 192 50

29. Figure S7: Difference in final visual acuity by subgroup at week 144 51

30. Figure S8: Difference in final visual acuity by subgroup at week 192 52

31. Figure S9: Difference in final optical coherence tomography central subfield thickness by subgroup at week 144 53

32. Figure S10: Difference in final optical coherence tomography central subfield thickness by subgroup at week 192 54

33. Table S16: Outcomes of visual acuity and VFQ-25 composite scores by study eye being better/worse seeing eye at baseline 55

34. Table S17: Outcomes of visual acuity and VFQ-25 composite scores by completeness of outcomes during COVID-19 lockdown 55

35. Table S18: Outcomes of visual acuity and VFQ-25 composite scores by presence of visually significant lens opacity 55-56

36. Table S19: Outcomes of visual acuity and VFQ-25 composite scores by presence of subfoveal fibrosis, subfoveal atrophy, and ellipsoid zone disruption 56-57

37. Table S20: Outcomes of visual acuity and VFQ-25 composite scores by presence of active exudative AMD, total lesion area, and total active lesion area 57

38. Table S21: Outcomes of visual acuity and VFQ-25 composite scores by median total lesion area, and total active lesion area 57-58

39. Table S22: Outcomes of visual acuity and VFQ-25 composite scores by presence of macular co-pathology 58

40. Figure S11: Differences in ETDRS best-corrected visual acuity in SRT group study eyes disaggregated by explanatory causes for vision loss, at each timepoint. 59

41. Figure S12: Differences in ETDRS best-corrected visual acuity in sham SRT group study eyes disaggregated by explanatory causes for vision loss, at each timepoint. 60

Safety

Adverse events

42. Table S23: Adverse events and serious adverse events in the study eye by received treatment over 192 weeks 61

43. Table S24: All adverse events and serious adverse events by received treatment over 192 weeks 62-63

44. Table S25: Intensity and relatedness of adverse events in the study eye over 192 weeks 64

45. Table S26: Intensity and relatedness of adverse events over 192 weeks 65

Microvascular abnormalities and impact on acuity

46. Table S27: Mean change in best-corrected visual acuity over 192 weeks by reading center-determined microvascular abnormality status and fovea involvement 66

Additional tables

Baseline characteristics of participants disaggregated by missingness at final outcome

47. Table S28: Baseline characteristics of participants with and without complete primary outcome data at follow-up 67-68

Baseline characteristics of participants disaggregated by sex

48. Table S29: Baseline characteristics of participants disaggregated by sex 68-69

List of collaborators, committee members, national treatment centres and reading centre staff

49. Study Investigators 70

50. National Treatment Centres
Lead clinician 70
Lead medical physicist or radiation protection officer 70

51. Trial Steering Committee members 70-71

52. Data Monitoring and Ethics Committee members 71

53. Study Reading Centre members 71

* Note that "compliance" was the term used in the finalised SAP (pp7-28) to refer to whether participants adhered to the trial protocol. This has been changed to "adherence" in this publication and appendix in all sections apart from the SAP which remains the original finalised version.

1. **Eligibility criteria**

Inclusion Criteria

- Participants must have neovascular AMD in the study eye, for which they have received at least 3 prior intravitreal injections of either bevacizumab (Avastin), aflibercept (Eylea), ranibizumab (Lucentis), or pegaptanib (Macugen).

- Participants must have received an anti-VEGF injection in the study eye within 4 months prior to enrolment.

- Participants must require treatment with anti-VEGF therapy at the time of enrolment, due to OCT evidence of subretinal fluid and/or cystoid macular oedema, **and** have a macular volume that is greater than **a pre-defined threshold** that varies for each different make of SD-OCT machine. The threshold for each approved machine is shown in the protocol.

- Participants must be at least 50 years of age.

Exclusion Criteria

- Disciform scarring that involves the fovea, in the study eye.

- Visual acuity worse than 6/96 (24 ETDRS letters) in the study eye.

- Lesion size greater than 4 mm in greatest linear dimension, or greater than 2 mm from the centre of the fovea to the furthest point on the lesion perimeter, to include active choroidal neovascular leakage, pigment epithelial detachment and haemorrhage, as determined by fluorescein angiography.*

- An axial length of less than 20 mm, or greater than 26 mm, in the study eye.

- Contraindication or sensitivity to contact lens application, including recurrent corneal erosions, in the study eye.

- Type 1 or Type 2 diabetes mellitus.

- Retinopathy in the study eye.

- Prior, current or anticipated treatment in the study eye for age-related macular degeneration, other than anti-VEGF agents, including submacular surgery, subfoveal thermal laser photocoagulation, photodynamic therapy (PDT), or transpupillary thermotherapy (TTT).

- Presence of an intravitreal device in the study eye.

- Previous radiation therapy to the study eye, head, or neck with the exception of radio-iodine treatment for hyperthyroidism, epimacular brachytherapy to the non-study eye, or IRay SRT to the non-study eye.

- Inadequate pupillary dilation or significant media opacities in the study eye, including cataract, which may interfere with visual acuity testing, the clinical evaluation of the posterior segment, or fundus imaging.

- Study eyes with CNV due to causes other than AMD, including presumed ocular histoplasmosis syndrome (POH), angioid streaks, multifocal choroiditis, choroidal rupture, and pathological myopia (greater than 8 Dioptres spherical equivalent). Participants with retinal angiomatous proliferation (RAP) or idiopathic polypoidal choroidal vasculopathy (IPCV) are *not* excluded.

- Known allergy to intravenous fluorescein, ICG or intravitreal ranibizumab.

- Intraocular surgery or laser-assisted in situ keratomileusis (LASIK) in the study eye within 12 weeks prior to enrolment.

- Prior pars plana vitrectomy in the study eye.

- Current participation in another interventional clinical trial, or participation in such a clinical trial within the last six months.

- Unwilling, unable, or unlikely to return for scheduled follow-up for the duration of the trial.

- Women who are pregnant at the time of radiotherapy.

- Participants with an implantable cardioverter defibrillator (ICD) or pacemaker implant (or any implanted device) where the device labelling specifically contraindicates patients undergoing X-ray.

- Any other condition, which in the judgment of the investigator, would prevent the participant from granting informed consent or completing the study, such as dementia, and mental illness (including generalized anxiety disorder and claustrophobia).

* One of the key exclusion criteria is the lesion size and distance from the centre of the fovea to the furthest point on the lesion perimeter. This assessment is done for the study eye on the fluorescein angiogram, at screening.

The measurement relates to the area of active leakage on fluorescein angiography. The measurement should be taken on an early phase of angiography. The measurement includes active CNV leakage, pigment epithelial detachment and haemorrhage that is contiguous with the active leakage. Atrophy, inactive fibrosis, RPE tears and haemorrhages that are not related to the area of active leakage are not included in the measurement.

Two separate measurement must be performed:
- greatest linear dimension (GLD) - the maximum diameter of the area of active leakage defined as above; in order for the patient to be eligible for the study, this must not exceed 4 mm; the GLD must be recorded in the source documents.
- distance from the centre of the fovea to the furthest point on lesion perimeter (same lesion as above) - in order for the patient to be eligible for the study this must be less than 2 mm.

If the area of the lesion is uncertain on fluorescein angiography, OCT can be used to help determine the active leakage, but the final measurement must be taken on fluorescein angiography.

The greatest linear dimension is also measured and recorded in the source documents at month 12 and month 24 visits.

2. **Schedule of assessments**

Assessment	Screening	SRT with baseline ranibizumab	Monthly review (Month 1-11)	Month 12	Monthly review (Month 13-23)	Month 24	Month 36	Month 48
Visit Window: *Day 0 = Day of successful enrolment*	**Day -14 to 0**	**Day 0 to 21**	**±7 days**	**±7 days**	**±7 days**	**±7 days**	**±14 days**	**±14 days**
Informed Consent	**X**							
Demographics	**X**							
Ophthalmic History	**X**							
Med History/Con Meds	**X**							
Blood Pressure	**X**							
EDTRS Visual Acuity	**X**		**X**	**X**	**X**	**X**	**X**	**X**
Intraocular Pressure	**X**			**X**		**X**	**X**	**X**
Cataract Assessment	**X**			**X**		**X**	**X**	**X**
Biometry	**X**							
OCT (sent to reading centre)	**X**			**X**		**X**	**X**	**X**
OCT (*not* sent to reading centre)	**X**		**X**		**X**			
Fundus Photographs (sent to reading centre)	**X**			**X**		**X**	**X**	**X**
Fluorescein Angiography (sent to reading centre)	**X**			**X**		**X**	**X**	**X**
Indocyanine Green Angiography (sent to reading centre)	**X**							
Stereotactic Radiotherapy with mandated baseline Ranibizumab		**X**						
Ranibizumab injection if required (*prn*)			**X**	**X**	**X**	**X**	**X**	**X**
Health Economics questionnaires			**X**	**X**	**X**	**X**	**X**	**X**
EQ-5D and VFQ-25 patient questionnaires	**X**			**X**		**X**	**X**	**X**
AEs/ConMed changes			**X**	**X**	**X**	**X**	**X**	**X**

3. **Statistical analysis plan**

STAR Trial

StereoTactic radiotherapy for wet Age-Related macular degeneration (STAR): A randomised, double-masked, sham-controlled, clinical trial comparing low-voltage X-ray irradiation with as needed ranibizumab, to as needed ranibizumab monotherapy.

Statistical Analysis Plan Version 1·4

Version 1·4 started: 14/04/2022

ISRCTN: 12884465

Prepared by:

Hatem Wafa Statistician

School of Life Course & Population Health Sciences

King's College London Addison House, Guy's Campus London, SE1 1UL

Tel: 020 7848 6623

Approved by:

Email: hatem.a.wafa@kcl.ac.uk

Signature: Hatem Wafa

Yanzhong Wang

Reader in Medical Statistics

School of Life Course & Population Health Sciences

King's College London Addison House, Guy's Campus London, SE1 1UL

Tel: 020 7848 8223

Email: yanzhong.wang@kcl.ac.uk

Date: 29 April 2022

Signature:

Date: 29 April 2022

CONTENTS

This document contains up to date statistical analysis plans (with version numbers and dates).

A) Quantitative Analysis Plan
B) Economic Analysis Plan
C) Schedule of Assessments and Measures

A) *QUANTITATIVE ANALYSIS PLAN* *95*
1. Description of the trial 96
1.1 Principal research objectives to be addressed 96
Primary objectives 96
Secondary objectives 96
1.2 Trial design and flowchart 96
Figure 1. Trial design flow diagram 97
1.3 Method of allocation of groups 98
1.4 Study duration and frequency of follow up 98
1.5 Data collection 98
1.5.1 Eligibility screening 98
Exclusion Criteria 98
1.5.2 Efficacy Measures 99
Primary Measure 100
Secondary Measure 100
1.5.3 Safety Outcome Measures 100
1.6 Sample size estimation (including clinical significance) 100
Summary 100
Justification for parameters used in the sample size calculations 101
1.7 Brief description of proposed analyses 101
2. Data analysis plan – Data description 102
2.1 Recruitment and representativeness of recruited patients 102
2.2 Baseline comparability of randomised groups 102
2.3 Loss to follow-up on outcome data 102
2.4 Adverse event reporting 102
2.5 Descriptive statistics for outcome measures 102
Primary Measure 103
Secondary Measure 103
3. Data analysis plan – Inferential analysis 103
3.1 Main analysis of treatment differences 103
3.1.1 Analysis of primary outcomes 103
3.1.2 Analysis of secondary outcomes 104
3.1.3 Planned subgroup analyses 104
3.1.4 Statistical considerations 105
3.2 Exploratory analyses 106
3.3 Interim analysis 106
4. Software 106
B) SCHEDULE OF ASSESSMENTS AND MEASURES 107
Appendix A: Compliance rules 108
Reference List 112

A) QUANTITATIVE ANALYSIS PLAN

Chief Investigator

Timothy L Jackson PhD, FRCOphth

Professor of Retinal Research

Consultant Ophthalmic Surgeon

Department Ophthalmology

King's College Hospital

London SE5 9RS

Tel: 020 3299 1297

Fax: 020 3299 1721

Email: t.jackson1@nhs.net

Trial Statistician

Hatem Wafa, MPH

Statistician

King's College London

Addison House, Guy's Campus

London SE1 1UL

Tel : 020 7848 6620

Email: hatem.a.wafa@kcl.ac.uk

Deputy Chief Investigator
Barney Reeves DPhil (Oxon), MFPHM
Professorial Fellow, Health Services Research
Clinical Trials and Evaluation Unit

Bristol Royal Infirmary, Level 7

Bristol BS2 8HW

Senior Trial Statistician
Wang Yanzhong, PhD
Reader in Medical Statistics
King's College London

Addison House, Guy's Campus

London SE1 1UL

Tel : 020 7848 8223

Email: yanzhong.wang@kcl.ac.uk

KCTU manager

Ms Caroline Murphy

King's Clinical Trials Unit, PO64,

Institute of Psychiatry,

De Crespigny Park, Denmark Hill,

London, SE5 8AF
Tel: 020 7848 5273 Fax: 020 7848 5229

Email: caroline.murphy@kcl.ac.uk

Trial data manager

Ms Joanna Kelly

King's Clinical Trials Unit, PO64,

Institute of Psychiatry,

De Crespigny Park, Denmark Hill,

London, SE5 8AF
Tel: 020 78480532

Email: Joanna.kelly@kcl.ac.uk

Trial investigators:
Timothy Jackson (CI), Barney Reeves, Yanzhong Wang, Cornelius Lewis, Sarah Wordsworth, Usha Chakravarthy, Hatem Wafa.

1. Description of the trial

The key objective of the STAR study is to evaluate the safety and efficacy of low voltage external beam radiotherapy, in combination with anti-VEGF therapy, for the treatment of neovascular (wet) AMD. Specifically, this study will evaluate whether Stereotactic RadioTherapy (SRT) reduces the need for ranibizumab injections, compared with ranibizumab monotherapy. STAR will also determine if SRT produces a non-inferior visual outcome compared with anti-VEGF monotherapy.

This study aims to enrol 411 participants in a double-masked, multicentre, sham-controlled clinical trial.

Participants will receive a single treatment of SRT using the iRay system Gray) with a concomitant baseline intravitreal injection of 0.5 mg ranibizumab. Thereafter, participants will attend clinic for a review every month (28 days) for 96 weeks*prn*). Two safety visits occur subsequently, one at 36 months (144 weeks) and the other at 48 months (192 weeks).

1.1 Principal research objectives to be addressed

Hypothesis: For patients with neovascular (wet) age-related macular degeneration (AMD), 16 Gray SRT, together with as needed, intravitreal, anti- vascular endothelial growth factor (VEGF) therapy, results in fewer intravitreal anti-VEGF injections and a non-inferior visual acuity outcome, compared with anti-VEGF monotherapy.

Aim: To determine the safety and efficacy of 16 Gray SRT.

Primary objectives

To investigate:

- If 16 Gray SRT, in conjunction with anti-VEGF therapy, reduces anti-VEGF injection frequency.

Secondary objectives

To investigate:

- If 16 Gray SRT, in conjunction with anti-VEGF therapy, achieves a visual acuity outcome that is not inferior to anti-VEGF monotherapy.
- If 16 Gray SRT is safe.
- If 16 Gray SRT is cost-effective.
- If there are baseline structural features associated with a poor response to anti-VEGF therapy and a positive response to SRT.
- If retinal microvascular changes occur in response to SRT, and if so, how do these evolve over time?

1.2 Trial design and flowchart

Randomised, double-masked, sham-controlled, multicenter, clinical trial.

Figure 1. Trial design flow diagram

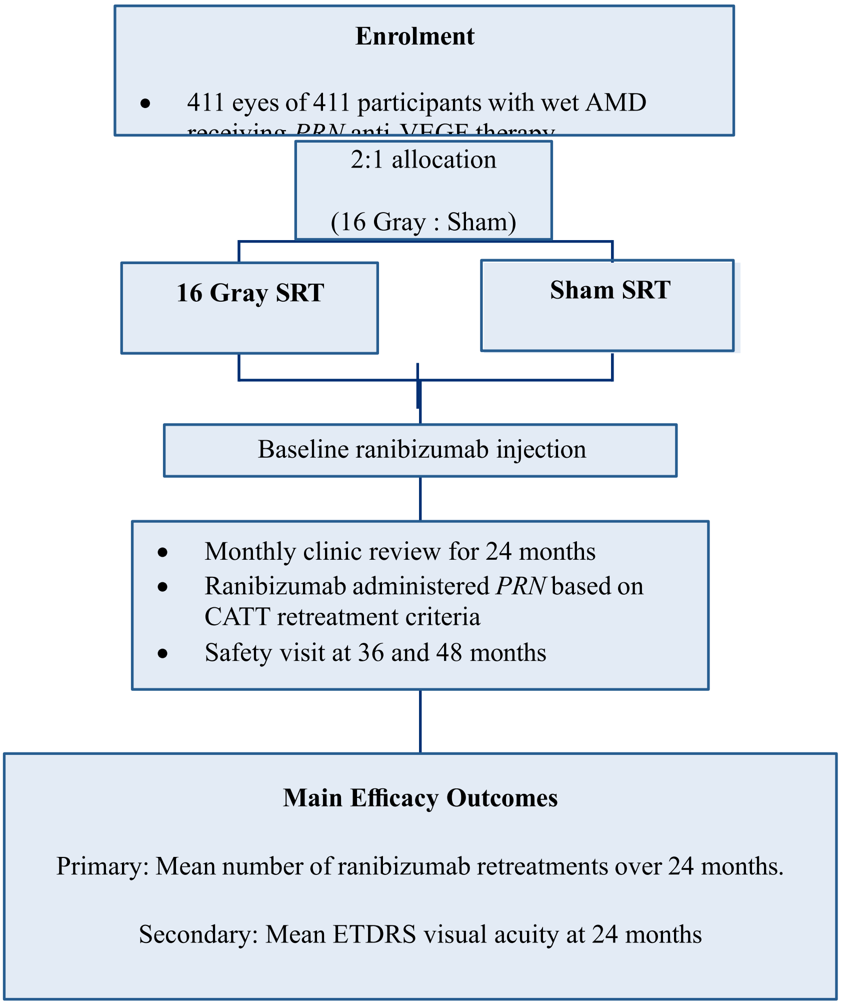

Abbreviations: AMD, age-related macular degeneration; CATT, comparison of AMD treatments trial; ETDRS, early treatment of diabetic retinopathy study; *PRN*, *pro re nata* ‘as required’ dosing; SRT, stereotactic radiotherapy; VEGF, vascular endothelial growth factor.

1.3 Method of allocation of groups

Once baseline assessments are complete, participants will be randomized to SRT and sham in a 2:1 ratio. Randomisation is at the patient level and is performed using an online randomisation system set up by the King's Clinical Trials Unit (KCTU) at King's College London. Randomisation is stratified by national treatment centre with variable block sizes to ensure that patients are allocated to the two arms within each treatment centre in a 2:1 ratio. The procedure is as follows: The patient travels from their local recruiting site having been determined as eligible. Staff at the national treatment centre then use the online randomisation system to get an alphanumeric code. This is entered into the iRay system, and that will administer sham treatment or active treatment. The person delivering radiation/sham does not know which has been selected, as the machine fires up and prepares a dose map in the same way for each treatment.

1.4 Study duration and frequency of follow up

Participants will be treated at baseline and followed every 28 days for 24 months, with safety visits at month 36 and month 48.

1.5 Data collection

The trial will randomize 411 patients with previously treated, wet AMD. After giving fully informed written consent, patients with wet AMD will be screened for participation in the study. For patients with two eligible eyes, the patient may select which eye they wish to allocate as the study eye. Patients should fulfil the following criteria to be eligible for enrolment:

1.5.1 Eligibility screening

Key Eligibility Criteria

- Males and females with wet AMD requiring anti-VEGF treatment at the time of entry to the study.

In details:

Inclusion Criteria

- Participants must have neovascular AMD in the study eye, for which they have received at least 3 prior intravitreal injections of either bevacizumab (Avastin), aflibercept (Eylea), ranibizumab (Lucentis), or pegaptanib (Macugen).
- Participants must have received an anti-VEGF injection in the study eye within 3 months prior to enrolment.
- Participants must require treatment with anti-VEGF therapy at the time of enrolment, due to optical coherence tomography (OCT) evidence of subretinal fluid and/or cystoid macular oedema, **and** a macular volume that is greater than the 95th percentile of normal for the Spectral Domain (SD)-OCT machines used in the investigational sites.
- Participants must be at least 50 years of age.

Exclusion Criteria

- Disciform scarring that involves the fovea, in the study eye.

- Visual acuity worse than 6/96 (24 ETDRS letters) in the study eye.
- Lesion size greater than 4 mm in greatest linear dimension, or greater than 2 mm from the centre of the fovea to the furthest point on the lesion perimeter.
- An axial length of less than 20 mm, or greater than 26 mm, in the study eye.
- Contraindication or sensitivity to contact lens application, including recurrent corneal erosions, in the study eye.
- Type 1 or Type 2 diabetes mellitus.
- Retinopathy in the study eye.
- Prior, current or anticipated treatment in the study eye for age-related macular degeneration, other than anti-VEGF agents, including submacular surgery, subfoveal thermal laser photocoagulation, photodynamic therapy (PDT), or transpupillary thermotherapy (TTT).
- Presence of an intravitreal device in the study eye.
- Previous radiation therapy to the study eye, head, or neck with the exception of radio-iodine treatment for hyperthyroidism, epimacular brachytherapy to the non-study eye, or SRT to the non-study eye.
- Inadequate pupillary dilation or significant media opacities in the study eye, including cataract, which may interfere with visual acuity testing, the clinical evaluation of the posterior segment, or fundus imaging.
- Likely to need cataract surgery in the study eye, within two years of enrolment.
- Study eyes with CNV due to causes other than AMD, including presumed ocular histoplasmosis syndrome (POH), angioid streaks, multifocal choroiditis, choroidal rupture, and pathological myopia (greater than 8 Dioptres spherical equivalent). Participants with retinal angiomatous proliferation (RAP) or idiopathic polypoidal choroidal vasculopathy (IPCV) are *not* excluded.
- Known allergy to intravenous fluorescein, indocyanine green (ICG) or intravitreal ranibizumab.
- Intraocular surgery or laser-assisted in situ keratomileusis (LASIK) in the study eye within 12 weeks prior to enrolment.
- Prior pars plana vitrectomy in the study eye.
- Current participation in another interventional clinical trial or participation in such a clinical trial within the last six months.
- Unwilling, unable, or unlikely to return for scheduled follow-up for the duration of the trial.
- Women who are pregnant at the time of radiotherapy.
- Participants with an implantable cardioverter defibrillator (ICD) or pacemaker implant (or any implanted device) where the device labelling specifically contraindicates patients undergoing X-ray.
- Any other condition, which in the judgment of the investigator, would prevent the participant from granting informed consent or completing the study, such as dementia, and mental illness (including generalized anxiety disorder and claustrophobia).

1.5.2 Efficacy Measures

The following outcomes will be reported at Month 24

Primary Measure

- Number of as required (*prn*) ranibizumab injections during the first 24 months.

Secondary Measure

- Mean ETDRS VA.
- Percentage of participants losing < 15 ETDRS letters
- Percentage of participants gaining ≥ 0 ETDRS letters
- Percentage of participants gaining ≥ 15 ETDRS letters
- Total lesion size by fluorescein angiography
- Total CNV size by fluorescein angiography
- Foveal thickness measured using OCT
- Health-related quality of life assessed using the National Eye Institute 25- Item Visual Function Questionnaire and the EuroQol EQ-5D™ questionnaire
- Cost per Quality Adjusted Life Year (QALY)

1.5.3 Safety Outcome Measures

Safety will be evaluated by assessing adverse events (AEs) and serious adverse events (SAEs). The trial will specifically report the incidence of radiation retinopathy or radiation-related microvascular changes, and arteriothrombotic events.

1.6 Sample size estimation (including clinical significance)

Summary

If SRT produces a 25% reduction, group sample sizes of 248 and 124 (ratio: 2:1) achieve 90% power to detect a difference of 2.5 injections between the null hypothesis that both group means are 10 injections and the alternative hypothesis that the mean of the active treatment group is 7.5 injections, with a standard deviation (SD) of 7 for both, and a significance level (alpha) of 0.05 (two-sided) using a two-sample t-test. A 2:1 ratio adds only 42 patients but boosts recruitment and safety data.

We expect VA in the SRT group to be non-inferior compared to the control group. The SD of the mean change in VA was estimated as 12 letters from the INTREPID study. Group sample sizes of 248 and 124 achieve 97% power to detect non-inferiority in the mean changes in VA using a one-sided, two-sample t- test assuming a SD of 12 for both groups. The margin of equivalence is 5 letters. The true difference between the means is assumed to be 0. The significance level (alpha) of the test is 0.025.

In the INTREPID study, 2.2% of the randomized population were lost to follow up by year 1. Year 2 data are not representative as INTREPID had minimal review in year 2. The CABERNET study had 93% of data available for analysis at the end of year 2. We anticipate a 10% loss to follow-up over two years for STAR, so we aim to recruit 274 participants in the active arm and 137 in the control arm (total 411). Sample size calculations were performed using PASS software.

Justification for parameters used in the sample size calculations.

The INTREPID study (ClinicalTrial.gov identifier: NCT01016873) compared patients treated with low-voltage x-ray, external-beam, SRT plus ranibizumab *prn* to patients treated with sham SRT plus ranibizumab *prn*. Since INTREPID studied anti-VEGF-experienced patients the results of that study are more relevant to the STAR population than the results of CATT, which studied anti-VEGF-naïve participants. Participants in INTREPID were randomized to 16 Gray plus ranibizumab *prn*, 24 Gray plus ranibizumab *prn*, or sham radiotherapy (either 16 Gray or 24 Gray) plus ranibizumab *prn*. The mean changes in ETDRS VA at 12 months (±SD) were -0.28 ± 8.77, 0.40 ± 10.33, and -1.57 ± 11.90, respectively. The pooled SD across all groups is therefore 10.4, with approximate 95% confidence limits of 9.6 and 11.5. For power calculations for STAR, the assumed SD of the mean change in VA is 12 letters.

The treatment arm of the present study (STAR) will receive 16 Gray SRT, as used in the INTREPID study. Both arms will receive ranibizumab *prn*, as used in the CATT trial. The primary outcome is the ranibizumab re-injection rate over 2 years. CATT reported a mean (±SD) of 6.9 ± 3.0 ranibizumab retreatments to the end of year 1 and 12.6 ± 6.6 to the end of year 2. The year 2 retreatment rate is most relevant to the STAR control group, which recruits patients with previously treated disease (CATT participants were treatment-naive at enrolment). The year 2 CATT retreatment was calculated to be 5.7 injections (12.6 – 6.9), so we might expect our control group to receive twice this (11.4) over two years. As CATT was undertaken in the US, to allow more conservative assumptions in case the injection rate is lower in the UK, we assume the injection rate to be 10 injections over 2 years in our control group, with a SD of 7 (based on INTREPID data which showed the SD was 69% of the mean). A 25% reduction in the number of injections is thought to be clinically and economically meaningful. Notwithstanding the fact that the second year of INTREPID was primarily designed to assess safety and not efficacy, this figure also matches the 25% reduction in the injection rate in the 2-year results of INTREPID, comparing the combined radiotherapy arms to the sham arm (Jackson et al, 2015).

1.7 Brief description of proposed analyses

Analyses will be carried out by the trial statistician. In the first instance data will be analysed under intention-to-treat assumptions (i.e. analyse all those with data in groups as randomised irrespective of treatment received).

2. Data analysis plan – Data description

2.1 Recruitment and representativeness of recruited patients

Recruitment, randomisation and follow-up for STAR will be summarised by arm in a CONSORT flow-diagram. This will include the main reasons for there being missing data (withdrawal, lost to follow up) by stages of the trial, and will also include the numbers for whom this occurs per arm. Also included will be the number randomised, who comprise the intention to treat trial population, and the numbers followed-up to be in the analyses of the primary outcome.

2.2 Baseline comparability of randomised groups

Baseline characteristics of each group will be summarised as mean and standard deviation for continuous variables with median and interquartile range for highly skewed data, and count and percentage for categorical variables. No significance testing on baseline variables will be performed.

The baseline characteristics will include patient demographics, randomisation stratifiers, ophthalmic history, medical history, EDTRS visual acuity, and other baseline (screening) clinical measures. This will allow an assessment of whether there is clinically important imbalance in any variables.

2.3 Loss to follow-up on outcome data

The proportions of participants with any missing data will be summarised by variable in each arm and at each time point. The baseline characteristics of those with missing primary outcome data will be compared statistically to those with complete follow up using appropriate univariate statistical tests. The reasons for withdrawal from the trial will be summarised in the CONSORT flow diagram.

Patients entering the trial have already become accustomed to one or two monthly hospital review and retention rates are expected to be high. In INTREPID loss to follow up at one year was 2.2%. CABERNET had 93% of data available for analysis at 2 years follow up. Our study size and power calculations allow for a 10% loss to follow up at the 2 year primary endpoint. To address any missingness that occurs, we will conduct a sensitivity analysis of the primary outcome that adjusted for any factors shown to be different between those present and those with full primary outcome data.

2.4 Adverse event reporting

AEs, adverse reactions (AR), SAEs, and serious adverse reactions (SAR) will be summarised as counts and percentages with 95% confidence intervals by trial arm. Where patients have not received the allocated treatment, this will be noted in reporting AEs so that the denominator for AEs is the number who actually received each treatment.

2.5 Descriptive statistics for outcome measures

The following outcomes will be reported at Month 24.

Primary Measure

- Number of as required (*prn*) ranibizumab injections during the first 24 months. This will be analysed as a continuous variable since while it is discrete, it is expected to have a wide range (1 to 20, as shown in the INTREPID study).

Secondary Measure

- Mean ETDRS VA.
- Percentage of participants losing < 15 ETDRS letters
- Percentage of participants gaining ≥ 0 ETDRS letters
- Percentage of participants gaining ≥ 15 ETDRS letters
- Total lesion size by fluorescein angiography
- Total CNV size by fluorescein angiography
- Foveal thickness measured using OCT
- Health-related quality of life assessed using the National Eye Institute 25- Item Visual Function Questionnaire and the EuroQol EQ-5D™ questionnaire
- Cost per Quality Adjusted Life Year (QALY)

Continuous outcome measures (per treatment arm) will be summarised as mean and standard deviation, with median and interquartile range where there is extreme skewness; categorical outcome measures as count and percentage. Also, mean vision change and mean OCT thickness will be plotted against time (24 monthly visits over two years) as summary measures showing vision change over time and OCT thickness over time to demonstrate the biological response to radiation.

3. Data analysis plan – Inferential analysis

3.1 Main analysis of treatment differences

The main statistical analyses will estimate the difference in mean outcome between patients randomised to SRT and sham by intention to treat at 24 months. Group difference estimates and associated 95% confidence intervals will be reported.

3.1.1 Analysis of primary outcomes

The principal analyses of primary outcome will be performed according to "intent-to-treat" principle. All randomized patients in these analyses will be classified according to their assigned treatment at randomization, regardless of patient's adherence. The primary analysis is to test the mean difference in number of ranibizumab retreatments up to and including Month 24 between the SRT and sham group (ranibizumab monotherapy). Previous research (CATT and INTREPID) has suggested that the number of injections is approximately normally distributed. In this case, a multiple linear regression analysis will be used to assess the treatment effect with adjustment for the baseline stratification factor – treatment centre. The analysis will not include the initial mandated ranibizumab treatment as it is administered to all participants, and

does not reflect the effect of SRT or sham treatment. The treatment effect is evaluated at the two-sided 0.05 significance level.

In the event that the number of injections is not normally distributed, a data transformation will be used to give normally distributed residuals. In the unlikely event that no transformation is possible, analysis will be based on a non- parametric approach, a stratified Wilcoxon-Mann-Whitney (WMW) test (the van Elteren test), adjusted for the baseline stratification factors and the median difference with 95% confidence interval calculated by the (stratified) Hodges-Lehmann estimation.

3.1.2 Analysis of secondary outcomes

The change in visual acuity (VA) will be formally tested statistically for non- inferiority. The change in VA in the SRT arm compared to the change in VA in the control arm from baseline to Month 24 will be analysed by using a multiple linear regression model with adjustment for the baseline stratification factor (treatment centre) and the baseline VA score. Multiple linear regression will be used rather than repeated measure analysis because although there will be 24 monthly visits for patients in the trial, the focus of interest is the mean changes in VA from baseline to Month 24.

Data from the other efficacy outcomes (listed in Section 1.5.2) will be summarized. Statistical analysis of these outcomes will be descriptive, with differences and 95% confidence intervals where possible. There will be no correction for multiple testing. Mean vision change and mean OCT thickness will be plotted against time (24 monthly visits over two years) as summary measures showing vision change over time and OCT thickness over time.

3.1.3 Planned subgroup analyses

Subgroup analyses of number of injections, mean VA and OCT thickness (as a forest plot) will be conducted for pre-specified subgroups defined by the following key variables. All subgroup effects will be tested by fitting an interaction factor in the model so that differences between subgroups will only be confirmed if the test for interaction is statistically significant. All tests will be at a statistically significance level of 5%.

1. Total angiographic lesion size, as per reading centre evaluation (above and below the median)
2. Greatest distance of the lesion from the foveal centre, as per the reading centre evaluation
3. Angiographic lesion type per reading centre:
    1. Type 1 (occult)
    2. Type 2 (classic)
    3. Type 3 (retinal angiomatous proliferation (RAP))
    4. Mixed (minimally classic)
    5. Idiopathic polypoidal choroidal vasculopathy (IPCV)
4. OCT macular volume per reading centre (above and below median)
5. Baseline vision in ETDRS letters (above and below median)
6. Duration of disease (above and below median)

7. Number of prior anti-VEGF injections excluding that given at baseline (above and below median)
8. Presence of absence of vitreomacular adhesion on OCT, as per reading centre.
9. Lens status (phakic or pseudophakic)

3.1.4 Statistical considerations

Missing outcome data

To address any missingness that occurs, we will conduct a sensitivity analysis of the primary outcome that adjusted for any factors shown to be different between those present and those with full primary outcome data.

Method for handling non-compliance

The number of patients who have not completed their full treatment protocol is expected to be few but will be noted. In addition to the primary intention-to-treat analysis the effect of actually receiving treatment as defined in the protocol will also be estimated by comparing the two arms in just those who have received the full protocol.

Method for handling non-conformity in randomisation

In the case that randomised treatment code is incorrectly applied by unforeseen reason, we will identify the patients potentially affected and establish which, if any, of those patients received the opposite treatment allocation to that randomised. Analyses will be based on intention-to-treat (ITT). A sensitivity analysis will be carried out using the 'actually received' treatment.

COVID-19 sensitivity analysis

The COVID-19 pandemic has affected research globally. For the UK-based STAR trial, national lockdown measures instituted in March 2020 have the potential to impact the primary outcome (number of ranibizumab injections up to year 2) if participants elected not to attend for review, or if hospitals changed their management pathway to mitigate the risks of patients contracting COVID.

To assess the impact of COVID-19, a sensitivity analysis of the primary outcome has been predefined, prior to data lock. This sensitivity analysis will be used to help interpret the main primary endpoint analysis, which remains intent-to-treat. The sensitivity analysis aims to assess how the pandemic might have altered the trial's primary outcomes.

The sensitivity analysis involves an evaluation of the primary outcome in four predefined sub-populations:

i. Participants whose year 2 primary endpoint was completed before the onset of the national lockdown on 23rd March 2020. These participants will have been largely or fully unaffected by the pandemic.

ii. Participants whose year 2 primary endpoint was completed after the onset of the national lockdown on 23rd March 2020. These participants will have been affected by the pandemic to varying degrees depending on when they enrolled and hoe many of their monthly visits occurred during the pandemic.

iii. Participants whose compliance up to their year 2 primary endpoint was sufficient.

iv. Participants whose compliance up to their year 2 primary endpoint may be compromised.

The distinction between group 3 and 4 is based on the application of predefined rules, which categorise visits as green (compliant), amber (some deviation but not one that is unlikely to materially affect the primary outcome), and red (deviations that may have affected the primary outcome). Participants will be considered to be sufficiently compliant with the protocol (group 3) if they had no more than 4 red visits or 8 amber visits up to their year 2 primary endpoint. The rules to categorise each visit are shown in appendix A.

Compliance will be shown graphically for the entire population, using a novel 'compliance' schematic. This will show the visit timeline for each participant up to the year 2 primary endpoint. Sequential visits will be shown in green, amber or red, as a horizontal row. The colour coded visit timeline for each participant will be stacked one above the other, from first to last participant recruited. Withdrawals will be shown in white, and deaths in black. Additionally, the onset of COVID restrictions will be marked on each participant's timeline (if it applies), so that the overall compliance, death rate, and withdrawals in the trial can be compared graphically before and after lockdown.

3.2 Exploratory analyses

Any examination of subgroups, not specifically identified in the protocol, will be considered exploratory in nature and will be clearly identified.

3.3 Interim analysis

The usual rationale for an interim analysis is to consider stopping the treatment (or the trial) however as this treatment is given at baseline, it is not possible to subsequently stop treatment. As such we elected not to include an interim analysis. The DMC will examine the recruitment rate, data completeness and monitor safety, and will recommend whether the study should continue, stop, be suspended, or be modified, based on their findings.

4. Software

Data management: An online data collection system for clinical trials (MACRO; InferMed Ltd) will be used. This is hosted on a dedicated server at King's College London and managed by King's CTU (KCTU). The KCTU Data Manager will extract data periodically as needed and provide these in comma separated (.csv) format.

Statistical analysis: Statistical software package R will be used for data description and the main inferential analysis.

B) SCHEDULE OF ASSESSMENTS AND MEASURES

Assessment	Screening	SRT with baseline ranibizumab †	Monthly review* (Month 1-11)	Month 12	Monthly review* (Month 13-23)	Month 24	Month 36	Month 48
Visit Window: *Day 0 = Day of successful enrolment*	Day -14 to 0	Day 0 to 21	±7 days	±7 days	±7 days	±7 days	±14 days	±14 days
Informed Consent	X							
Demographics	X							
Ophthalmic History	X							
Med History/Con Meds	X							
Blood Pressure	X							
EDTRS Visual Acuity	X		X	X	X	X	X	X
Intraocular Pressure	X			X		X	X	X
Cataract Assessment	X			X		X	X	X
Biometry	X							
OCT (sent to reading centre)	X			X		X	X	X
OCT (*not* sent to reading centre)	X		X		X			
Fundus Photographs (sent to reading centre)	X			X		X	X	X
Fluorescein Angiography (sent to reading centre)	X			X		X	X	X
Indocyanine Green Angiography (sent to reading centre)	X		X (?)					
Stereotactic Radiotherapy with mandated baseline Ranibizumab†		X						
Ranibizumab injection if required (*prn*)			X	X	X	X	X	X
EQ-5D and VFQ-25 patient questionnaires‡	X			X		X	X	X
AEs/ConMed changes			X	X	X	X	X	X

Appendix A: Compliance rules

Assessment Categorisation<br>Red, Amber, Green.	Visits (by month)					
	**1-11**	**12**	**13-23**	**24**	**36**	**48**
Visit attendance†<br>'Missed consultation'<br>As indicated by the status form (SF – not done [888/8888])<br><br>'Visit out of window'<br>For the purpose of this analysis, visits up to month 24 are in window if they are either within 14 days of the study visit planner target visit date, or from 21 to 42 days after the last visit. The greater tolerance for longer than shorter visits is because a shorter visit might reduce the likelihood of needing an injection, whereas a slightly longer visit will probably not, as macular fluid persists. For the two safety visits at month 36 and 48, the visit is in window if it occurs with 21 days of target, per the study scheduler, or from 49 to 57 weeks of the last visit.<br><br>'Visit within window'<br>The visit is in window if it meets one of the criterions listed immediately above.						
ETDRS VA<br>Not performed at visit – Visual acuity (Monthly or Annuals) = Q1 Visual acuity study eye = 888<br><br>- Visual acuity not performed but injection required at same visit regardless, due to other disease activity– i.e. Ranibizumab retreatment form states one of the following:<br>o Q2 Evidence of subretinal, intraretinal, or sub-RPE fluid on OCT? = 1 Yes<br>o Q3 New or persistent subretinal or intraretinal haemorrhage? = 1 Yes<br>o Q5 Increased lesion size on fluorescein angiography relative to last angiogram? = 1 Yes<br>o Q6 Leakage on fluorescein angiography? = 1 Yes<br>Performed per protocol	x	x*	x	x*	x*	x*

Fundus examination (posterior segment abnormality):<br>Not performed at visit – Fundal examination (Monthly) = 888<br><br>Not performed but any of the following performed (as they allow doctor to see fundus):<br>o OCT undertaken = Q2 Right central subfield thickness (µm) *or* Q7 Left central subfield thickness (µm) contains value – ie value that is not 7777 or 8888 or 9999.<br>o Fundus Photography = Q1 Performed on both eyes = 1 Yes<br>o Fluorescein Angiography (Annual) = Q1 Performed = 1Yes<br><br>Not performed but any of the following mandated treatment anyway (Ranibizumab retreatment form states one of the following):<br><br>o Q2 Evidence of subretinal, intraretinal, or sub-RPE fluid on OCT? = 1 Yes<br>o Q3 New or persistent subretinal or intraretinal haemorrhage? = 1 Yes<br>o Q5 Increased lesion size on fluorescein angiography relative to last angiogram? = 1 Yes<br>Q6 Leakage on fluorescein angiography? = 1 Yes<br><br>Performed per protocol	x	x	x	x	x	x
OCT<br>Not performed at visit – OCT = 8888<br><br>- OCT not performed but injection required anyway– i.e. Ranibizumab retreatment form states one of the following:<br>o Q3 New or persistent subretinal or intraretinal haemorrhage? = 1 Yes<br>o Q5 Increased lesion size on fluorescein angiography relative to last angiogram? = 1 Yes<br>o Q6 Leakage on fluorescein angiography? = 1 Yes<br>- OCT wrongly recorded as not performed (OCT 888) but actually was:<br>o Q2 Evidence of subretinal, intraretinal, or sub-RPE fluid on OCT? = 1 Yes<br><br>Performed per protocol	x		x			

Ranibizumab injection (prn)<br>Ranibizumab injection not given when indicated – i.e. Ranibizumab retreatment form indicates both of:<br>○ Q1 Does the patient require ranibizumab retreatment (regardless of whether injection actually given)? = 1 Yes<br>○ Q8 Ranibizumab 0.5µg administered? = 0 No<br><br>Given when indicated, but out of window by >14 days<br><br>Given when indicated, within 14-day window	x	x	x	x	x	x

†If a visit was missed but determined to be amber because no injection was needed at the next visit, ignore the fact that VA, OCT and fundus examination were 'missing' during the non-attended visit, even if their absence would otherwise define a red visit.

Typical codes on MACRO:
888/8888 = not done 999/9999 = data unknown
777/7777 = not available or not applicable
1 = Yes
0 = No

Amendments to version 1.0

Version 1.1 (29 July 2015) includes following amendments:

3.1.4 Statistical considerations

Method for handling non-conformity in randomisation

In the case that randomised treatment code is incorrectly inputted by human error, we will identify the patients potentially affected and establish which, if any, of those patients received the opposite treatment allocation to that randomised. Analyses will be based on treatment actually received and any deviation from the randomisation will be documented. A sensitivity analysis will be carried out excluding those affected patients.

Amendments to version 1.1

Version 1.2 (19 August 2015) includes the following change of wording in the method for handling non-conformity in randomisation (under 3.1.4 Statistical considerations): ‘human error’ is changed to ‘unforeseen reason’.

Amendments to version 1.2

Version 1.3 (06 November 2015) includes following amendments in the planned subgroup analyses (section 3.1.3) regarding names of angiographic lesion subtypes:

Angiographic lesion type per reading centre:

1. Type 1 (occult)
2. Type 2 (classic)
3. Type 3 (retina angiomatous proliferation (RAP))
4. Mixed (minimally classic)
5. Idiopathic polypoidal choroidal vasculopathy (IPCV)

Amendments to version 1.3

Version 1.4 (14 April 2022) includes the following amendments:

- Addition of “COVID-19 sensitivity analysis” section under “3.1.4 Statistical considerations”.
- Addition of appendix A defining visit compliance rules for the planned COVID-19 sensitivity analysis.
- Removal of section B “Economic Analysis Plan” (a separate document detailing the plan for health economic analysis will be provided).

Reference List

1. Protocol for StereoTactic radiotherapy for wet Age-Related macular degeneration (STAR): A randomised, double-masked, sham-controlled, clinical trial comparing low-voltage X-ray irradiation with as needed ranibizumab, to as needed ranibizumab monotherapy. STAR trial protocol version 1.3. Date: 25 Oct 2014.

2. CATT Group, Martin DF, Maguire MG, et al. Ranibizumab and bevacizumab for neovascular age-related macular degeneration. N Engl J Med 2011;364(20):1897-908.

3. Jackson TL, Dugel PU, Bebchuk JD, et al. Epimacular Brachytherapy for Neovascular Age-Related Macular Degeneration (CABERNET): Fluorescein Angiography and Optical Coherence Tomography. Ophthalmology 2013.

4. Jackson TL, Chakravarthy U, Kaiser PK, et al. Stereotactic Radiotherapy for Neovascular Age-Related Macular Degeneration: 52-Week Safety and Efficacy Results of the INTREPID Study. Ophthalmology 2013.

5. Investigators IS, Chakravarthy U, Harding SP, et al. Ranibizumab versus bevacizumab to treat neovascular age-related macular degeneration: one- year findings from the IVAN randomized trial. Ophthalmology 2012;119(7):1399-411.

6. Moher D, Schulz KF, Altman DG. The CONSORT statement: revised recommendations for improving the quality of reports of parallel-group randomised trials. Lancet 2001 Apr 14;357(9263):1191-4.

7. Moher D, Hopewell S, Schulz KF, Montori V, Gotzsche PC, Devereaux PJ, Elbourne D, Egger M, Altman DG. Consort 2010 explanation and elaboration: Updated guidelines for reporting parallel group randomised trials. *Bmj*. 2010;340:c869

8. White IR, Thompson SG. Adjusting for partially missing baseline measurements in randomized trials. Stat Med 2005 Apr 15;24(7):993-1007.

9. White IR, Horton NJ, Carpenter J, Pocock SJ. (2011) Strategy for intention to treat analysis in randomised trials with missing outcome data. BMJ 342:d40

10. Lehmann EL. Nonparametrics: Statistical methods based on ranks. 1975. San Francisco: Holden-Day. 457 pp.

4. ***Post-hoc* analysis plan**

Additional *post-hoc* analyses were undertaken to investigate the effect of switching anti-VEGF agent after week 96 on the primary outcome (number of injections) and secondary outcome (mean ETDRS BCVA and macular thickness) at weeks 144 and 192 specifically. This has been reported in appendix figures S5-10, pages 50-55 of this appendix as an add-on to the other pre-specific subgroup analyses.

Additionally, *post-hoc* analyses were undertaken to investigate if the reason for stable VFQ-25 throughout the trial may have been due to the worse-seeing eye chosen as the study eye at baseline and, in light of the unexpected drop in ETDRS BCVA in SRT-treated eyes at weeks 144 and 192, to investigate if a main contributory cause could be determined. The potential contributors, reasons for their effect on vision, and method of analysis are described in table S1, and are represented in tables S17-23, pages 56-59 and figures S11 and S12, pages 60-61 of this appendix.

5. **Table S1: Explanatory causes of vision loss**

Explanatory cause	Mechanism of vision loss	Method of post-hoc analysis
Follow up during COVID lockdown	Participants may have been under-treated due to lockdown rules limiting clinic appointments. Equally, ETDRS BCVA assessments may not have been as thorough or may have been modified due to pressures limit in-clinic time.	Mean ETDRS BCVA in SRT and sham SRT eyes disaggregated by participants who attended week 144 and 192 visits before and after COVID-lockdown.
Lens opacity	Participants had lens opacities graded by clinicians, and it is possible participants may have had visually significant lens opacity that was not reported as cataract by investigators.	Mean ETDRS BCVA in SRT and sham SRT eyes disaggregated by participants with visually significant lens opacities, defined as having either (1) a cataract reported as an adverse event (and untreated) or (2) a phakic lens grading of nuclear sclerosis OR cortical OR posterior subcapsular cataract of ≥2.0 per AREDS 2008 clinical lens opacity photographs at weeks 144 and 192.
Subfoveal fibrosis<br>Subfoveal atrophy<br>Subfoveal ellipsoid zone disruption	Participants with reading centre-graded subfoveal fibrosis, atrophy or ellipsoid zone disruption at weeks 144 and 192 would be most likely to manifest vision loss due to permanent photoreceptor injury.	Mean ETDRS BCVA in SRT and sham SRT eyes disaggregated by presence (yes/no) of subfoveal fibrosis, atrophy or ellipsoid zone disruption.
Total lesion area<br>Total active lesion area	Participants with greater reading centre-graded total lesion area and total active lesion area at weeks 144 and 192 could manifest greater vision loss due to active nAMD disease.	Mean ETDRS BCVA in SRT and sham SRT eyes disaggregated by median total lesion and area and total active lesion area (below / above median).
Concomitant macular pathology	Participants with reading centre-graded macular co-pathology that was not otherwise reported as adverse events by investigators at weeks 144 and 192 could manifest greater vision loss due to concurrent disease.	Mean ETDRS BCVA in SRT and sham SRT eyes disaggregated by presence of macular co-pathology (yes/no).

Table S1: Explanatory causes for vision loss in study eyes. Abbreviations: anti-VEGF, anti-vascular endothelial growth factor; AREDS, age-related eye disease study lens opacity classification criteria; BCVA, best-corrected visual acuity; COVID, covid-19 lockdown; ETDRS, early treatment of diabetic retinopathy study letters; nAMD, neovascular age-related macular degeneration; SRT, stereotactic radiotherapy.

6. **Additional methods of costing analyses**

Statistical analysis of cost data

For the costing analyses, we compared the cost of nAMD treatment between the SRT and sham groups over a 192-week time horizon. This included costs for SRT and the first ranibizumab dose, anti-VEGF and administration, and monitoring consultations.

SRT and First Ranibizumab Dose

We used the micro-costing estimate of the cost of SRT that has been published previously, which includes the manufacturer's license fee and staff costs involved.[1] No cost was applied to sham SRT. Ranibizumab was costed as Lucentis (not a biosimilar) using the list price in the economic evaluation, as there is no publicly available NHS pricing data.

Anti-VEGF and administration

Between weeks 1 and 96, ranibizumab was administered whenever patients had active disease, defined by trial re-treatment criteria,[2] with participants attending reviews every four weeks. Between weeks 97 and 192, participants returned to routine care and could receive different anti-VEGF drugs, monitoring schedules and retreatment criteria. We cost injections according to the named drug in each participant's log. If no drug was specified, we imputed an estimated cost using the weighted average cost of all anti-VEGF injections given in that year of the trial.

Monitoring Consultations

Between weeks 1 and 96, participants returned to their recruiting centre every four weeks. No cost was applied to visits that were scheduled but unattended, regardless of the reason, including the COVID-19 pandemic or lockdowns.

Between weeks 97 and 192, participants returned to routine care with varying follow-up intervals. Clinicians classified each participant into one of the three monitoring regimens that were in use at the time, based on health economic utilization forms and injection logs, independent of randomisation:

- Treat-and-Extend (T&E): Participants had an injection every visit, with the visit duration modified based on disease activity, with a maximum interval of 16 weeks. Anti-VEGFs monitored under this regimen included aflibercept (Eylea), faricimab (Vabysmo), ranibizumab (Lucentis), and bevacizumab (Avastin).[3]
- Pro Re Nata (PRN): The monitoring visit interval was fixed at 4 weeks, with injections depending on disease activity. Anti-VEGFs monitored under this regimen included ranibizumab (Lucentis) and bevacizumab (Avastin). [3]
- Observation: Stable disease status with no treatment. If a participant had no injections for ≥16 weeks between weeks 97 to 192, they were classified under observation from the last injection until injections resumed or the participant reached a follow-up endpoint.

Participants could switch regimens during the study. Injection dates for both study and non-study eyes determined each participant's monitoring regimen. The cost of monitoring AMD with OCT was estimated based on the duration of participant time spent on each regimen, calculated from the actual 2-year visit date up to the planned 3-year and 4-year dates, separately:

- T&E: One OCT cost per injection.
- PRN: One OCT cost per 28 days: we applied 0.035714286 (=1/28) OCTs per day on this regimen.
- Observation: One OCT cost every 16 weeks: we applied 0.010951403 (=4/365.25) OCTs per day on this regimen.

Total Cost of SRT, Anti-VEGF, and Monitoring

Unit costs were taken from Adapted from Jackson et al. (2024) Table S5.[1] The reference year for costs was 2021-22. [4-6] VAT was excluded per NICE manual guidelines.[7]

We summed the costs of treating nAMD over a 192-week horizon, discounting costs more than one year after randomization at 3.5% per annum. To minimize bias from chance imbalances between randomized groups, we used linear regression to adjust for age, gender, baseline EQ-5D utility, baseline EDTRS visual acuity, and days affected by COVID-19. Adjusting for age and gender primarily accounted for potential differences in mortality due to chance imbalances. Adjusting for baseline EQ-5D utility accounted for differences in participants' initial health status.[9] Adjusting for the timing of COVID-19 restrictions helped address any chance of imbalances in the number of follow-up appointments and injections cancelled due to the pandemic. Days affected by COVID-19 were controlled for during two periods: 1-2 years (from the start of the first lockdown to the second anniversary of

randomization) and 3-4 years (from the second anniversary to the date when England reverted to Plan A, stopping the requirement for face coverings and COVID-19 Passes).[10] To quantify uncertainty, we bootstrapped the cost estimates 1,000 times and ran each regression on each bootstrap. We then calculated 95% confidence intervals using the 2.5th and 97.5th percentiles from the 1,000 bootstraps.

Since SRT only applied to the study eye, data were not collected on treatments or consultations related to the fellow eye and all monitoring visits and ranibizumab injections were costed on the basis that only the study eye was being treated. Analyses on the cost of SRT and first ranibizumab dose were not adjusted for covariates, as variability arose only from non-attendance or receiving opposite treatments from randomization allocation.

7. **Table S2: STAR study health economic Unit costs**

Resource (units)	Cost (£)	Source
Monitoring consultation	159.05	Outpatient procedure code for retinal tomography (ophthalmology) 19 years and over BZ88A in National Schedule of NHS Costs Year 2021-22 Outpatient procedures.[4]
Injection consultation	157.39	A weighted average of two procedure codes (weighting by number of examinations in England): BZ86B Intermediate Vitreous Retinal Procedures, 19 years and over, with CC Score 0-1 (Ophthalmology) and BZ87A Minor Vitreous Retinal Procedures, 19 years and over (Ophthalmology). National Schedule of NHS Costs Year 2021-22 Outpatient procedures.[4]
Ranibizumab	551.00	British National Formulary.[3] Cost per vial or pre-filled disposable injection containing ranibizumab (Lucentis) 10 mg/mL solution for injection.
Faricimab	857.00	British National Formulary.[3] Cost per pre-filled syringes containing Faricimab (Vabysmo) 120mg/ml solution for injection.
Bevacizumab	58.41	Micro-costing from the IVAN study,[11] also used in recent NICE appraisals,[12,13] was based on manual repackaging and dilution of bevacizumab (Avastin) 25 mg/mL solution. Medicine cost was from the British National Formulary,[3] with 28% attributed to ingredients. Non-medicine costs were inflated using the Personal Social Services Pay & Prices Index. [4]
Aflibercept	£816.00	British National Formulary.[3] Cost per vial or pre-filled disposable injection containing Aflibercept (Eylea) 40 mg/mL solution for injection.
SRT	1342.91	Micro-costing from the STAR study. [1]

Table S2: Adapted from Jackson et al. (2024) Table S5.[1] Abbreviations: anti-VEGF, anti-vascular endothelial growth factor; nAMD, neovascular age-related macular degeneration; SRT, stereotactic radiotherapy.

8. **Costing analysis: Results**

Over the 192-week follow-up period, the SRT group had lower costs for anti-VEGF and administration (£13819.28 vs. £15093.43; p=0.15): a difference of -£1274.15 (95% CI: -£3245.95 to £580.49). Monitoring consultation costs were similar between treatment groups (p=0.63). In the analysis population used for 4 year anti-VEGF injections and BCVA, the total treatment costs were £61.65 (95% CI: -£2220.51 to £1979.54) lower in the SRT group (p=0.95). A full economic evaluation with multiple imputation of missing data and a more comprehensive range of costs will be reported separately.

9. **Table S3: Mean cost of neovascular age-related macular degeneration treatment (UK 2021-22 pounds)**

Costs‡	SRT (n=222)	Sham SRT (n=106)	Difference (SRT-sham SRT)
SRT and first ranibizumab dose	1893.91	730.76	1163.15
Anti-VEGF and administration	13819.28 (12361.58, 15214.13)	15093.43 (13566.62, 17045.94)	-1274.15 (-3245.95, 580.49)

Monitoring consultations	5466.01 (5267.98, 5654.34)	5416.67 (5234.26, 5629.35)	49.34 (-170.07, 263.58)
Total costs of SRT, anti-VEGF and monitoring	21179.20 (19563.39, 22726.32)	21240.85 (19564.06, 23374.58)	-61.65 (-2220.51, 1979.54)

Table S3: Mean costs (95% confidence interval) from week 1 to 192 based on a complete case intention-to-treat analysis excluding patients who died, withdrew, or were lost to follow up. ‡ Costs were discounted at 3.5% following the UK's National Institute for Health and Care Excellence guidelines and were adjusted for age, gender, baseline EQ-5D utility, baseline best-corrected visual acuity, and days affected by COVID-19. Mean values were presented for women aged 78 years, who were unaffected by the COVID pandemic, with a visual acuity of 69 letters and an EQ-5D-5L utility score of 0.84. Abbreviations: SRT, stereotactic radiotherapy; VEGF, vascular endothelial growth factor.

10. **Table S4: Number of participants selecting each anti-VEGF drug in years 3 and 4 (available cases)**

	**Standard care**	**SRT plus anti-VEGF**	**Total**
Ranibizumab	86	175	261
Bevacizumab	0	1	1
Aflibercept	8	23	31
Faricimab	0	1	2

Table S4: This table was based on the anti-VEGF injection log for the study eye reported by hospital records. Participants could switch between drugs in years three and four. Abbreviations: VEGF, vascular endothelial growth factor; SRT, stereotactic radiotherapy.

11. **Costing analysis:** References

1. Jackson TL, Desai R, Wafa HA, et al. Stereotactic radiotherapy for neovascular age-related macular degeneration (STAR): a pivotal, randomised, double-masked, sham-controlled device trial. *Lancet* 2024.
2. CATT Research Group, Martin DF, Maguire MG, et al. Ranibizumab and bevacizumab for neovascular age-related macular degeneration. *New England Journal of Medicine* 2011; **364**(20): 1897-908.
3. National Institute for Health and Care Excellence. British National Formulary (BNF). 13 December 2023. https://bnf.nice.org.uk/ (accessed 29 Feburary 2024.
4. Jones K, H. W. Unit Costs of Health and Social Care 2022. 2022. https://kar.kent.ac.uk/id/eprint/100519 (accessed 22 December 2023
5. Joint Formulary Committee. British National Formulary. London: BMJ and Pharmaceutical Press; March 2022.
6. NHS Digital. National Cost Collection: National Schedule of NHS costs - Year 2021-22 - NHS trust and NHS foundation trusts. 2023. https://www.england.nhs.uk/costing-in-the-nhs/national-cost-collection/ (accessed 3 March 2023.
7. National Institute for Health and Care Excellence. NICE health technology evaluations: the manual. 31 January 2022. https://www.nice.org.uk/process/pmg36/resources/nice-health-technology-evaluations-the-manual-pdf-72286779244741 (accessed 7 February 2022.
8. DB R. Multiple imputation for nonresponse in surveys. New York: Wiley; 1987.
9. Manca A, Hawkins N, Sculpher MJ. Estimating mean QALYs in trial-based cost-effectiveness analysis: the importance of controlling for baseline utility. *Health Econ* 2005; **14**(5): 487-96.
10. Department of Health and Social Care, The Rt Hon Sajid Javid MP. England returns to Plan A as regulations on face coverings and COVID Passes change today. 2022. https://www.gov.uk/government/news/england-returns-to-plan-a-as-regulations-on-face-coverings-and-covid-passes-change-today (accessed 17 June 2024.
11. IVAN Study Investigators, Chakravarthy U, Harding SP, et al. Ranibizumab versus bevacizumab to treat neovascular age-related macular degeneration: one-year findings from the IVAN randomized trial. *Ophthalmology* 2012; **119**(7): 1399-411.
12. National Institute for Health and Care Excellence. Brolucizumab for treating wet age-related macular degeneration [ID1254] Committee Papers. 2020. https://www.nice.org.uk/guidance/ta672/evidence/committee-papers-pdf-8964536941 (accessed 11 May 2022.
13. National Institute for Health and Care Excellence. Age-related macular degeneration NICE guideline [NG82] Appendix J: Health economics. 2018. https://www.nice.org.uk/guidance/ng82/evidence/appendix-j-health-economics-pdf-170036251093.

Missingness and adherence: Missingness

12. **Table S5: Missingness in baseline and outcome variables.**

	SRT (N=274)	Sham SRT (N=137)
**Demographic characteristics**		
Age	-	-
Female	-	-
Ethnicity	-	-
Smoking status	-	-
**Ophthalmic history**		
nAMD duration	5 (2%)	6 (4%)
Number of previous anti-VEGF injections	1 (<1%)	7 (5%)
Lens status	-	-
ETDRS visual acuity	-	-
Total lesion size	14 (5%)	7 (5%)
Total active lesion size	22 (8%)	8 (6%)
Central subfield thickness	1 (<1%)	-
Total macular volume	4 (1%)	4 (3%)
**Patient-reported outcome measures at baseline**		
NEI VFQ-25 composite score	7 (3%)	-
EQ-5D (VAS)	7 (3%)	1 (<1%)
**Outcomes at Week 48**		
Number of *prn* anti-VEGF injections	15 (5%)	10 (7%)
ETDRS visual acuity	17 (6%)	11 (8%)
Total lesion size	48 (18%)	30 (22%)
Total active lesion size*	101 (37%)	44 (32%)
Central subfield thickness	14 (5%)	11 (8%)
NEI VFQ-25 composite score	19 (7%)	15 (11%)
EQ-5D (VAS)	24 (9%)	15 (11%)
**Outcomes at Week 96**		
Number of *prn* anti-VEGF injections	33 (12%)	19 (14%)
ETDRS visual acuity	35 (13%)	17 (12%)
Total lesion size	67 (24%)	35 (26%)
Total active lesion size*	138 (50%)	54 (39%)
Central subfield thickness	27 (10%)	19 (14%)
NEI VFQ-25 composite score	33 (12%)	18 (13%)
EQ-5D (VAS)	33 (12%)	18 (13%)
**Outcomes at Week 144**		
Number of *prn* anti-VEGF injections	48 (18%)	29 (21%)
ETDRS visual acuity	46 (17%)	30 (22%)
Total lesion size	70 (26%)	41 (30%)
Total active lesion size*	144 (53%)	69 (50%)
Central subfield thickness	41 (15%)	25 (18%)
NEI VFQ-25 composite score	43 (16%)	23 (17%)
EQ-5D (VAS)	47 (17%)	23 (17%)
**Outcomes at Week 192**		
Number of *prn* anti-VEGF injections	59 (22%)	33 (24%)
ETDRS visual acuity	49 (18%)	34 (25%)
Total lesion size	80 (29%)	45 (33%)
Total active lesion size*	173 (63%)	80 (58%)
Central subfield thickness	51 (19%)	34 (25%)
NEI VFQ-25 composite score	50 (18%)	31 (23%)
EQ-5D (VAS)	55 (20%)	32 (23%)

*Table S5: Data are count (%). *High proportions of missingness in "Total active lesion size" were due to inactive lesions (graded as "NULL") being interpreted as "missing"; this is therefore not truly missing data. Abbreviations: anti-VEGF = anti-Vascular Endothelial Growth Factor; EQ-5D-5L (VAS), Euroqol questionnaire with visual analogue scale; ETDRS, Early Treatment Diabetic Retinopathy Study; nAMD, neovascular age-related macular degeneration; NEI VFQ-25, National Eye Institute 25-item visual function questionnaire; p.r.n. = pro-re-nata dosing regimen; SRT = stereotactic radiotherapy.*

13. **Table S6: Reasons for missingness**

	SRT (N=274)	Sham SRT (N=137)	Overall (N=411)
**Reason for missingness at Week 192**			
Adverse event	1 (<1%)	-	1 (<1%)
Participant intercurrent illness	4 (1%)	4 (3%)	8 (2%)
Participant withdrew consent	4 (1%)	4 (3%)	8 (2%)
No longer able to travel to centre	9 (3%)	4 (3%)	13 (3%)
Unable to locate / contact participant	1 (<1%)	1 (<1%)	2 (<1%)
Death of participant	17 (6%)	5 (4%)	22 (5%)
Other/Unknown*	16 (6%)	13 (9%)	29 (7%)

*Table S6: Reasons for missingness are described. *Examples of "other" included site-level technical failures (such as unforeseen equipment failure or staff unavailability meaning assessments could not be completed), site-level Covid-related changes to practice limiting tests that could be performed, and participants who opted not to attend but had not withdrawn consent.*

Missingness and adherence: Adherence

14. **Table S7: Classification of visit adherence**

Assessment Categorisation

Red (poor adherence), Amber (reduced adherence), Green (per protocol)

Visit attendance

Red = 'Missed consultation'

Amber = 'Visit out of window'
For the purpose of this analysis, visits up to week 96 are in window if they are either within 14 days of the study visit planner target visit date, or from 21 to 42 days after the last visit. The greater tolerance for longer than shorter visits is because a shorter visit might reduce the likelihood of needing an injection, whereas a slightly longer visit will probably not, as macular fluid persists.

Green = 'Visit within window'

Best-correct visual acuity

Red = Not performed at visit and does not meet criteria for amber

Amber = Visual acuity not performed but injection required at same visit regardless, due to other disease activity (for example, fluid on OCT such that visual acuity would not alter decision to inject)

Green = Performed as per protocol

Fundus examination

Red = Not performed at visit and does not meet criteria for amber

Amber = Not performed but any of the following performed (as they allow doctor to see fundus):
- OCT
- Fundus Photography
- Fluorescein Angiography

Not performed but any of the following mandated treatment anyway:
- Evidence of subretinal, intraretinal, or sub-RPE fluid on OCT
- New or persistent subretinal or intraretinal haemorrhage
- Increased lesion size on fluorescein angiography relative to last angiogram
- Leakage on fluorescein angiography

Green = Performed as per protocol

Optical coherence tomography

Red = Not performed at visit and does not meet criteria for amber

Amber = OCT not performed but injection required anyway:

- New or persistent subretinal or intraretinal haemorrhage
- Increased lesion size on fluorescein angiography relative to last angiogram
- Leakage on fluorescein angiography

OCT wrongly recorded as not performed but actually was, as investigator records evidence of subretinal, intraretinal, or sub-RPE fluid on OCT

Green = Performed as per protocol

Ranibizumab injection

Red = Ranibizumab injection not given when indicated

Amber = Given when indicated, but out of window by >14 days

Green = Given when indicated, within 14-day window

15. **Table S8: Outcomes of EQ-5D-5L domains by treatment groups at different visit weeks**

	SRT	Sham SRT
Any mobility problems		
Week 48	91 (36%)	43 (35%)
Week 96	89 (37%)	56 (47%)
Week 144	82 (36%)	56 (49%)
Week 192	92 (41%)	54 (51%)
Any self-care problems		
Week 48	25 (10%)	13 (11%)
Week 96	25 (10%)	15 (12%)
Week 144	24 (11%)	18 (16%)
Week 192	31 (14%)	17 (16%)
Any usual activities problems		
Week 48	83 (33%)	45 (38%)
Week 96	93 (39%)	46 (38%)
Week 144	92 (40%)	52 (46%)
Week 192	93 (42%)	51 (49%)
Any pain/discomfort problems		
Week 48	114 (45%)	52 (43%)
Week 96	110 (45%)	52 (43%)
Week 144	105 (46%)	54 (47%)
Week 192	100 (45%)	50 (48%)
Any anxiety/depression problems		
Week 48	51 (20%)	31 (25%)
Week 96	51 (21%)	31 (26%)
Week 144	60 (26%)	35 (31%)
Week 192	72 (33%)	34 (32%)
Any vision problems		
Week 48	144 (57%)	77 (63%)
Week 96	141 (58%)	65 (54%)
Week 144	147 (64%)	71 (62%)
Week 192	145 (66%)	74 (70%)

Table S8: Data are n (%). Abbreviations: SRT, stereotactic radiotherapy.

16. **Table S9: Sensitivity analyses of missing data and COVID pandemic impact: primary outcome at Week 48 (year 1)**

	Mean number of injections		Difference (95% confidence interval)	P-Value
	SRT	Sham SRT		
Base Intention-To-Treat (ITT) model	(N=259)	(N=127)		
	6	7·1	-1·1 (-1·8 to -0·4)	0·001*
(i) All assessments up to primary outcome completed before COVID lockdown [a]	(N=221)	(N=106)		
	6	7·2	-1·2 (-1·9 to -0·4)	0·002*
(ii) At least one assessment up to primary outcome after COVID lockdown [b]	(N=38)	(N=21)		
	6·2	6·5	-0·3 (-2·2 to 1·6)	0·74
(iii) Fully adherent population model [c]	(N=255)	(N=123)		
	6	7·2	-1·1 (-1·8 to -0·5)	0·0009*
(iv) Reduced adherence population model [d]	(N=4)	(N=4)		
	5·1	5	N/A	N/A
Base ITT model with additional adjustments [e]	(N=259)	(N=127)		
	5·9	7·1	-1·1 (-1·8 to -0·5)	0·0009*
Per-Protocol model [f]	(N=261)	(N=125)		
	6	7·1	-1·1 (-1·8 to -0·4)	0·001*
Base ITT model with multiple imputation [g]	(N=274)	(N=137)		
	6·1	7·1	-1 (-1·63 to -0·4)	0·002*

*Table S9: All models are adjusted for baseline stratification factor – National Treatment Centre. The mean in stereotactic radiotherapy (SRT) group is calculated by adding the obtained coefficients to the mean number of injections in the sham SRT group (i.e., adjusted means). *Denotes significant test ($p < 0·05$).*

a. *Main ITT model restricted to participants who completed all 12 visits (4-weekly) before the COVID lockdown 23 March 2020 (population (i) in the manuscript methods section).*
b. *ITT model restricted to participants who had at least one visit due after lockdown (population (ii) in the manuscript methods section).*
c. *ITT model restricted to the more adherent population defined as participants who had no more than four non-adherent (red) visits or eight less adherent (amber) visits, and who did not die or withdraw (population (iii) in the manuscript methods section). Definitions of red and amber visits are given in Statistical analysis plan Appendix A; and Table S7, appendix p35-36.*
d. *ITT model restricted to the less adherent population (population (iv) in the manuscript methods section). In this model, there was insufficient data to estimate difference in mean number of injections.*
e. *Main intention-to-treat (ITT) model with additional adjustment for variables showing significant difference ($p < 0·05$) between participants with complete primary outcome and those without.*
f. *Groups categorized according to the received treatment (i.e., per-protocol analysis) irrespective of adherence.*
g. *ITT model on multiply imputed dataset. Multiple imputation with chained equations was applied to generate 20 datasets. Baseline demography, ophthalmic history, and recruitment site were used to impute the 25 incomplete values in the outcome variable. Parameter estimates were finally combined using Rubin's principles.*

Base ITT model was undertaken as per statistical analysis plan for primary outcome. Models (i) – (iv) are pre-specified and labelled in the protocol and statistical analysis plan. All other models are post-hoc.

Abbreviations: anti-VEGF, anti-Vascular Endothelial Growth Factor; ITT, intention-to-treat model; SRT, stereotactic radiotherapy.

17. **Table S10: Sensitivity analyses of missing data and COVID pandemic impact: primary outcome at Week 96 (year 2)**

		Mean number of injections		Difference (95% confidence interval)	P-Value
		SRT	Sham SRT		
Base Intention-To-Treat (ITT) model		(N=241)	(N=118)		
		10·3	13·3	-2·9 (-4·2 to -1·6)	<0·0001*
(i)	All assessments up to primary outcome completed before COVID lockdown [a]	(N=152)	(N=73)		
		10·7	13·7	-3 (-4·6 to -1·4)	0·0004*
(ii)	At least one assessment up to primary outcome after COVID lockdown [b]	(N=89)	(N=45)		
		9·4	12·5	-3·1 (-5·3 to -0·8)	0·008*
(iii)	Fully *adherent* population model [c]	(N=225)	(N=100)		
		10·4	13·5	-3·1 (-4·5 to -1·7)	<0·0001*
(iv)	Reduced adherence population model [d]	(N=16)	(N=18)		
		10·4	11·8	-1·4 (-5·2 to 2·5)	0·47
Base ITT model with additional adjustments [e]		(N=241)	(N=118)		
		10·3	13·3	-3 (-4·3 to -1·7)	<0·0001*
Per-Protocol model [f]		(N=243)	(N=116)		
		10·3	13·2	-2·9 (-4·2 to -1·6)	<0·0001*
Base ITT model with multiple imputation [g]		(N=274)	(N=137)		
		10·8	13·3	-2·4 (-3·6 to -1·2)	<0·0001*

*Table S10: All models are adjusted for baseline stratification factor – National Treatment Centre. The mean in stereotactic radiotherapy (SRT) group is calculated by adding the obtained coefficients to the mean number of injections in the sham SRT group (i.e., adjusted means). *Denotes significant test (p < 0·05).*

h. Main ITT model restricted to participants who completed all 24 visits (4-weekly) before the COVID lockdown 23 March 2020 (population (i) in the manuscript methods section).
i. ITT model restricted to participants who had at least one visit due after lockdown (population (ii) in the manuscript methods section).
j. ITT model restricted to the more adherent population defined as participants who had no more than four non-adherent (red) visits or eight less adherent (amber) visits, and who did not die or withdraw (population (iii) in the manuscript methods section). Definitions of red and amber visits are given in Statistical analysis plan Appendix A; and Table S7, appendix p35-36.
k. ITT model restricted to the less adherent population (population (iv) in the manuscript methods section).
l. Main intention-to-treat (ITT) model with additional adjustment for variables showing significant difference (p < 0·05) between participants with complete primary outcome and those without.
m. Groups categorized according to the received treatment (i.e., per-protocol analysis) irrespective of adherence.
n. ITT model on multiply imputed dataset. Multiple imputation with chained equations was applied to generate 20 datasets. Baseline demography, ophthalmic history, and recruitment site were used to impute the 52 incomplete values in the outcome variable. Parameter estimates were finally combined using Rubin's principles.

Base ITT model was undertaken as per statistical analysis plan for primary outcome. Models (i) – (iv) are pre-specified and labelled in the protocol and statistical analysis plan. All other models are post-hoc.

Abbreviations: anti-VEGF, anti-Vascular Endothelial Growth Factor; ITT, intention-to-treat model; SRT, stereotactic radiotherapy.

18. **Table S11: Sensitivity analyses of missing data and COVID pandemic impact: primary outcome at Week 144 (year 3)**

		Mean number of injections		Difference (95% confidence interval)	P-Value
		SRT	Sham SRT		
Base Intention-To-Treat (ITT) model		(N=226)	(N=108)		
		14·6	17·7	-3·1 (-5 to -1·2)	0·002*
(i)	All assessments up to primary outcome completed before COVID lockdown [a]	(N=85)	(N=41)		
		16·8	18·6	-1·8 (-4·9 to 1·3)	0·25
(ii)	At least one assessment up to primary outcome after COVID lockdown [b]	(N=141)	(N=67)		
		13·1	17·2	-4 (-6·6 to -1·5)	0·002*
(iii)	Fully *adherent* population model [c]	(N=205)	(N=90)		
		14·7	17·8	-3·1 (-5·2 to -1)	0·004*
(iv)	Reduced adherence population model [d]	(N=21)	(N=18)		
		17·6	17·3	0·3 (-6·1 to 6·7)	0·92
Base ITT model with additional adjustments [e]		(N=226)	(N=108)		
		14·6	17·7	-3·1 (-5 to -1·2)	0·002*
Per-Protocol model [f]		(N=228)	(N=106)		
		14·6	17·6	-3·1 (-5 to -1·2)	0·002*
Base ITT model with multiple imputation [g]		(N=274)	(N=137)		
		15	17·7	-2·7 (-4·4 to -0·9)	0·003*

*Table S11: All models are adjusted for baseline stratification factor – National Treatment Centre. The mean in stereotactic radiotherapy (SRT) group is calculated by adding the obtained coefficients to the mean number of injections in the sham SRT group (i.e., adjusted means). *Denotes significant test (p < 0·05).*

o. Main ITT model restricted to participants who completed all 24 visits (4-weekly) before the COVID lockdown 23 March 2020 (population (i) in the manuscript methods section).
p. ITT model restricted to participants who had at least one visit due after lockdown (population (ii) in the manuscript methods section).
q. ITT model restricted to the more adherent population defined as participants who had no more than four non-adherent (red) visits or eight less adherent (amber) visits, and who did not die or withdraw (population (iii) in the manuscript methods section). Definitions of red and amber visits are given in Statistical analysis plan Appendix A; and Table S7, appendix p35-36.
r. ITT model restricted to the less adherent population (population (iv) in the manuscript methods section).
s. Main intention-to-treat (ITT) model with additional adjustment for variables showing significant difference (p < 0·05) between participants with complete primary outcome and those without.
t. Groups categorized according to the received treatment (i.e., per-protocol analysis) irrespective of adherence.
u. ITT model on multiply imputed dataset. Multiple imputation with chained equations was applied to generate 20 datasets. Baseline demography, ophthalmic history, and recruitment site were used to impute the 77 incomplete values in the outcome variable. Parameter estimates were finally combined using Rubin's principles.

Base ITT model was undertaken as per statistical analysis plan for primary outcome. Models (i) – (iv) are pre-specified and labelled in the protocol and statistical analysis plan. All other models are post-hoc.

Abbreviations: anti-VEGF, anti-Vascular Endothelial Growth Factor; ITT, intention-to-treat model; SRT, stereotactic radiotherapy.

19. **Table S12: Sensitivity analyses of missing data and COVID pandemic impact: primary outcome at Week 192 (year 4)**

		Mean number of injections		Difference (95% confidence interval)	P-Value
		SRT	Sham SRT		
Base Intention-To-Treat (ITT) model		(N=215) 18·4	(N=104) 21·6	-3·2 (-5·7 to -0·8)	0·011*
(i)	All assessments up to primary outcome completed before COVID lockdown [a]	(N=44) 22·4	(N=23) 25·3	-2·9 (-8·3 to 2·5)	0·29
(ii)	At least one assessment up to primary outcome after COVID lockdown [b]	(N=171) 17·1	(N=81) 20·6	-3·5 (-6·2 to -0·6)	0·016*
(iii)	Fully *adherent* population model [c]	(N=192) 18·9	(N=87) 21·7	-2·9 (-5·6 to -0·1)	0·041*
(iv)	Reduced adherence population model [d]	(N=23) 21	(N=17) 21·1	-0·1 (-7·4 to 7·2)	0·98
Base ITT model with additional adjustments [e]		(N=215) 18·4	(N=104) 21·6	-3·2 (-5·7 to -0·8)	0·011*
Per-Protocol model [f]		(N=217) 18·3	(N=102) 21·5	-3·2 (-5·7 to -0·7)	0·012*
Base ITT model with multiple imputation [g]		(N=274) 18·8	(N=137) 21·6	-2·8 (-5 to -0·57)	0·014*

*Table S12: All models are adjusted for baseline stratification factor – National Treatment Centre. The mean in stereotactic radiotherapy (SRT) group is calculated by adding the obtained coefficients to the mean number of injections in the sham SRT group (i.e., adjusted means). *Denotes significant test (p < 0·05).*

v. Main ITT model restricted to participants who completed all 24 visits (4-weekly) before the COVID lockdown 23 March 2020 (population (i) in the manuscript methods section).
w. ITT model restricted to participants who had at least one visit due after lockdown (population (ii) in the manuscript methods section).
x. ITT model restricted to the more adherent population defined as participants who had no more than four non-adherent (red) visits or eight less adherent (amber) visits, and who did not die or withdraw (population (iii) in the manuscript methods section). Definitions of red and amber visits are given in Statistical analysis plan Appendix A; and Table S7, appendix p35-36.
y. ITT model restricted to the less adherent population (population (iv) in the manuscript methods section).
z. Main intention-to-treat (ITT) model with additional adjustment for variables showing significant difference (p < 0·05) between participants with complete primary outcome and those without.
aa. Groups categorized according to the received treatment (i.e., per-protocol analysis) irrespective of adherence.
bb. ITT model on multiply imputed dataset. Multiple imputation with chained equations was applied to generate 20 datasets. Baseline demography, ophthalmic history, and recruitment site were used to impute the 92 incomplete values in the outcome variable. Parameter estimates were finally combined using Rubin's principles.
Base ITT model was undertaken as per statistical analysis plan for primary outcome. Models (i) – (iv) are pre-specified and labelled in the protocol and statistical analysis plan. All other models are post-hoc.

Abbreviations: anti-VEGF, anti-Vascular Endothelial Growth Factor; ITT, intention-to-treat model; SRT, stereotactic radiotherapy.

Clinical efficacy outcomes: Primary and secondary outcomes by sex

20. **Table S13: Primary and secondary efficacy outcomes at Week 96 by sex**

	Male			Female		
	**SRT**	**Sham SRT**	**Adjusted regression coefficient (95% CI)**	**SRT**	**Sham SRT**	**Adjusted regression coefficient (95% CI)**
Number of *prn* anti-VEGF injections, mean (SD)						
Week 48	6·0 (3·4)	7·4 (3·2)	-1·6 (-2·8 to -0·5)	6·2 (3·3)	6·9 (2·8)	-0·8 (-1·6 to 0)
Week 96	10·7 (6·6)	13·8 (6·0)	-3·5 (-5·7 to -1·3)	10·7 (6·1)	12·9 (5·7)	-2·8 (-4·5 to -1·2)
Week 144	14·7 (9·0)	18·9 (8·9)	-4·4 (-7·6 to -1·2)	15·3 (8·6)	17·1 (8·5)	-2·5 (-5 to 0)
Week 192	18·1 (10·6)	23·8 (12·0)	-5·6 (-9·5 to -1·7)	20·0 (11·2)	20·3 (10·7)	-1·9 (-5·3 to 1·4)
Change in ETDRS visual acuity, mean (SD)						
Week 48	0·7 (9·7)	-1·1 (8·0)	0 (-3·1 to 3·1)	-0·5 (8·1)	0·8 (9·2)	-1·5 (-3·9 to 1)
Week 96	-1·5 (10·8)	-3·8 (10·2)	1·1 (-3 to 5·2)	-4·0 (11·2)	-0·2 (11·6)	-3·8 (-7·2 to -0·5)
Week 144	-9·2 (15·8)	-7·3 (14·5)	-3·2 (-9·7 to 3·3)	-10·3 (16·2)	-1·5 (14·2)	-8·9 (-13·7 to -4)
Week 192	-15·0 (17·9)	-12·4 (16·3)	-5 (-11·7 to 1·6)	-16·5 (20·7)	-4·1 (14·7)	-11·2 (-17·2 to -5·1)
ETDRS visual acuity, mean (SD)						
Week 48	67·5 (14·5)	70·2 (13·4)		68·7 (14·4)	69·6 (15·2)	
Week 96	65·7 (13·7)	68·4 (15·0)		65·2 (15·9)	68·6 (16·0)	
Week 144	57·6 (17·8)	64·3 (17·9)		59·8 (19·1)	66·5 (18·5)	
Week 192	51·9 (19·7)	60·0 (19·0)		54·0 (22·2)	64·3 (20·0)	
Losing < 15 ETDRS letters						
Week 48	104 (95%)	47 (94%)		141 (96%)	73 (96%)	
Week 96	92 (89%)	41 (93%)		117 (86%)	71 (93%)	
Week 144	72 (74%)	29 (83%)		88 (67%)	66 (92%)	
Week 192	58 (57%)	24 (67%)		74 (60%)	56 (84%)	
Gaining ≥ 0 ETDRS letters						
Week 48	61 (55%)	23 (46%)		79 (54%)	46 (61%)	
Week 96	54 (52%)	18 (41%)		53 (39%)	40 (53%)	
Week 144	29 (30%)	13 (37%)		44 (34%)	36 (50%)	
Week 192	18 (18%)	7 (19%)		26 (21%)	27 (40%)	
Gaining ≥ 15 ETDRS letters						
Week 48	7 (6%)	1 (2%)		2 (1%)	2 (3%)	
Week 96	4 (4%)	2 (5%)		3 (2%)	1 (1%)	
Week 144	4 (4%)	-		3 (2%)	4 (6%)	
Week 192	1 (<1%)	-		1 (<1%)	4 (6%)	
Total lesion size, median [IQR]						
Week 48	8 [5-12]	6 [3-11]		7 [4-12]	7 [5-12]	
Week 96	7 [4-12]	8 [4-11]		8 [5-12]	7 [4-12]	
Week 144	8 [5-12]	9 [4-15]		9 [5-13]	8 [5-13]	
Week 192	8 [4-12]	8 [3-12]		8 [5-11]	9 [5-15]	
Total active lesion size, median [IQR]						
Week 48	7 [4-11]	6 [3-9]		6 [4-11]	8 [4-12]	
Week 96	6 [4-12]	6 [3-10]		7 [5-12]	6 [4-12]	
Week 144	7 [4-12]	7 [4-13]		9 [6-13]	6 [4-12]	
Week 192	6 [3-12]	7 [3-11]		6 [4-10]	6 [5-11]	
Central subfield thickness (µm), mean (SD)						
Week 48	287·7 (93·3)	343·9 (142·9)		303·2 (124·3)	305·2 (99·3)	
Week 96	309·4 (126·9)	316·0 (108·4)		301·9 (133·6)	302·1 (96·0)	
Week 144	296·7 (122·6)	328·4 (183·3)		283·3 (128·0)	327·4 (274·4)	
Week 192	301·4 (165·5)	269·3 (96·7)		305·0 (217·5)	282·2 (94·0)	
NEI VFQ-25 composite score, median [IQR]						
Week 48	88 [79-95]	92 [84-96]		88 [72-94]	81 [67-93]	
Week 96	89 [76-95]	90 [83-96]		87 [72-94]	81 [60-92]	
Week 144	87 [74-93]	90 [80-95]		86 [63-93]	75 [54-92]	
Week 192	82 [66-92]	88 [79-92]		82 [66-93]	76 [60-92]	
EQ-5D (VAS), median [IQR]						
Week 48	85 [75-95]	85 [80-95]		85 [72-95]	80 [70-90]	

Week 96	85 [75-95]	85 [79-95]		85 [70-95]	80 [70-90]
Week 144	88 [75-90]	88 [75-95]		85 [70-95]	80 [70-90]
Week 192	85 [70-92]	90 [80-96]		80 [69-90]	80 [60-90]

Table S13: Data are n (%), mean (standard deviation) or median (interquartile range). The figures are unadjusted, except for the regression coefficients, which are adjusted for the national treatment centre in the case of the primary outcome, and for both the national treatment centre and baseline visual acuity in the case of the change in visual acuity score. Structural outcomes (lesion size, active lesion size and central subfield thickness) are those from the independent reading centre. Sex was self-reported. This table was prepared at the request of Lancet, during peer review of the primary outcome paper. Abbreviations: EQ-5D-5L (VAS), Euroqol questionnaire with visual analogue scale; ETDRS, Early Treatment Diabetic Retinopathy Study; IQR, interquartile range; NEI VFQ-25, National Eye Institute 25-item visual function questionnaire; prn, pro-re-nata; SD, standard deviation; SRT, stereotactic radiotherapy; VEGF, vascular endothelial growth factor; mm2, millimeter squared; µm, micrometer; 95% CI, 95% confidence interval.

Clinical efficacy outcomes: Number of injections

21. **Table S14: Mean and cumulative number of anti-VEGF injections per 4-weekly visit**

Visit	Number of participants		Mean number of injections per 4 weeks		Cumulative mean number of injections	
	SRT	Sham SRT	SRT	Sham SRT	SRT	Sham SRT
Week 4	273	132	0·73	0·72	0·73	0·72
Week 8	268	126	0·74	0·74	1·48	1·48
Week 12	265	131	0·69	0·71	2·07	2·14
Week 16	252	120	0·67	0·69	2·68	2·76
Week 20	243	118	0·64	0·69	3·21	3·44
Week 24	254	127	0·61	0·65	3·67	3·91
Week 28	259	123	0·59	0·65	4·1	4·57
Week 32	258	121	0·57	0·64	4·54	5·11
Week 36	254	120	0·56	0·64	5	5·73
Week 40	249	114	0·55	0·62	5·45	6·25
Week 44	257	124	0·53	0·61	5·78	6·66
Week 48	259	127	0·51	0·6	6·14	7·17
Week 52	256	117	0·5	0·6	6·55	7·81
Week 56	252	116	0·5	0·6	6·97	8·39
Week 60	250	115	0·49	0·59	7·36	8·9
Week 64	256	120	0·48	0·58	7·64	9·36
Week 68	254	119	0·47	0·58	8·04	9·87
Week 72	250	117	0·47	0·58	8·44	10·41
Week 76	248	113	0·47	0·57	8·85	10·89
Week 80	243	110	0·46	0·57	9·28	11·35
Week 84	247	118	0·46	0·56	9·6	11·84
Week 88	244	116	0·45	0·56	9·98	12·29
Week 92	242	114	0·45	0·55	10·39	12·74
Week 96	241	118	0·45	0·56	10·71	13·33
Week 144	226	108	0·42	0·49	15·03	17·72
Week 192	215	104	0·4	0·45	19·13	21·6

Table S14: Mean and cumulative mean number of anti-VEGF injections (ranibizumab from week 4 – week 92, and which may have been ranibizumab, aflibercept, bevacizumab, brolucizumab or faricimab after week 92), excluding the baseline injection as that reflected pre-existing disease activity and was mandated in all participants. Abbreviations: SRT, stereotactic radiotherapy.

22. **Figure S1: Mean number of anti-VEGF injections for each 4-weekly visit**

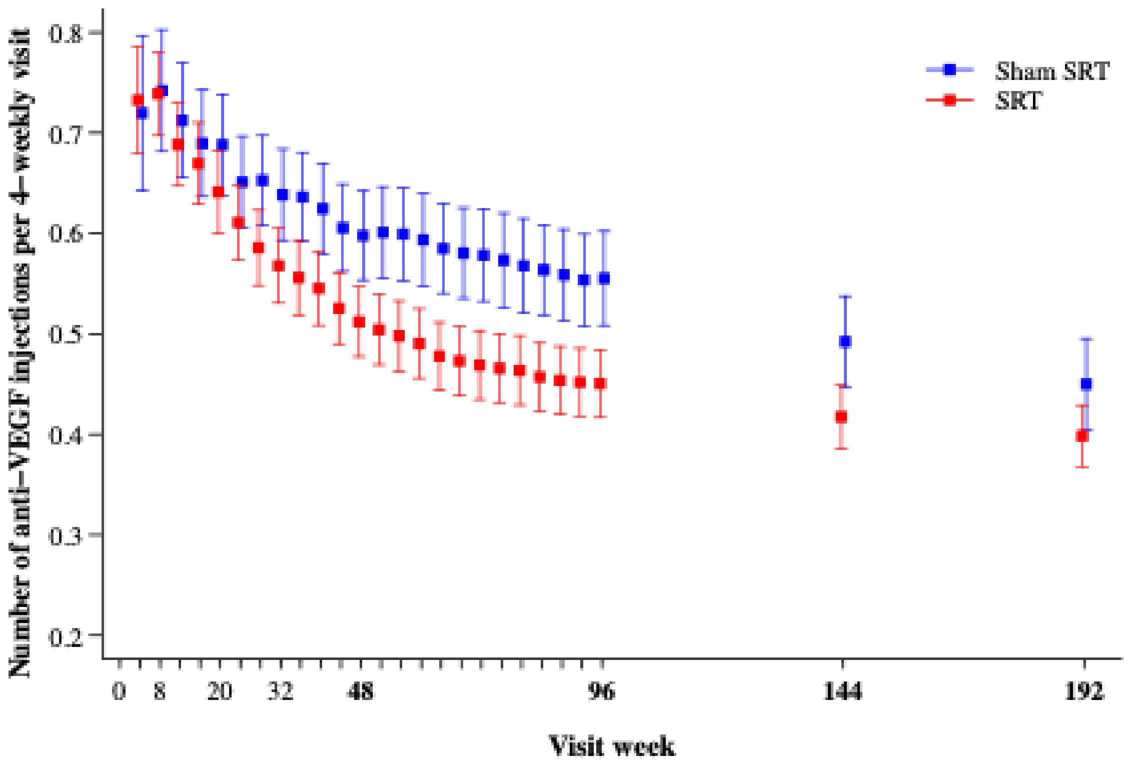

Figure S1: Mean number of anti-VEGF injection (allowed after week 96) from week 4 to week 192, in the stereotactic radiotherapy (SRT) and sham SRT groups. Error bars show the 95% confidence interval. Abbreviations: Abbreviations: SRT, stereotactic radiotherapy

23. **Figure S2: Number of anti-VEGF injections by participants**

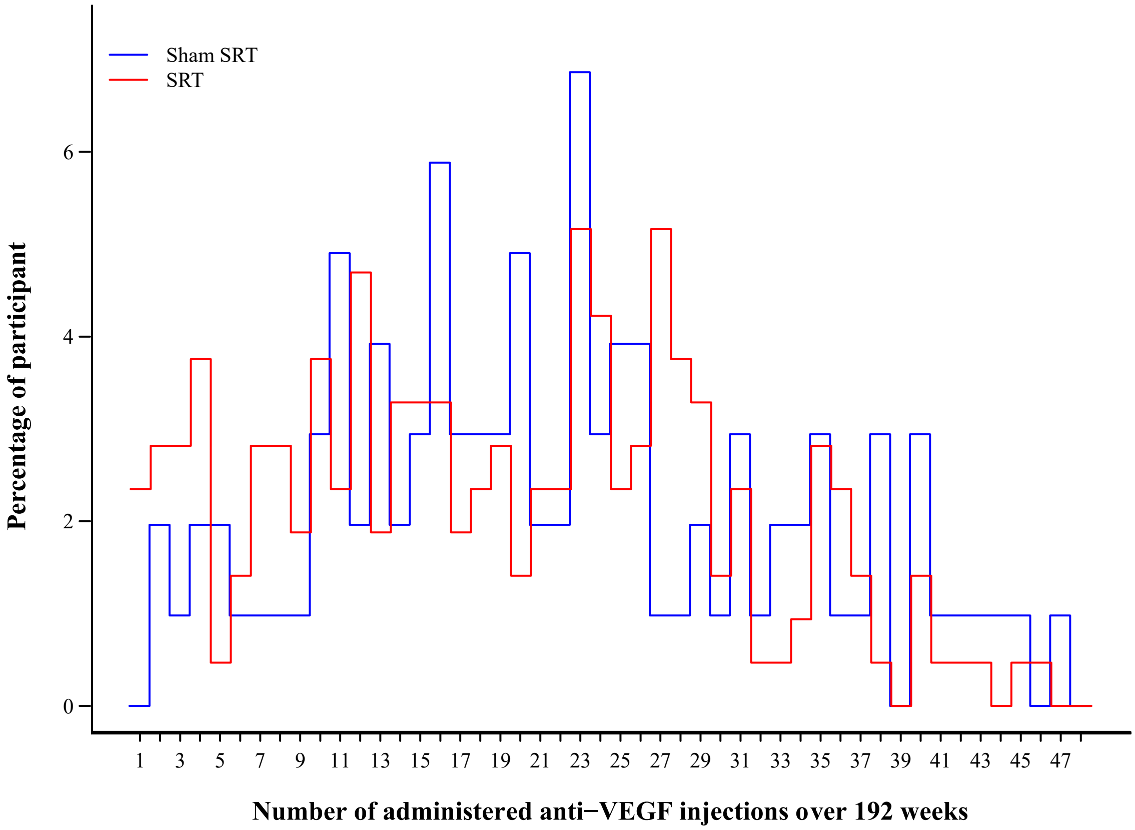

Figure S2: Number of injections participants received over 192 weeks, comparing the stereotactic radiotherapy (SRT) and sham SRT groups. Abbreviations: SRT, stereotactic radiotherapy; VEGF, vascular endothelial growth factor.

Clinical efficacy outcomes: Visual acuity

24. **Figure S3: Mean visual acuity over time**

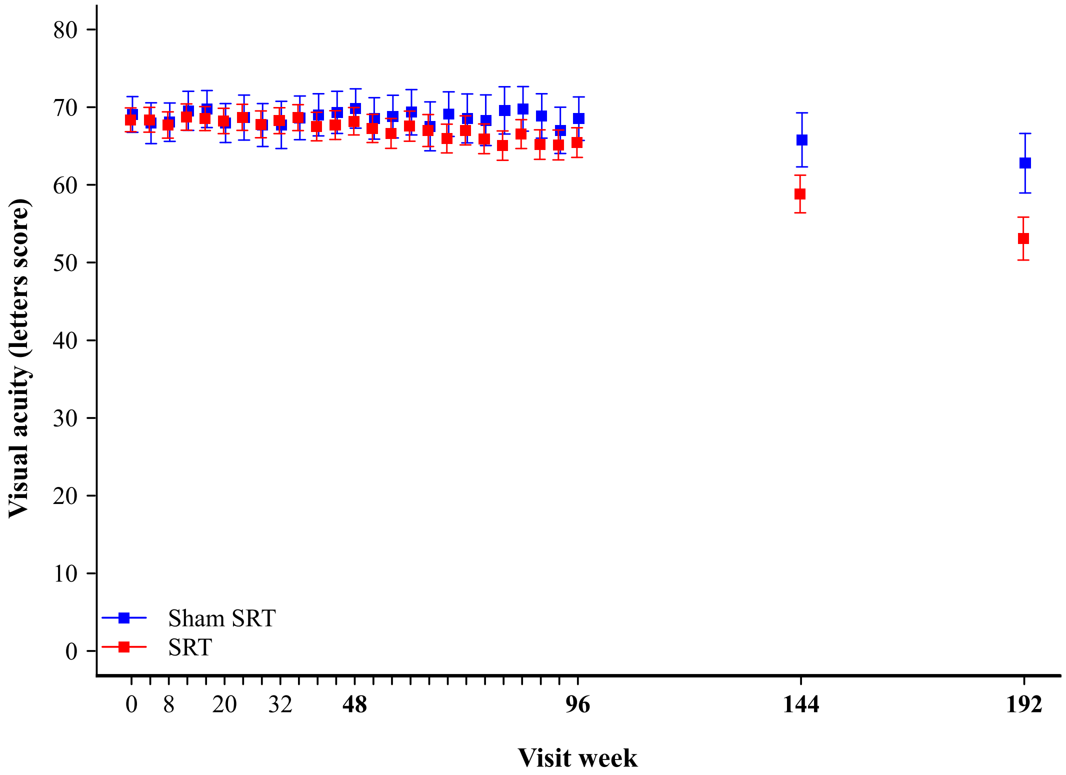

Figure S3: Mean Early Treatment Diabetic Retinopathy Study (ETDRS) letter score at each 4-weekly visit to week 192, comparing the stereotactic radiotherapy (SRT) and sham SRT groups. Error bars show the 95% confidence interval. Abbreviations: SRT, stereotactic radiotherapy.

Clinical efficacy outcomes: Macular thickness

25. **Figure S4: Optical coherence tomography central subfield thickness over time**

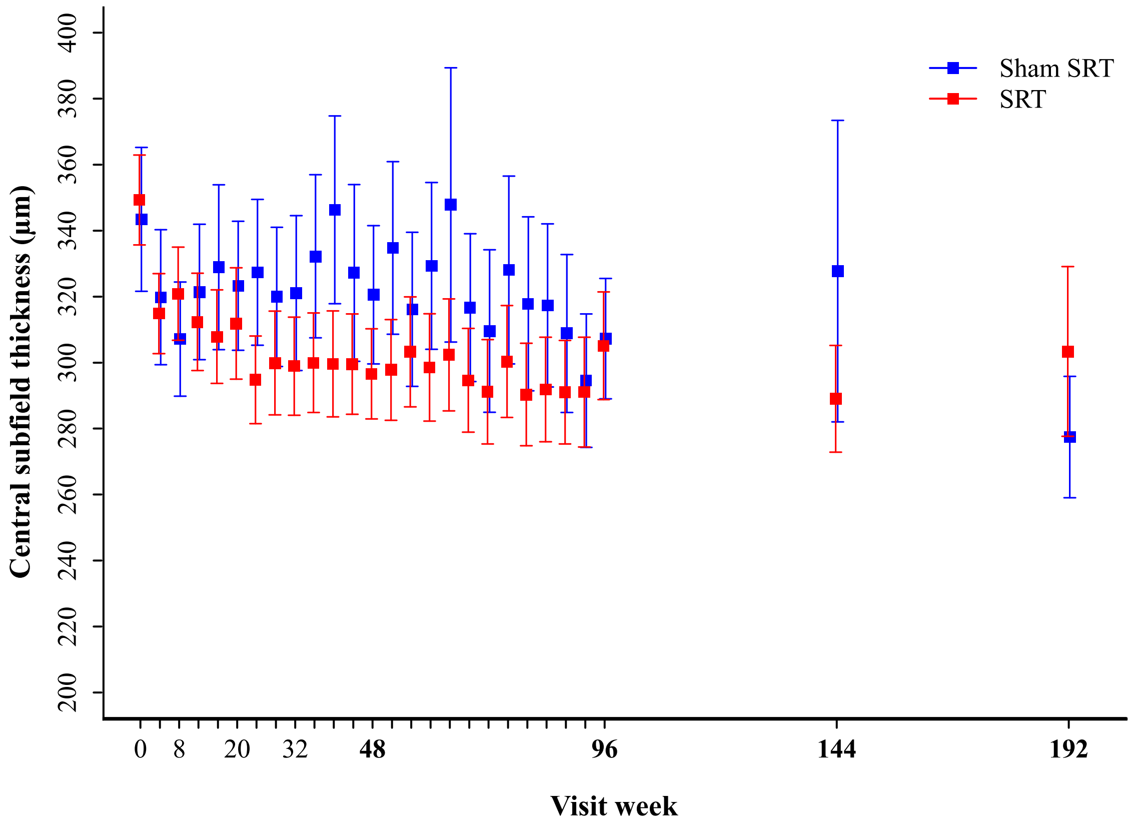

Figure S4: Optical coherence tomography (OCT) central 1mm subfield thickness in the central 1 mm subfield at each 4-weekly visit to week 192, comparing the stereotactic radiotherapy (SRT) and sham SRT groups. Values were determined automatically by each site's OCT device, but masked site clinicians were required to manually correct any segmentation errors. Separate analyses, used for the predefined OCT secondary outcome, were undertaken by the reading centre at baseline and yearly thereafter. Error bars show the 95% confidence interval of the mean. Abbreviations: OCT, optical coherence tomography; SRT, stereotactic radiotherapy; µm, micrometer.

Clinical efficacy outcomes: Reading centre imaging outcomes

26. **Table S15: Reading centre-determined optical coherence tomography analysis**

	SRT	Sham SRT	Difference in means (SRT – Sham)
**Baseline**	N = 274	N = 137	
Full centrepoint thickness, µm	373 (356 to 389)	369 (342 to 395)	4 (-27 to 35)
Neurosensory retinal thickness, µm	258 (246 to 270)	270 (248 to 292)	-12 (-36 to 13)
Subretinal fluid height, µm	92 (80 to 103)	118 (96 to 141)	-26 (-52 to -1·1)
Pigment epithelial detachment height, µm	122 (107 to 138)	101 (80 to 122)	21 (-4·5 to 47)
**Week 48**	N = 259	N = 127	
Full centrepoint thickness, µm	279 (260 to 298)	288 (261 to 315)	-9·4 (-42 to 23)
Neurosensory retinal thickness, µm	196 (182 to 209)	199 (180 to 218)	-3·1 (-27 to 20)
Subretinal fluid height, µm	83 (67 to 100)	89 (71 to 108)	-5·9 (-30 to 18)
Pigment epithelial detachment height, µm	102 (89 to 116)	106 (86 to 126)	-3·9 (-28 to 20)
**Week 96**	N = 241	N = 118	
Full centrepoint thickness, µm	237 (219 to 256)	272 (241 to 303)	-35 (-70 to 0·9)
Neurosensory retinal thickness, µm	174 (160 to 188)	177 (158 to 196)	-3·3 (-27 to 20)
Subretinal fluid height, µm	84 (63 to 104)	94 (70 to 117)	-9·9 (-40 to 21)
Pigment epithelial detachment height, µm	88 (76 to 99)	122 (99 to 145)	-34 (-60 to -8·5)
**Week 144**	N = 233	N = 111	
Full centrepoint thickness, µm	249 (227 to 272)	237 (208 to 265)	13 (-24 to 49)
Neurosensory retinal thickness, µm	182 (166 to 199)	159 (139 to 179)	23 (-2·8 to 50)
Subretinal fluid height, µm	70 (55 to 85)	73 (56 to 89)	-2·6 (-25 to 20)
Pigment epithelial detachment height, µm	93 (78 to 107)	105 (87 to 123)	-12 (-35 to 11)
**Week 192**	N = 222	N = 106	
Full centrepoint thickness, µm	254 (221 to 287)	235 (186 to 284)	19 (-40 to 78)
Neurosensory retinal thickness, µm	193 (167 to 218)	154 (122 to 186)	38 (-1·9 to 79)
Subretinal fluid height, µm	100 (60 to 140)	66 (42 to 90)	34 (-12 to 80)
Pigment epithelial detachment height, µm	92 (76 to 109)	112 (86 to 138)	-19 (-50 to 11)

Table S15: Optical coherence tomography thicknesses measured at the centre of the fovea by the independent reading centre at the respective timepoints. Total centrepoint thickness was measured from the inner limiting membrane to the basement membrane, inclusive of all fovea-involving subretinal fluid and pigment epithelial detachment. Neurosensory retinal thickness was measured from the inner limiting membrane to the outer ellipsoid zone only. Data are mean (95% confidence interval). Abbreviations: SRT, stereotactic radiotherapy; µm, micrometer.

Clinical efficacy outcomes: Subgroup and post-hoc analyses

27. **Figure S5: Difference in number of injections by subgroup at Week 144**

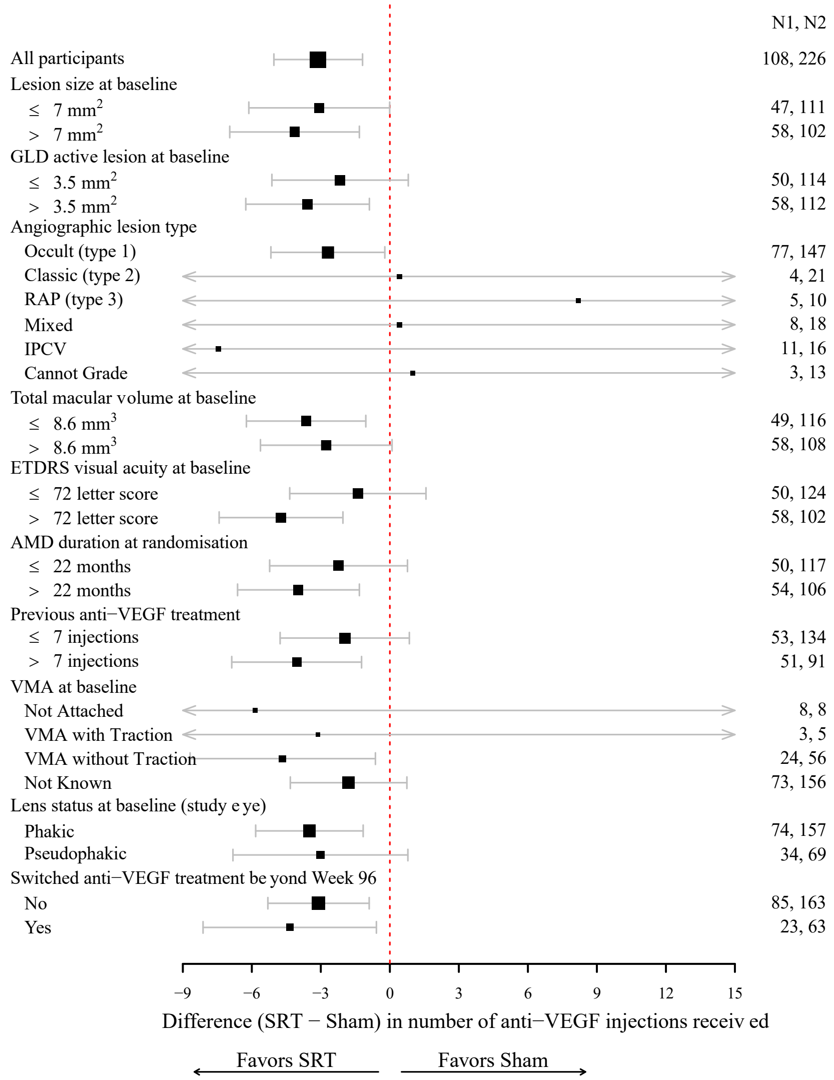

Figure S5: Difference in number of injections by pre-specified subgroup at week 144. N1 = sham-SRT eyes, N2 = SRT eyes. Abbreviations: AMD, age-related macular degeneration; ETDRS, early treatment of diabetic retinopathy study; GLD, greatest linear dimension; IPCV, idiopathic polypoidal choroidal vasculopathy; RAP, retinal angiomatous proliferation; SRT, stereotactic radiotherapy; VEGF, vascular endothelial growth factor; VMA, vitreomacular adhesion; mm^3, millimeters cubed; mm^2, millimetres squared.

28. **Figure S6: Difference in number of injections by subgroup at Week 192**

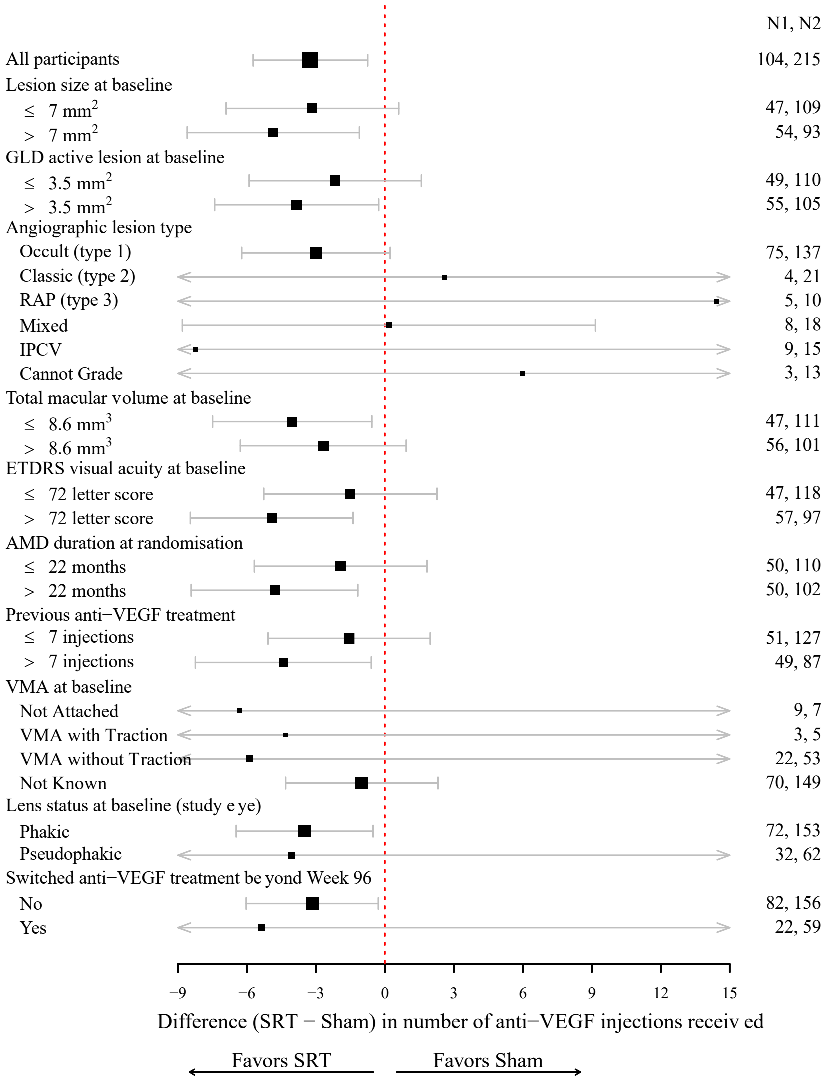

Figure S6: Difference in number of injections by pre-specified subgroup at week 192. N1 = sham-SRT eyes, N2 = SRT eyes. Abbreviations: AMD, age-related macular degeneration; ETDRS, early treatment of diabetic retinopathy study; GLD, greatest linear dimension; IPCV, idiopathic polypoidal choroidal vasculopathy; RAP, retinal angiomatous proliferation; SRT, stereotactic radiotherapy; VEGF, vascular endothelial growth factor; VMA, vitreomacular adhesion; mm^3, millimeters cubed; mm^2, millimetres squared.

29. **Figure S7: Difference in final visual acuity by subgroup at Week 144**

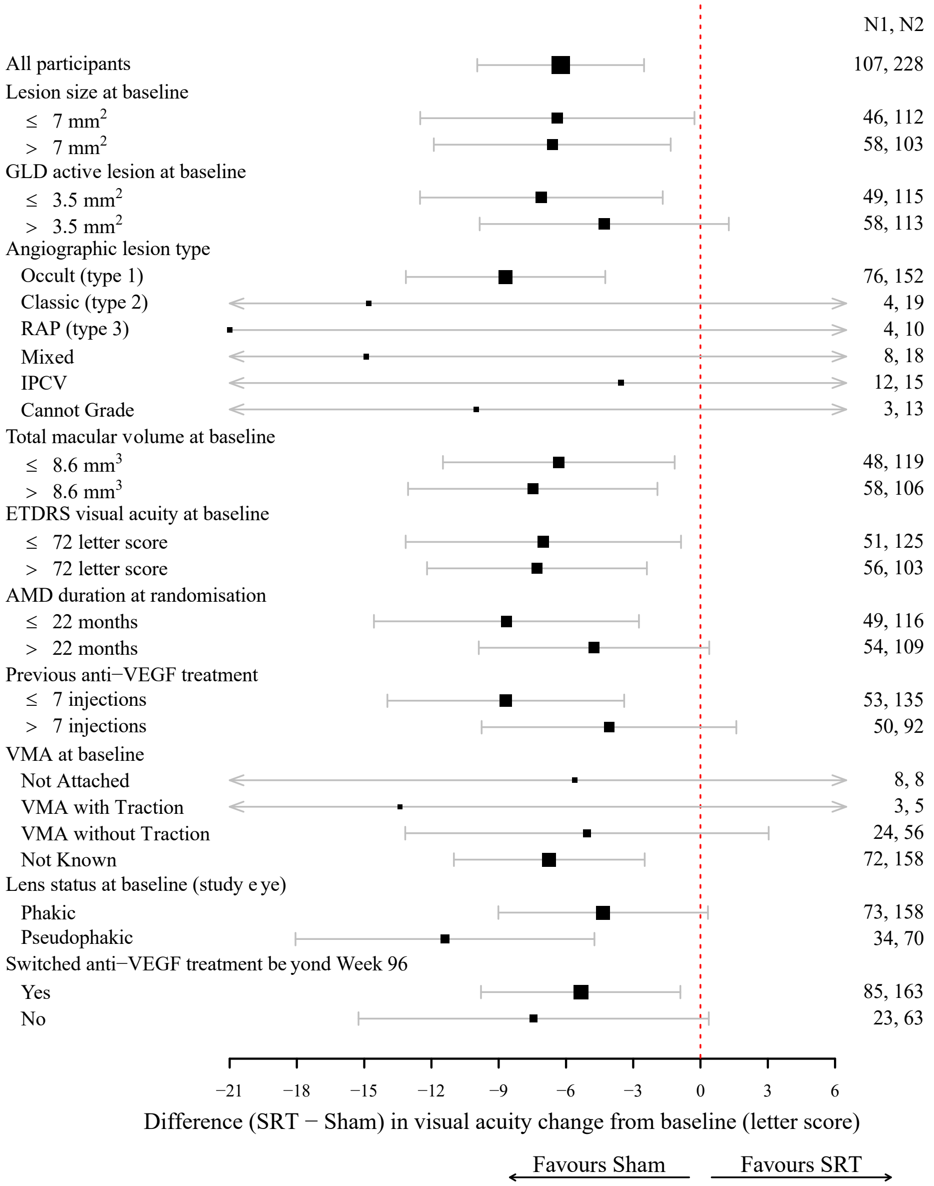

Figure S7: Difference in final visual acuity by pre-specified subgroup at week 144. N1 = sham-SRT eyes, N2 = SRT eyes. Abbreviations: AMD, age-related macular degeneration; ETDRS, early treatment of diabetic retinopathy study; GLD, greatest linear dimension; IPCV, idiopathic polypoidal choroidal vasculopathy; RAP, retinal angiomatous proliferation; SRT, stereotactic radiotherapy; VEGF, vascular endothelial growth factor; VMA, vitreomacular adhesion; mm³, millimeters cubed; mm², millimetres squared..

30. **Figure S8: Difference in final visual acuity by subgroup at Week 192**

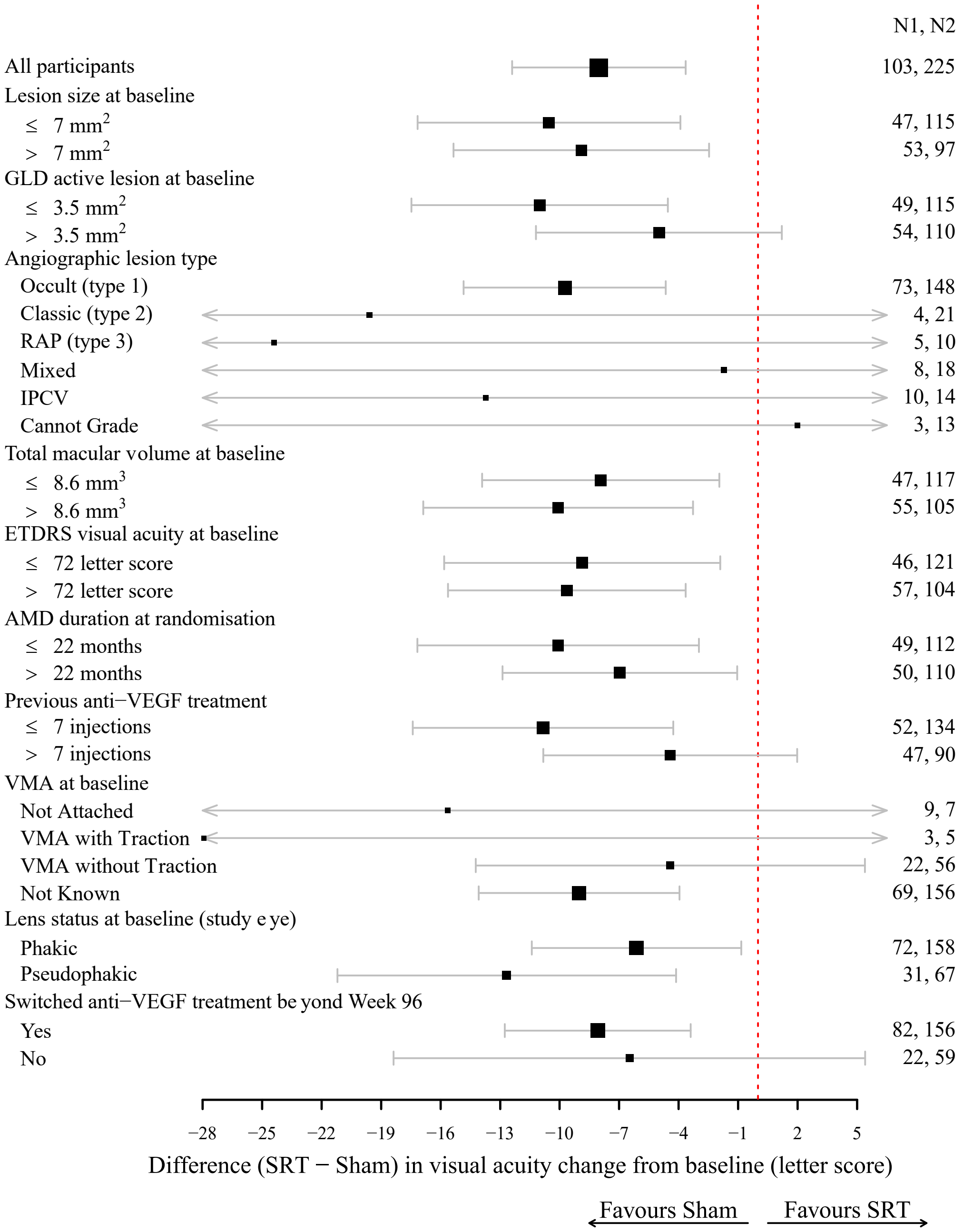

Figure S8: Difference in final visual acuity by subgroup at week 192. N1 = sham-SRT eyes, N2 = SRT eyes. Abbreviations: AMD, age-related macular degeneration; ETDRS, early treatment of diabetic retinopathy study; GLD, greatest linear dimension; IPCV, idiopathic polypoidal choroidal vasculopathy; RAP, retinal angiomatous proliferation; SRT, stereotactic radiotherapy; VEGF, vascular endothelial growth factor; VMA, vitreomacular adhesion; mm³, millimeters cubed; mm², millimetres squared.

31. **Figure S9: Difference in final optical coherence tomography central subfield thickness by subgroup at week 144**

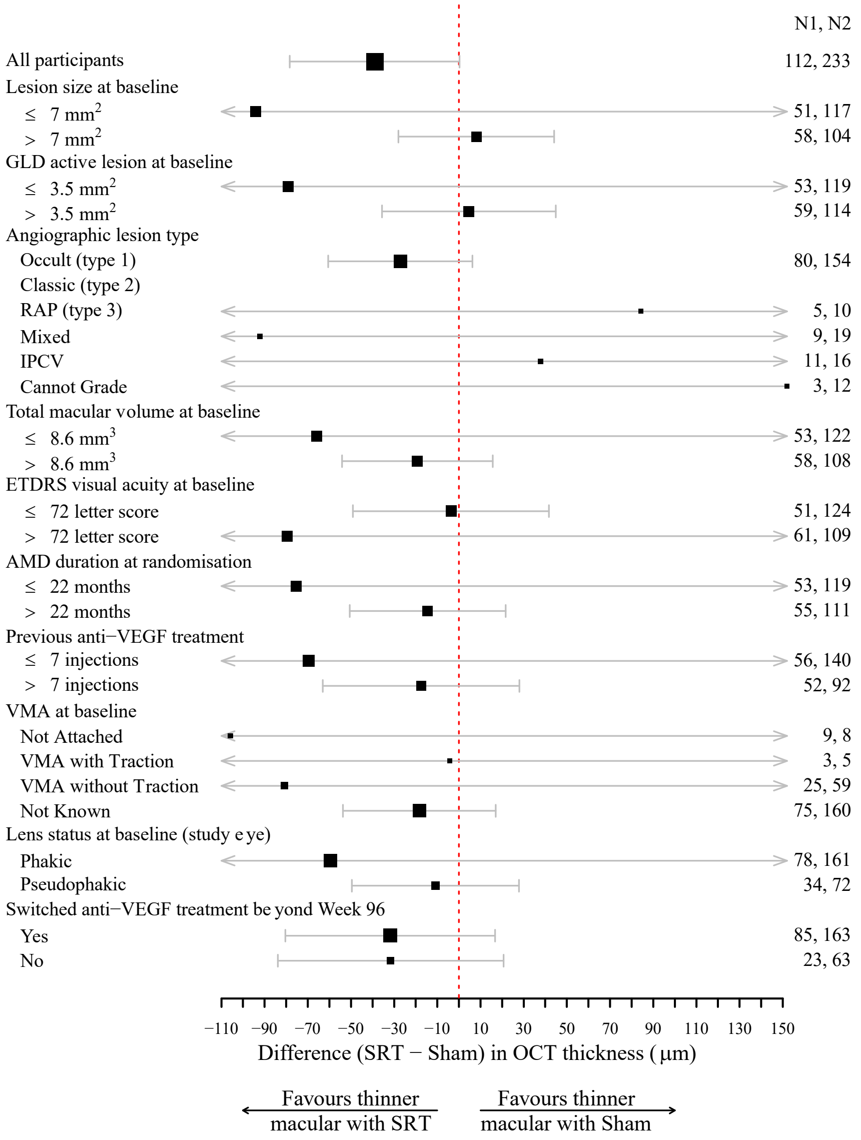

Figure S9: Difference in final optical coherence tomography central subfield thickness by subgroup at week 144. N1 = sham-SRT eyes, N2 = SRT eyes. Abbreviations: AMD, age-related macular degeneration; ETDRS, early treatment of diabetic retinopathy study; GLD, greatest linear dimension; IPCV, idiopathic polypoidal choroidal vasculopathy; RAP, retinal angiomatous proliferation; SRT, stereotactic radiotherapy; VEGF, vascular endothelial growth factor; VMA, vitreomacular adhesion; mm³, millimeters cubed; mm², millimetres squared.

32. Figure S10: Difference in final optical coherence tomography central subfield thickness by subgroup at week 192

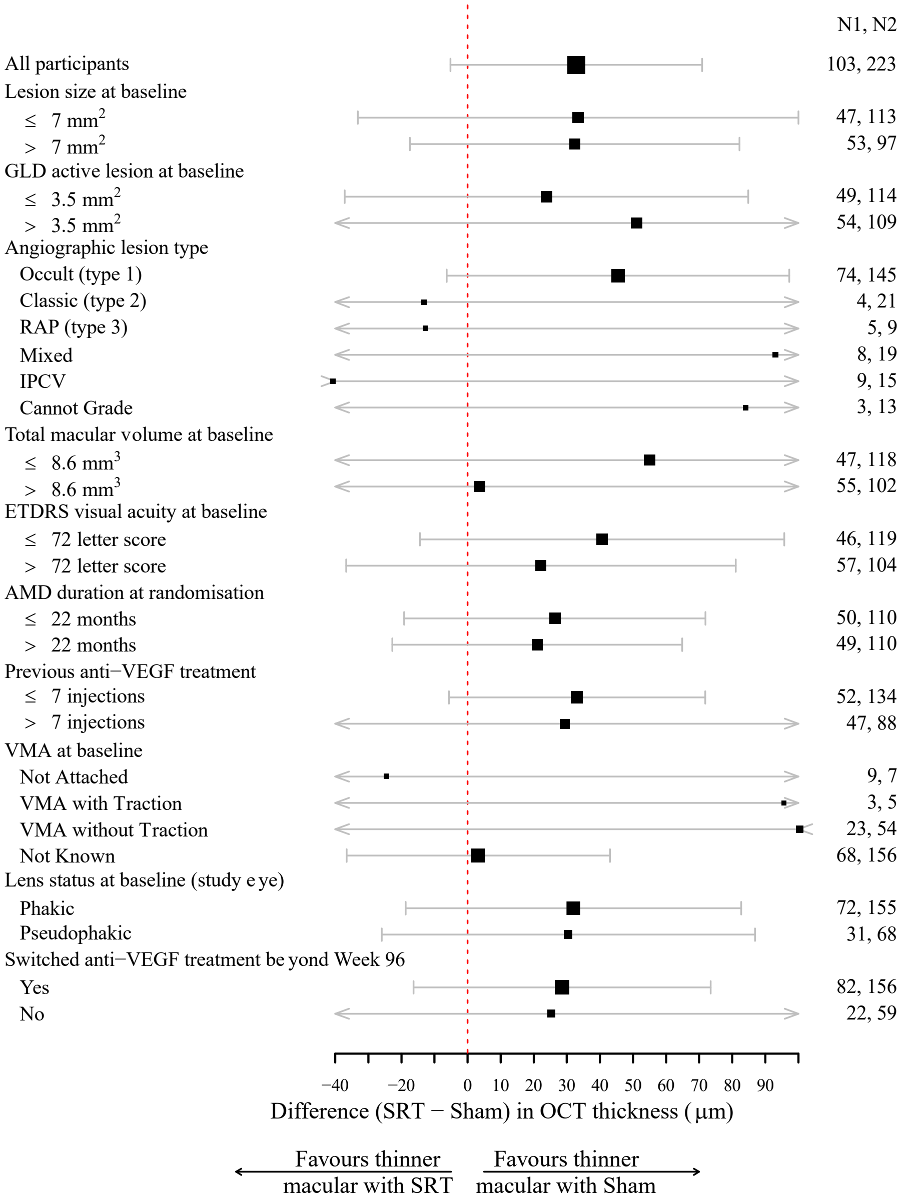

Figure S10: Difference in final optical coherence tomography central subfield thickness by subgroup at week 192. N1 = sham-SRT eyes, N2 = SRT eyes. Abbreviations: AMD, age-related macular degeneration; ETDRS, early treatment of diabetic retinopathy study; GLD, greatest linear dimension; IPCV, idiopathic polypoidal choroidal vasculopathy; RAP, retinal angiomatous proliferation; SRT, stereotactic radiotherapy; VEGF, vascular endothelial growth factor; VMA, vitreomacular adhesion; mm³, millimeters cubed; mm², millimetres squared.

33. **Table S16: Outcomes of visual acuity and VFQ-25 composite scores by study eye being better/worse seeing eye at baseline**

	Better seeing eyes		Worse seeing eyes	
	**SRT (N=87)**	**Sham SRT (N=53)**	**SRT (N=180)**	**Sham SRT (N=80)**
**Week 48**				
ETDRS visual acuity (letters score)	72·1 (10·3)	73·4 (10·4)	66·1 (15·8)	67·9 (16·5)
NEI VFQ-25 composite score	78 [62-90]	81 [67-90]	91 [82-95]	91 [80-96]
Agreement in VA and VFQ-25 changes from baseline	37 (45%)	24 (55%)	85 (53%)	41 (55%)
**Week 96**				
ETDRS visual acuity (letters score)	70·2 (9·4)	71·7 (14·7)	63·3 (16·6)	66·6 (15·9)
NEI VFQ-25 composite score	78 [64-90]	79 [55-90]	90 [80-95]	91 [81-96]
Agreement in VA and VFQ-25 changes from baseline	30 (43%)	22 (44%)	91 (57%)	34 (52%)
**Week 144**				
ETDRS visual acuity (letters score)	64·9 (14·0)	70·6 (13·4)	56·2 (19·8)	63·6 (19·8)
NEI VFQ-25 composite score	74 [57-86]	72 [55-89]	90 [79-94]	90 [75-95]
Agreement in VA and VFQ-25 changes from baseline	42 (60%)	18 (39%)	80 (54%)	25 (38%)
**Week 192**				
ETDRS visual acuity (letters score)	59·2 (18·6)	69·1 (12·8)	51·1 (21·0)	59·4 (21·7)
NEI VFQ-25 composite score	70 [50-84]	75 [52-84]	87 [72-94]	88 [75-94]
Agreement in VA and VFQ-25 changes from baseline	36 (54%)	23 (55%)	83 (57%)	40 (65%)

Table S16: Outcomes of visual acuity and VFQ-25 composite scores disaggregated by whether study eye was the better or worse seeing eye at baseline. Abbreviations: ETDRS, early treatment of diabetic retinopathy study; NEI VFQ-25, National Eye Institute Visual Function Questionnaire-25 item; SRT, stereotactic radiotherapy; VA, visual acuity.

34. **Table S17: Outcomes of visual acuity and VFQ-25 composite scores by completeness of outcomes during COVID-19 lockdown**

	Attended any visit during COVID-19 lockdown			
	No		Yes	
	**SRT**	**Sham SRT**	**SRT**	**Sham SRT**
**Week 48**	**(N=228)**	**(N=113)**	**(N=46)**	**(N=24)**
ETDRS visual acuity (letters score)	68·2 (14·8)	71·4 (13·3)	68·1 (12·4)	61·2 (18·0)
NEI VFQ-25 composite score	88·3 [77·0-94·8]	88·1 [73·9-94·4]	88·4 [75·9-92·4]	72·9 [62·0-94·4]
**Week 96**	**(N=167)**	**(N=85)**	**(N=107)**	**(N=52)**
ETDRS visual acuity (letters score)	65·5 (15·8)	70·1 (14·3)	65·3 (13·3)	65·8 (17·4)
NEI VFQ-25 composite score	89·2 [76·0-95·2]	84·4 [66·6-94·0]	85·8 [70·2-91·9]	87·1 [64·0-93·5]
**Week 144**	**(N=100)**	**(N=50)**	**(N=174)**	**(N=87)**
ETDRS visual acuity (letters score)	57·8 (19·7)	64·5 (21·2)	59·4 (17·9)	66·6 (16·3)
NEI VFQ-25 composite score	89·3 [76·8-93·5]	87·7 [56·6-94·5]	82·8 [63·8-92·6]	80·4 [66·0-92·1]
**Week 192**	**(N=54)**	**(N=27)**	**(N=220)**	**(N=110)**
ETDRS visual acuity (letters score)	55·8 (17·7)	65·0 (14·5)	52·4 (21·8)	62·1 (21·0)
NEI VFQ-25 composite score	84·2 [74·5-92·7]	73·8 [60·4-86·4]	81·5 [61·1-92·0]	84·3 [67·0-92·9]

Table S17: Outcomes of visual acuity and VFQ-25 composite scores disaggregated by whether key study visits (weeks 48, 96, 144 and 192) were undertaken during COVID lockdown. Abbreviations: ETDRS, early treatment of diabetic retinopathy study; NEI VFQ-25, National Eye Institute Visual Function Questionnaire-25 item; SRT, stereotactic radiotherapy.

35. **Table S18: Outcomes of visual acuity and VFQ-25 composite scores by presence of visually significant lens opacity**

	Significant opacity in the study eye at visit week			
	No		Yes	
	**SRT**	**Sham**	**SRT**	**Sham**
**Week 48**	**(N=206)**	**(N=105)**	**(N=39)**	**(N=15)**
ETDRS visual acuity (letters score)	68·9 (14·5)	70·2 (15·0)	63·3 (14·2)	67·7 (12·0)

NEI VFQ-25 composite score	89 [78-95]	87 [74-94]	86 [63-92]	90 [74-96]
**Week 96**	**(N=199)**	**(N=100)**	**(N=34)**	**(N=13)**
ETDRS visual acuity (letters score)	66·0 (15·1)	68·6 (16·2)	60·5 (14·0)	65·2 (13·7)
NEI VFQ-25 composite score	89 [74-95]	86 [66-93]	84 [65-90]	91 [67-95]
**Week 144**	**(N=184)**	**(N=93)**	**(N=35)**	**(N=17)**
ETDRS visual acuity (letters score)	60·1 (17·9)	65·6 (18·7)	52·4 (20·4)	66·7 (16·4)
NEI VFQ-25 composite score	85 [67-93]	82 [64-94]	89 [65-94]	85 [62-92]
**Week 192**	**(N=182)**	**(N=75)**	**(N=28)**	**(N=19)**
ETDRS visual acuity (letters score)	53·6 (21·2)	63·3 (19·0)	48·8 (21·6)	64·1 (20·2)
NEI VFQ-25 composite score	82 [66-92]	80 [65-92]	88 [58-94]	90 [73-92]

Table S18: Outcomes of visual acuity and VFQ-25 composite scores disaggregated by whether there was a visually significant lens opacity at weeks 48, 96, 144 and 192. Abbreviations: ETDRS, early treatment of diabetic retinopathy study; NEI VFQ-25, National Eye Institute Visual Function Questionnaire-25 item; SRT, stereotactic radiotherapy.

36. **Table S19: Outcomes of visual acuity and VFQ-25 composite scores by presence of subfoveal fibrosis, subfoveal atrophy, and ellipsoid zone disruption**

	Subfoveal fibrosis at visit week			
	No		Yes	
	**SRT**	**Sham**	**SRT**	**Sham**
**Week 48**	**(N=14)**	**(N=2)**	**(N=29)**	**(N=8)**
ETDRS visual acuity (letters score)	60·6 (15·3)	54·5 (4·9)	64·1 (17·2)	60·8 (21·0)
NEI VFQ-25 composite score	88 [73-96]	93 [90-96]	80 [66-91]	87 [70-96]
**Week 96**	**(N=16)**	**(N=6)**	**(N=50)**	**(N=18)**
ETDRS visual acuity (letters score)	63·8 (15·3)	70·0 (10·2)	66·2 (16·0)	66·3 (15·2)
NEI VFQ-25 composite score	88 [75-94]	87 [69-91]	88 [69-95]	87 [70-95]
**Week 144**	**(N=12)**	**(N=9)**	**(N=46)**	**(N=20)**
ETDRS visual acuity (letters score)	51·7 (20·9)	57·3 (23·5)	58·8 (16·5)	58·1 (18·1)
NEI VFQ-25 composite score	88 [77-93]	75 [70-90]	80 [62-92]	90 [60-95]
**Week 192**	**(N=16)**	**(N=4)**	**(N=62)**	**(N=27)**
ETDRS visual acuity (letters score)	45·8 (24·2)	61·5 (16·8)	51·6 (19·5)	56·4 (24·9)
NEI VFQ-25 composite score	65 [47-83]	72 [45-92]	82 [67-93]	80 [59-90]

	Subfoveal atrophy at visit week			
	No		Yes	
	**SRT**	**Sham**	**SRT**	**Sham**
**Week 48**	**(N=31)**	**(N=14)**	**(N=42)**	**(N=14)**
ETDRS visual acuity (letters score)	69·7 (11·3)	69·4 (13·4)	61·3 (17·1)	64·9 (20·7)
NEI VFQ-25 composite score	88 [74-94]	87 [66-89]	83 [68-91]	84 [54-94]
**Week 96**	**(N=34)**	**(N=16)**	**(N=47)**	**(N=15)**
ETDRS visual acuity (letters score)	63·6 (16·3)	66·1 (13·9)	59·2 (16·9)	67·9 (17·2)
NEI VFQ-25 composite score	84 [67-93]	63 [44-91]	84 [65-91]	86 [63-92]
**Week 144**	**(N=26)**	**(N=13)**	**(N=57)**	**(N=18)**
ETDRS visual acuity (letters score)	55·0 (15·4)	68·1 (17·5)	51·8 (20·8)	61·8 (14·6)
NEI VFQ-25 composite score	75 [52-88]	89 [75-94]	79 [55-90]	72 [53-94]
**Week 192**	**(N=29)**	**(N=10)**	**(N=67)**	**(N=21)**
ETDRS visual acuity (letters score)	54·7 (21·3)	63·7 (15·6)	48·6 (19·9)	65·2 (16·4)
NEI VFQ-25 composite score	77 [61-90]	74 [68-84]	76 [63-90]	82 [70-94]

	Ellipsoid zone disruption at visit week			
	No		Yes	
	**SRT**	**Sham**	**SRT**	**Sham**
**Week 48**	**(N=30)**	**(N=26)**	**(N=228)**	**(N=96)**
ETDRS visual acuity (letters score)	68·9 (14·3)	70·8 (12·2)	68·3 (14·0)	70·0 (14·6)
NEI VFQ-25 composite score	88 [77-93]	89 [79-95]	89 [77-95]	86 [72-94]
**Week 96**	**(N=37)**	**(N=14)**	**(N=195)**	**(N=99)**
ETDRS visual acuity (letters score)	67·0 (12·6)	68·9 (13·1)	65·5 (15·2)	69·4 (14·8)

NEI VFQ-25 composite score	88 [79-95]	86 [66-90]	88 [72-94]	85 [67-94]
**Week 144**	**(N=28)**	**(N=21)**	**(N=200)**	**(N=85)**
ETDRS visual acuity (letters score)	62·0 (17·6)	72·1 (10·7)	58·5 (18·5)	64·6 (18·3)
NEI VFQ-25 composite score	87 [71-92]	85 [76-92]	87 [68-94]	85 [61-94]
**Week 192**	**(N=29)**	**(N=14)**	**(N=191)**	**(N=88)**
ETDRS visual acuity (letters score)	66·2 (19·2)	70·0 (9·8)	51·5 (20·5)	62·3 (19·8)
NEI VFQ-25 composite score	86 [69-95]	80 [74-85]	82 [64-92]	84 [66-93]

Table S19: Outcomes of visual acuity and VFQ-25 composite scores disaggregated by whether there was reading-centre determined subfoveal fibrosis, atrophy or ellipsoid zone disruption at weeks 48, 96, 144 and 192. Abbreviations: ETDRS, early treatment of diabetic retinopathy study; NEI VFQ-25, National Eye Institute Visual Function Questionnaire-25 item; SRT, stereotactic radiotherapy.

37. **Table S20: Outcomes of visual acuity and VFQ-25 composite scores by presence of active exudative AMD, total lesion area, and total active lesion area**

	**Active exudative AMD at visit week**			
	**No**		**Yes**	
	**SRT**	**Sham**	**SRT**	**Sham**
**Week 48**	**(N=54)**	**(N=14)**	**(N=172)**	**(N=93)**
ETDRS visual acuity (letters score)	65·6 (16·7)	64·4 (18·6)	68·7 (14·1)	72·4 (12·8)
NEI VFQ-25 composite score	85 [70-93]	85 [73-97]	89 [79-95]	88 [73-94]
**Week 96**	**(N=71)**	**(N=21)**	**(N=136)**	**(N=81)**
ETDRS visual acuity (letters score)	64·5 (17·5)	64·8 (18·4)	66·0 (13·3)	70·8 (12·2)
NEI VFQ-25 composite score	87 [71-94]	84 [63-93]	88 [75-95]	86 [68-94]
**Week 144**	**(N=74)**	**(N=28)**	**(N=130)**	**(N=68)**
ETDRS visual acuity (letters score)	58·1 (18·3)	59·9 (19·7)	58·8 (18·9)	68·9 (15·9)
NEI VFQ-25 composite score	80 [58-92]	76 [60-92]	88 [72-94]	86 [70-94]
**Week 192**	**(N=93)**	**(N=35)**	**(N=101)**	**(N=57)**
ETDRS visual acuity (letters score)	54·0 (19·4)	62·8 (22·8)	52·6 (22·1)	65·2 (16·1)
NEI VFQ-25 composite score	80 [64-92]	79 [64-90]	85 [70-93]	85 [72-93]

Table S20: Outcomes of visual acuity and VFQ-25 composite scores disaggregated by whether there was presence of active exudative AMD at weeks 48, 96, 144 and 192. Abbreviations: AMD, age-related macular degeneration; ETDRS, early treatment of diabetic retinopathy study; NEI VFQ-25, National Eye Institute Visual Function Questionnaire-25 item; SRT, stereotactic radiotherapy.

38. **Table S21: Outcomes of visual acuity and VFQ-25 composite scores disaggregated by median total lesion area, and total active lesion area**

	**Total lesion area at visit week**			
	**≤ median**		**> median**	
	**SRT**	**Sham**	**SRT**	**Sham**
**Week 48**	**(N=112)**	**(N=55)**	**(N=114)**	**(N=52)**
ETDRS visual acuity (letters score)	71·9 (10·0)	71·3 (14·6)	64·1 (17·5)	71·4 (13·2)
NEI VFQ-25 composite score	91 [82-96]	92 [81-96]	84 [73-94]	84 [70-92]
**Week 96**	**(N=100)**	**(N=54)**	**(N=107)**	**(N=48)**
ETDRS visual acuity (letters score)	70·1 (10·5)	70·3 (13·4)	61·2 (17·0)	68·7 (14·4)
NEI VFQ-25 composite score	89 [79-95]	88 [72-96]	85 [70-93]	85 [66-92]
**Week 144**	**(N=103)**	**(N=47)**	**(N=101)**	**(N=49)**
ETDRS visual acuity (letters score)	63·3 (16·2)	71·3 (14·3)	53·6 (19·7)	61·3 (19·0)
NEI VFQ-25 composite score	88 [71-95]	86 [77-95]	83 [63-92]	75 [61-94]
**Week 192**	**(N=101)**	**(N=43)**	**(N=93)**	**(N=49)**
ETDRS visual acuity (letters score)	58·3 (17·6)	67·1 (16·1)	47·8 (22·7)	61·9 (20·8)
NEI VFQ-25 composite score	85 [72-93]	86 [78-94]	78 [58-91]	78 [60-92]

	**Total active lesion area at visit week**

	≤ median		> median	
	**SRT**	**Sham**	**SRT**	**Sham**
**Week 48**	**(N=85)**	**(N=48)**	**(N=88)**	**(N=45)**
ETDRS visual acuity (letters score)	71·0 (10·8)	72·3 (13·0)	66·5 (16·4)	72·5 (12·7)
NEI VFQ-25 composite score	92 [83-96]	89 [81-95]	84 [74-94]	86 [70-93]
**Week 96**	**(N=65)**	**(N=43)**	**(N=71)**	**(N=40)**
ETDRS visual acuity (letters score)	68·2 (11·0)	71·3 (12·0)	64·0 (14·9)	69·8 (13·1)
NEI VFQ-25 composite score	90 [84-96]	84 [73-95]	85 [70-93]	88 [68-94]
**Week 144**	**(N=61)**	**(N=38)**	**(N=69)**	**(N=30)**
ETDRS visual acuity (letters score)	63·3 (16·6)	72·2 (15·1)	54·6 (19·9)	64·5 (16·2)
NEI VFQ-25 composite score	89 [76-95]	90 [83-95]	88 [70-93]	72 [57-92]
**Week 192**	**(N=52)**	**(N=27)**	**(N=49)**	**(N=30)**
ETDRS visual acuity (letters score)	58·2 (17·4)	67·8 (13·4)	46·8 (25·0)	63·0 (18·0)
NEI VFQ-25 composite score	89 [72-93]	90 [79-95]	80 [66-92]	81 [65-92]

Table S21: Outcomes of visual acuity and VFQ-25 composite scores disaggregated by reading-centre determined median total and active lesion areas (see Table 2) at weeks 48, 96, 144 and 192. Abbreviations: ETDRS, early treatment of diabetic retinopathy study; NEI VFQ-25, National Eye Institute Visual Function Questionnaire-25 item; SRT, stereotactic radiotherapy.

39. Table S22: Outcomes of visual acuity and VFQ-25 composite scores by presence of macular co-pathology

	Pathology present			
	No		Yes	
	**SRT**	**Sham**	**SRT**	**Sham**
**Week 48**	**(N=167)**	**(N=82)**	**(N=92)**	**(N=41)**
ETDRS visual acuity (letters score)	69·3 (13·5)	72·1 (12·3)	66·1 (15·9)	66·5 (16·4)
NEI VFQ-25 composite score	90 [74-96]	88 [74-95]	87 [79-92]	88 [68-94]
**Week 96**	**(N=153)**	**(N=78)**	**(N=81)**	**(N=35)**
ETDRS visual acuity (letters score)	66·5 (13·4)	71·2 (13·3)	64·1 (17·0)	65·3 (16·4)
NEI VFQ-25 composite score	89 [76-95]	88 [68-94]	86 [71-93]	82 [62-94]
**Week 144**	**(N=122)**	**(N=73)**	**(N=106)**	**(N=33)**
ETDRS visual acuity (letters score)	59·4 (20·0)	70·2 (15·1)	58·3 (16·3)	57·7 (18·7)
NEI VFQ-25 composite score	88 [74-93]	85 [67-94]	81 [66-93]	85 [61-94]
**Week 192**	**(N=136)**	**(N=67)**	**(N=86)**	**(N=36)**
ETDRS visual acuity (letters score)	55·9 (20·2)	64·2 (18·2)	49·2 (21·9)	61·9 (19·9)
NEI VFQ-25 composite score	82 [62-92]	83 [66-93]	83 [70-92]	80 [66-92]

	Epiretinal membrane present			
	No		Yes	
	**SRT**	**Sham**	**SRT**	**Sham**
**Week 48**	**(N=188)**	**(N=100)**	**(N=86)**	**(N=37)**
ETDRS visual acuity (letters score)	69·4 (13·3)	71·4 (13·0)	65·7 (16·2)	66·1 (17·1)
NEI VFQ-25 composite score	89 [74-96]	88 [74-94]	88 [79-92]	87 [67-94]
**Week 96**	**(N=198)**	**(N=105)**	**(N=76)**	**(N=32)**
ETDRS visual acuity (letters score)	65·8 (13·8)	69·9 (14·9)	64·6 (17·3)	64·7 (17·0)
NEI VFQ-25 composite score	89 [76-95]	87 [68-93]	86 [70-93]	82 [59-95]
**Week 144**	**(N=176)**	**(N=107)**	**(N=98)**	**(N=30)**
ETDRS visual acuity (letters score)	58·8 (20·1)	69·5 (16·6)	58·8 (16·3)	56·4 (19·1)
NEI VFQ-25 composite score	87 [70-93]	82 [66-93]	81 [67-93]	87 [60-94]
**Week 192**	**(N=200)**	**(N=104)**	**(N=74)**	**(N=33)**
ETDRS visual acuity (letters score)	54·6 (20·0)	63·4 (19·4)	49·9 (23·0)	61·5 (20·6)
NEI VFQ-25 composite score	82 [63-92]	81 [65-92]	83 [70-91]	82 [66-92]

Table S22: Outcomes of visual acuity and VFQ-25 composite scores disaggregated by reading-centre determined presence or absence of macular co-pathology at weeks 48, 96, 144 and 192. The most common co-pathology was epiretinal membrane and the sub-table explored visual and VFQ-25 only in eyes with or without ERM. Abbreviations: ETDRS, early treatment of diabetic retinopathy study; NEI VFQ-25, National Eye Institute Visual Function Questionnaire-25 item; SRT, stereotactic radiotherapy.

40. **Figure S11: Differences in ETDRS best-corrected visual acuity in SRT group study eyes disaggregated by explanatory causes for vision loss, at each timepoint.**

Visit	Patients N1	Patients N2	Difference (N2–N1) in BCVA (95% CI)
By study eye being better/worse seeing eye at baseline			
Week 48	180	87	5·9 (2·2 to 9·7)
Week 96	180	87	6·9 (2·8 to 11·0)
Week 144	180	87	8·7 (3·6 to 13·8)
Week 192	180	87	8·0 (2·2 to 13·8)
By having attended any visit during COVID−19 lockdown			
Week 48	228	46	−0·15 (−5·3 to 5·0)
Week 96	167	107	−0·20 (−4·2 to 3·8)
Week 144	100	174	1·6 (−3·4 to 6·6)
Week 192	54	220	−3·3 (−10·3 to 3·6)
By presence of significant opacity in the study eye			
Week 48	206	39	−5·6 (−10·6 to −0·66)
Week 96	199	34	−5·5 (−11·0 to −0·02)
Week 144	184	35	−7·7 (−14·5 to −0·99)
Week 192	182	28	−4·9 (−13·4 to 3·6)
By presence of fibrosis			
Week 48	154	65	−7·6 (−11·8 to −3·4)
Week 96	112	92	−1·3 (−5·5 to 2·8)
Week 144	115	85	−4·1 (−9·4 to 1·2)
Week 192	92	102	−9·3 (−15·2 to −3·4)
By presence of subfoveal fibrosis			
Week 48	14	29	3·5 (−7·5 to 14·4)
Week 96	16	50	2·4 (−6·7 to 11·5)
Week 144	12	46	7·2 (−4·2 to 18·5)
Week 192	16	62	5·8 (−5·7 to 17·2)
By presence of atrophy			
Week 48	130	93	−5·1 (−9·0 to −1·3)
Week 96	111	92	−7·1 (−11·1 to −3·1)
Week 144	98	102	−10·3 (−15·3 to −5·2)
Week 192	82	117	−8·4 (−14·3 to −2·5)
By presence of subfoveal atrophy			
Week 48	31	42	−8·4 (−15·5 to −1·3)
Week 96	34	47	−4·5 (−11·9 to 3·0)
Week 144	26	57	−3·2 (−12·3 to 5·8)
Week 192	29	67	−6·1 (−15·1 to 3·0)
By presence of ellipsoid zone disruption			
Week 48	30	228	−0·61 (−6·0 to 4·7)
Week 96	37	195	−1·5 (−6·7 to 3·8)
Week 144	28	200	−3·5 (−10·8 to 3·8)
Week 192	29	191	−14·7 (−22·7 to −6·8)
By presence of active exudative AMD			
Week 48	54	172	3·1 (−1·4 to 7·7)
Week 96	71	136	1·5 (−2·8 to 5·8)
Week 144	74	130	0·64 (−4·7 to 6·0)
Week 192	93	101	−1·4 (−7·3 to 4·5)
By total lesion area (> vs ≤ median)			
Week 48	112	114	−7·8 (−11·5 to −4·0)
Week 96	100	107	−8·8 (−12·7 to −4·9)
Week 144	103	101	−9·8 (−14·7 to −4·8)
Week 192	101	93	−10·5 (−16·3 to −4·8)
By total active lesion area (> vs ≤ median)			
Week 48	85	88	−4·5 (−8·7 to −0·33)
Week 96	65	71	−4·2 (−8·7 to 0·30)
Week 144	61	69	−8·7 (−15·1 to −2·3)
Week 192	52	49	−11·4 (−19·9 to −2·9)
By presence of pathology details			
Week 48	167	92	−3·2 (−6·9 to 0·52)
Week 96	153	81	−2·4 (−6·5 to 1·6)
Week 144	122	106	−1·1 (−6·0 to 3·8)
Week 192	136	86	−6·7 (−12·4 to −1·0)
By presence of ERM (pathology details)			
Week 48	188	86	−3·7 (−7·4 to 0·06)
Week 96	198	76	−1·2 (−5·3 to 2·9)
Week 144	176	98	0·00 (−4·9 to 4·9)
Week 192	200	74	−4·7 (−10·6 to 1·1)

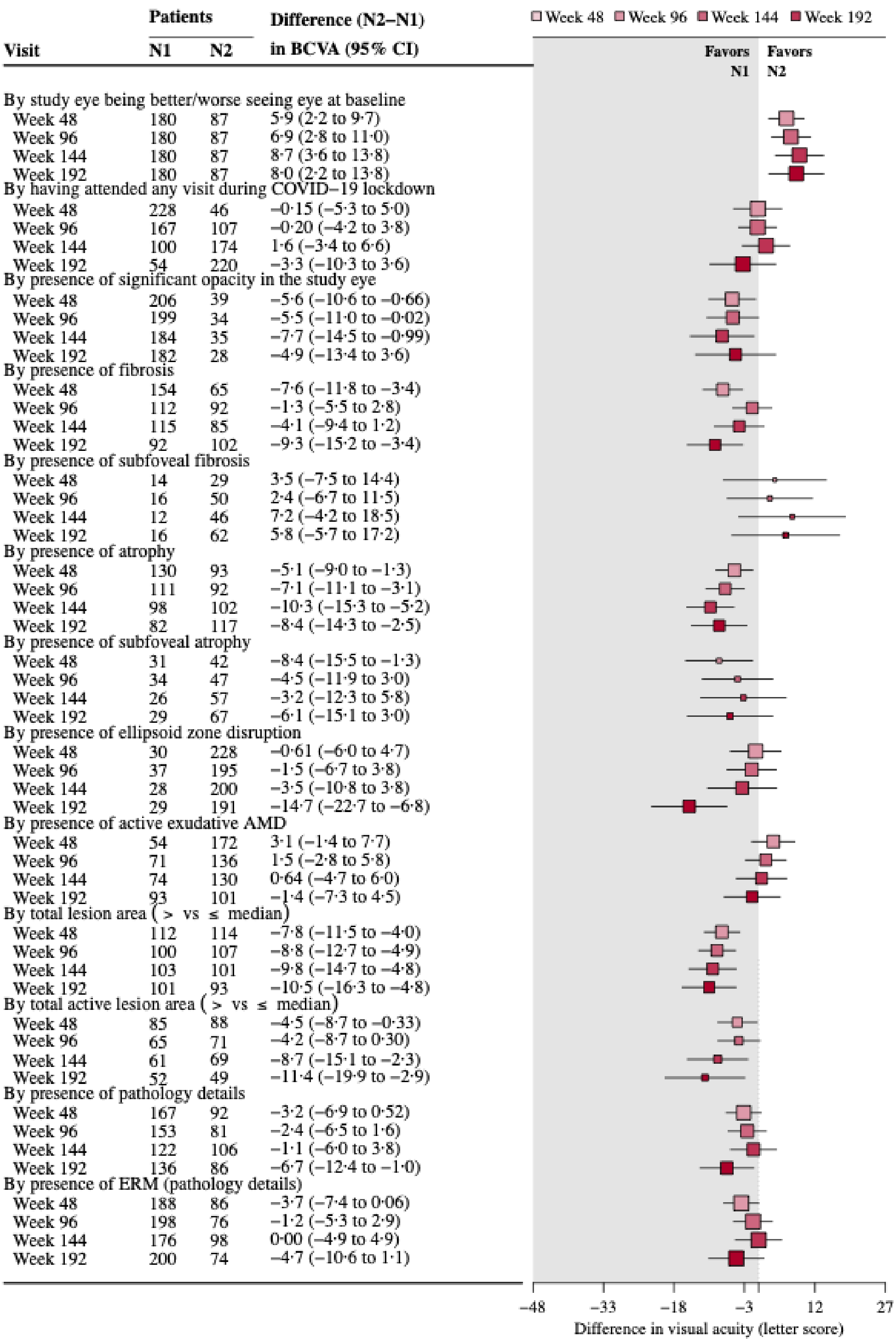

Figure S11: Difference in ETDRS best-corrected visual acuity in SRT group eyes disaggregated by explanatory cause, at each timepoint. For study eye being better/worse seeing eye at baseline, N2 = better seeing eye, N1 = worse seeing eye. For total lesion area and total active lesion area, N2 = greater than median, N1 = less than or equal to median. For all others, N2 = yes, N1 = no. Abbreviations: BCVA, best-corrected visual acuity; ERM, epiretinal membrane; ETDRS, early treatment of diabetic retinopathy study.

41. **Figure S12: Differences in ETDRS best-corrected visual acuity in sham SRT group study eyes disaggregated by explanatory causes for vision loss, at each timepoint.**

Visit	Patients N1	Patients N2	Difference (N2–N1) in BCVA (95% CI)
By study eye being better/worse seeing eye at baseline			
Week 48	80	53	5·5 (0·19 to 10·8)
Week 96	80	53	5·1 (−0·62 to 10·8)
Week 144	80	53	7·0 (0·10 to 13·8)
Week 192	80	53	9·7 (2·2 to 17·2)
By having attended any visit during COVID−19 lockdown			
Week 48	113	24	−10·2 (−17·1 to −3·2)
Week 96	85	52	−4·3 (−10·2 to 1·5)
Week 144	50	87	2·1 (−5·1 to 9·3)
Week 192	27	110	−2·9 (−12·2 to 6·4)
By presence of significant opacity in the study eye			
Week 48	105	15	−2·5 (−10·7 to 5·8)
Week 96	100	13	−3·4 (−12·7 to 5·9)
Week 144	93	17	1·1 (−8·8 to 11·0)
Week 192	75	19	0·84 (−9·2 to 10·9)
By presence of fibrosis			
Week 48	80	25	−7·5 (−13·7 to −1·2)
Week 96	65	36	−0·68 (−6·5 to 5·1)
Week 144	55	39	−9·6 (−16·8 to −2·5)
Week 192	51	41	−8·4 (−16·2 to −0·66)
By presence of subfoveal fibrosis			
Week 48	2	8	6·2 (−29·8 to 42·3)
Week 96	6	18	−3·7 (−17·6 to 10·2)
Week 144	9	20	0·72 (−15·9 to 17·3)
Week 192	4	27	−5·1 (−31·6 to 21·4)
By presence of atrophy			
Week 48	73	34	−5·3 (−10·9 to 0·37)
Week 96	66	35	−4·1 (−9·9 to 1·7)
Week 144	61	35	−2·9 (−10·2 to 4·4)
Week 192	54	38	−4·2 (−12·2 to 3·9)
By presence of subfoveal atrophy			
Week 48	14	14	−4·5 (−18·0 to 9·0)
Week 96	16	15	1·8 (−9·6 to 13·3)
Week 144	13	18	−6·2 (−18·1 to 5·6)
Week 192	10	21	1·5 (−11·2 to 14·2)
By presence of ellipsoid zone disruption			
Week 48	26	96	−0·87 (−7·2 to 5·4)
Week 96	14	99	0·48 (−7·8 to 8·7)
Week 144	21	85	−7·5 (−15·8 to 0·77)
Week 192	14	88	−7·7 (−18·4 to 3·0)
By presence of active exudative AMD			
Week 48	14	93	8·0 (0·22 to 15·7)
Week 96	21	81	6·0 (−0·70 to 12·6)
Week 144	28	68	9·0 (1·2 to 16·7)
Week 192	35	57	2·4 (−5·7 to 10·5)
By total lesion area (> vs ≤ median)			
Week 48	55	52	0·18 (−5·2 to 5·5)
Week 96	54	48	−1·6 (−7·0 to 3·9)
Week 144	47	49	−9·9 (−16·8 to −3·0)
Week 192	43	49	−5·2 (−13·0 to 2·6)
By total active lesion area (> vs ≤ median)			
Week 48	48	45	0·26 (−5·1 to 5·6)
Week 96	43	40	−1·5 (−6·9 to 4·0)
Week 144	38	30	−7·8 (−15·4 to −0·08)
Week 192	27	30	−4·8 (−13·4 to 3·8)
By presence of pathology details			
Week 48	82	41	−5·6 (−10·9 to −0·36)
Week 96	78	35	−5·9 (−11·7 to −0·08)
Week 144	73	33	−12·5 (−19·3 to −5·6)
Week 192	67	36	−2·3 (−10·0 to 5·5)
By presence of ERM (pathology details)			
Week 48	100	37	−5·4 (−10·9 to 0·20)
Week 96	105	32	−5·2 (−11·6 to 1·1)
Week 144	107	30	−13·1 (−20·5 to −5·7)
Week 192	104	33	−1·9 (−10·2 to 6·4)

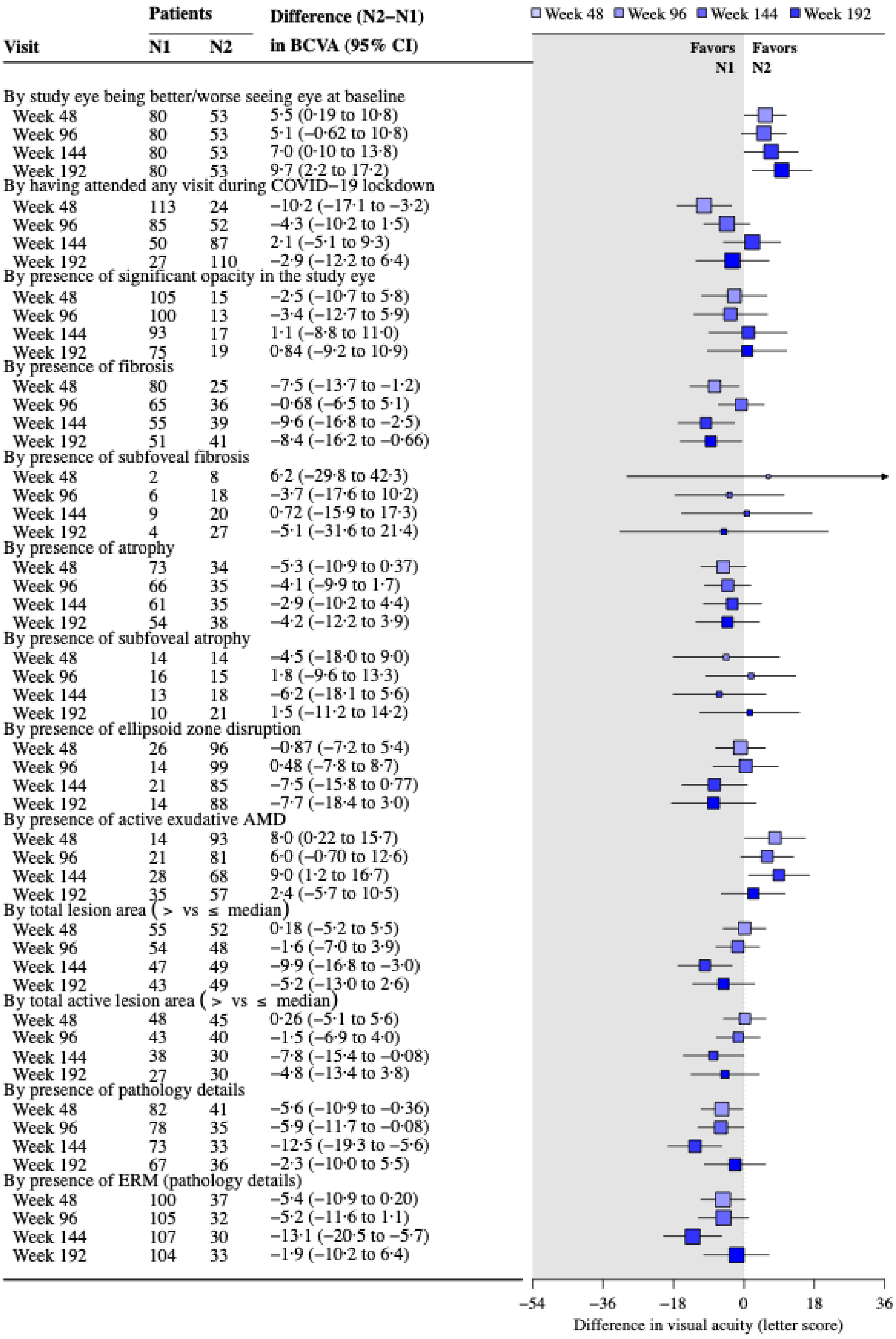

Figure S12: Difference in ETDRS best-corrected visual acuity in sham SRT group eyes disaggregated by explanatory cause, at each timepoint. For study eye being better/worse seeing eye at baseline, N2 = better seeing eye, N1 = worse seeing eye. For total lesion area and total active lesion area, N2 = greater than median, N1 = less than or equal to median. For all others, N2 = yes, N1 = no. Abbreviations: BCVA, best-corrected visual acuity; ERM, epiretinal membrane; ETDRS, early treatment of diabetic retinopathy study.

Safety: Adverse events

42. **Table S23: Adverse events and serious adverse events in the study eye by received treatment over 192 weeks**

	**Adverse events**		**Serious adverse events**	
	**SRT** (N=276)	**Sham SRT** (N=133)	**SRT** (N=276)	**Sham SRT** (N=133)
Total number of events (n)	169	65	110	22
Participants with event/s	108 (39%)	45 (34%)	66 (24%)	18 (14%)
1 event	71 (26%)	32 (24%)	40 (14%)	15 (11%)
2 events	23 (8%)	8 (6%)	18 (7%)	2 (2%)
3 events	8 (3%)	3 (2%)	4 (1%)	1 (<1%)
≥ 4 events	6 (2%)	2 (2%)	4 (1%)	-
Days to first event, median [IQR]	230 [69-478]	209 [85-362]	463 [253-906]	274 [146-414]
Ocular events				
Cataract/cataract surgery	-	-	38 (14%)	14 (11%)
Macular haemorrhage	2 (<1%)	-	4 (1%)	-
Epiretinal membrane	-	-	1 (<1%)	-
Macular hole	-	-	-	-
CSCR	-	-	-	-
Conjunctival/subconjunctival haemorrhage	14 (5%)	6 (5%)	2 (<1%)	-
Endophthalmitis	-	-	1 (<1%)	1 (<1%)
YAG capsulotomy	9 (3%)	-	4 (1%)	-
Ocular Events of Special Interest: Retinal microvascular changes (mandated reporting as an SAE)*	-	-	23 (8%)	-
Telangiectasia	-	-	2 (<1%)	-
Cotton wool spot	-	-	5 (2%)	-
Exudates	-	-	2 (<1%)	-
Retinal haemorrhage	-	-	10 (4%)	-
Retinal microaneurysm	-	-	4 (1%)	-
Retinal neovascularisation	-	-	-	-
Optic disc neovascularisation	-	-	-	-
Capillary dilation	-	-	-	-
Perivascular sheathing	-	-	-	-
Other retinopathy suspected related to radiotherapy	-	-	12 (4%)	-
AE body system code				
Eye disorders	102 (37%)	43 (32%)	59 (21%)	12 (9%)
Surgical and medical procedures	2 (<1%)	-	-	1 (<1%)
Infections and infestations	3 (1%)	3 (2%)	-	-
Injury, poisoning and procedural complications	1 (<1%)	-	-	-
Investigations	1 (<1%)	-	-	-
Neoplasms benign, malignant and unspecified	1 (<1%)	-	-	-
Nervous system disorders	1 (<1%)	1 (<1%)	-	-
Skin and subcutaneous tissue disorders	6 (2%)	-	16 (6%)	6 (5%)

*Table S23: Data are n (%), median [interquartile range]. Multiple events in the same category were counted only once. *To ensure prompt and vigilant reporting of any radiation-related retinal damage, the protocol instructed site investigators to report any retinal microvascular change as a serious adverse event (SAE), even if it would not otherwise meet the definition of an SAE. Abbreviations: SAE, serious adverse event; SD, standard deviation; SRT, stereotactic radiotherapy; YAG, Yttrium-Aluminium-Garnet*

43. **Table S24: All adverse events and serious adverse events by received treatment over 192 weeks**

	**AEs**		**SAEs**		**Combined AEs and SAEs**	
	**SRT** (N=276)	**Sham SRT** (N=133)	**SRT** (N=276)	**Sham SRT** (N=133)	**SRT** (N=276)	**Sham SRT** (N=133)
Total number of events (n)	988	471	304	113	1293	585
Participants with event/s	223 (81%)	104 (78%)	148 (54%)	63 (47%)	244 (88%)	112 (84%)
1 event	47 (17%)	26 (20%)	69 (25%)	35 (26%)	37 (13%)	19 (14%)
2 events	40 (14%)	17 (13%)	39 (14%)	17 (13%)	43 (16%)	23 (17%)
3 events	28 (10%)	15 (11%)	25 (9%)	3 (2%)	32 (12%)	14 (11%)
≥ 4 events	108 (39%)	46 (35%)	15 (5%)	8 (6%)	132 (48%)	56 (42%)
Days to first event, median [IQR]	134 [47-297]	162 [76-335]	440 [198-684]	397 [174-650]	136 [46-302]	146 [56-296]
Deaths	-	-	17 (6%)	5 (4%)	17 (6%)	5 (4%)
Arterial thromboembolic event (AE of special interest)	4 (1%)	2 (2%)	7 (3%)	4 (3%)	11 (4%)	6 (5%)
Cerebrovascular Event	1 (2%)	1 (3%)	1 (<1%)	2 (3%)	2 (5%)	3 (12%)
Myocardial Infarction	1 (2%)	-	2 (2%)	1 (1%)	3 (8%)	1 (4%)
Acute Myocardial Infarction	1 (2%)	-	2 (2%)	-	3 (8%)	-
AE body system code						
Blood and lymphatic system disorders	12 (4%)	2 (2%)	-	2 (2%)	12 (4%)	4 (3%)
Cardiac disorders	30 (11%)	14 (11%)	13 (5%)	6 (5%)	38 (14%)	17 (13%)
Congenital, familial and genetic disorders	-	-	-	-	-	-
Ear and labyrinth disorders	18 (7%)	8 (6%)	2 (<1%)	-	20 (7%)	8 (6%)
Endocrine disorders	5 (2%)	2 (2%)	4 (1%)	-	9 (3%)	2 (2%)
Eye disorders	121 (44%)	53 (40%)	75 (27%)	22 (17%)	152 (55%)	66 (50%)
Gastrointestinal disorders	50 (18%)	21 (16%)	11 (4%)	-	57 (21%)	21 (16%)
General disorders and administration site conditions	25 (9%)	16 (12%)	-	-	25 (9%)	16 (12%)
Hepatobiliary disorders	4 (1%)	2 (2%)	6 (2%)	2 (2%)	9 (3%)	3 (2%)
Immune system disorders	5 (2%)	1 (<1%)	-	-	5 (2%)	1 (<1%)
Infections and infestations	98 (36%)	49 (37%)	9 (3%)	6 (5%)	101 (37%)	51 (38%)
Injury, poisoning and procedural complications	35 (13%)	16 (12%)	7 (3%)	4 (3%)	39 (14%)	19 (14%)
Investigations	14 (5%)	5 (4%)	-	-	14 (5%)	5 (4%)
Metabolism and nutrition disorders	9 (3%)	4 (3%)	1 (<1%)	1 (<1%)	10 (4%)	4 (3%)
Musculoskeletal and connective tissue disorders	69 (25%)	34 (26%)	6 (2%)	5 (4%)	74 (27%)	36 (27%)
Neoplasms benign, malignant and unspecified	12 (4%)	7 (5%)	12 (4%)	8 (6%)	24 (9%)	11 (8%)
Nervous system disorders	21 (8%)	11 (8%)	4 (1%)	2 (2%)	24 (9%)	13 (10%)
Psychiatric disorders	3 (1%)	4 (3%)	1 (<1%)	1 (<1%)	3 (1%)	5 (4%)
Retinal and urinary disorders	21 (8%)	8 (6%)	6 (2%)	5 (4%)	24 (9%)	11 (8%)
Reproductive system and breast disorders	3 (1%)	4 (3%)	1 (<1%)	1 (<1%)	4 (1%)	5 (4%)
Respiratory, thoracic and mediastinal disorders	39 (14%)	23 (17%)	15 (5%)	6 (5%)	49 (18%)	26 (20%)
Skin and subcutaneous tissue disorders	37 (13%)	17 (13%)	-	-	37 (13%)	17 (13%)
Social circumstances	3 (1%)	2 (2%)	-	-	3 (1%)	2 (2%)

Surgical and medical procedures	35 (13%)	15 (11%)	45 (16%)	17 (13%)	72 (26%)	27 (20%)
Vascular disorders	17 (6%)	3 (2%)	3 (1%)	3 (2%)	20 (7%)	6 (5%)

*Table S24: Data are n (%) or median [interquartile range]. *Multiple events in the same category were counted only once. Abbreviations: AE, adverse event; IQR, interquartile range; SRT, stereotactic radiotherapy.*

44. **Table S25: Intensity and relatedness of adverse events in the study eye over 192 weeks**

	AEs related to study eye		SAEs related to study eye	
	**SRT** (N=169)	**Sham SRT** (N=65)	**SRT** (N=110)	**Sham SRT** (N=22)
Intensity				
Mild	125 (74%)	43 (66%)	65 (59%)	7 (32%)
Moderate	34 (20%)	18 (28%)	36 (33%)	9 (41%)
Severe	10 (6%)	4 (6%)	9 (8%)	6 (27%)
Relatedness to any intervention				
Definite	18 (11%)	7 (11%)	-	-
Probable	18 (11%)	11 (17%)	13 (12%)	1 (5%)
Possible	27 (16%)	7 (11%)	48 (44%)	4 (18%)
Remote	14 (8%)	8 (12%)	29 (26%)	9 (41%)
None	92 (54%)	32 (49%)	20 (18%)	8 (36%)
Relatedness to SRT				
Definite	-	-	-	-
Probable	2 (1%)	1 (2%)	10 (9%)	-
Possible	16 (9%)	2 (3%)	39 (35%)	2 (9%)
Remote	13 (8%)	7 (11%)	36 (33%)	10 (45%)
None	138 (82%)	55 (85%)	25 (23%)	10 (45%)
Relatedness to anti-VEGF treatment				
Definite	-	-	-	-
Probable	2 (1%)	1 (2%)	1 (<1%)	-
Possible	14 (8%)	2 (3%)	10 (9%)	2 (9%)
Remote	12 (7%)	7 (11%)	13 (12%)	5 (23%)
None	141 (83%)	55 (85%)	86 (78%)	15 (68%)
Relatedness to intravitreal injection procedure				
Definite	18 (11%)	7 (11%)	-	-
Probable	16 (9%)	11 (17%)	3 (3%)	1 (5%)
Possible	20 (12%)	6 (9%)	10 (9%)	3 (14%)
Remote	11 (7%)	6 (9%)	11 (10%)	2 (9%)
None	104 (62%)	35 (54%)	86 (78%)	16 (73%)
Outcomes				
Ongoing	58 (34%)	21 (33%)	44 (40%)	2 (9%)
Resolved with sequelae	12 (7%)	6 (10%)	17 (15%)	11 (50%)
Resolved without sequelae	97 (57%)	36 (57%)	49 (45%)	9 (41%)

Table S25: Data are count (%, to number of events in corresponding group). Populations defined according to the received treatment. Abbreviations: anti-VEGF, anti-Vascular Endothelial Growth Factor; SRT, stereotactic radiotherapy.

45. **Table S26: Intensity and relatedness of adverse events over 192 weeks**

	Adverse Events		Serious Adverse Events	
	**SRT** (N=988)	**Sham SRT** (N=471)	**SRT** (N=304)	**Sham SRT** (N=113)
Intensity				
Mild	593 (61%)	274 (59%)	87 (29%)	16 (14%)
Moderate	324 (33%)	171 (37%)	101 (33%)	45 (40%)
Severe	60 (6%)	23 (5%)	114 (38%)	51 (46%)
Relatedness to any intervention				
Definite	20 (2%)	9 (2%)	-	-
Probable	21 (2%)	12 (3%)	14 (5%)	2 (2%)
Possible	35 (4%)	11 (2%)	53 (17%)	4 (4%)
Remote	33 (3%)	15 (3%)	46 (15%)	19 (17%)
None	879 (89%)	421 (90%)	191 (63%)	87 (78%)
Relatedness to SRT				
Definite	1 (<1%)	1 (<1%)	-	-
Probable	2 (<1%)	2 (<1%)	10 (3%)	-
Possible	16 (2%)	2 (<1%)	39 (13%)	2 (2%)
Remote	24 (2%)	10 (2%)	45 (15%)	14 (12%)
None	945 (96%)	453 (97%)	210 (69%)	96 (86%)
Relatedness to anti-VEGF treatment				
Definite	-	-	-	-
Probable	3 (<1%)	1 (<1%)	2 (<1%)	1 (<1%)
Possible	19 (2%)	3 (<1%)	12 (4%)	2 (2%)
Remote	20 (2%)	11 (2%)	23 (8%)	11 (10%)
None	946 (96%)	453 (97%)	267 (88%)	98 (88%)
Relatedness to intravitreal injection procedure				
Definite	19 (2%)	8 (2%)	-	-
Probable	18 (2%)	11 (2%)	3 (<1%)	1 (<1%)
Possible	24 (2%)	10 (2%)	13 (4%)	3 (3%)
Remote	18 (2%)	6 (1%)	15 (5%)	3 (3%)
None	909 (92%)	433 (93%)	273 (90%)	105 (94%)
Outcomes				
Ongoing	301 (31%)	147 (32%)	92 (30%)	22 (20%)
Resolved with sequelae	96 (10%)	41 (9%)	61 (20%)	31 (28%)
Resolved without sequelae	578 (59%)	269 (58%)	132 (43%)	51 (46%)
AE related to study eye	169 (17%)	65 (14%)	110 (36%)	22 (19%)

Table S26: Data are count (%, to number of events in corresponding group). Populations defined according to the received treatment. Abbreviations: AE, adverse event; anti-VEGF, anti-Vascular Endothelial Growth Factor; SRT, stereotactic radiotherapy.

Safety: Microvascular abnormalities and impact on acuity

46. **Table S27: Mean change in best-corrected visual acuity from baseline by reading centre-determined microvascular abnormality status and fovea involvement**

	Count (N)		Difference in ETDRS BCVA from baseline	
	SRT	Sham SRT	SRT	Sham SRT
**MVA positive**				
Week 48	45	10	-0·3 (9·0)	0·3 (9·2)
Week 96	87	15	-0·1 (9·1)	5·1 (13)
Week 144	121	18	-10 (16)	1·9 (12)
Week 192	126	16	-17 (19)	-4·1 (11)
**MVA negative**				
Week 48	200	104	0·2 (8·8)	0·8 (8·0)
Week 96	139	94	-4·1 (12)	-1·5 (8·8)
Week 144	99	88	-8·8 (16)	-3·7 (13)
Week 192	92	86	-12 (18)	-6·9 (16)
**Fovea-involving MVA**				
Week 48	9	1	-	-
Week 96	27	3	-3·2 (10)	7·3 (8·0)
Week 144	45	3	-14 (18)	1·7 (7·6)
Week 192	52	5	-20 (21)	0·0 (8·2)
**Non-fovea-involving MVA**				
Week 48	35	9	0·0 (8·1)	1·3 (9·1)
Week 96	58	12	0·9 (8·9)	4·5 (14)
Week 144	73	14	-8·3 (14)	1·1 (13)
Week 192	71	11	-15 (18)	-6·0 (11)

*Table S27: Data are mean (standard deviation) and detail the findings of the independent reading centre. *Discrepancy between total MVA positive eyes and total of Fovea-involving MVA and Non-fovea-involving MVA eyes is due to masking from blood or cotton wool spots such that grading of fovea involvement was not possible. Abbreviations: BCVA, best-corrected visual acuity; ETDRS, Early Treatment Diabetic Retinopathy Study; MVA, microvascular abnormality; SRT, stereotactic radiotherapy.*

47. **Table S28: Baseline characteristics of participants with and without complete primary outcome data at follow-up**

	Week 144 primary outcome		Week 192 primary outcome	
	**Complete (N=334)**	**Missing (N=77)**	**Complete (N=319)**	**Missing (N=92)**
**Demographic characteristics**				
Age (years)	77·0 (6·8)	80·4 (7·7)	76·8 (6·9)	80·4 (7·2)
Female	198 (59%)	42 (55%)	184 (58%)	56 (61%)
Ethnicity				
White	324 (97%)	73 (95%)	309 (97%)	88 (96%)
Black or Black British	1 (<1%)	-	1 (<1%)	-
Asian or Asian British	6 (2%)	4 (5%)	6 (2%)	4 (4%)
Other	3 (<1%)	-	3 (<1%)	-
Smoking status				
Current	34 (10%)	8 (10%)	34 (11%)	8 (9%)
Ex	145 (43%)	30 (39%)	137 (43%)	38 (41%)
Non	155 (46%)	39 (51%)	148 (46%)	46 (50%)
**Ophthalmic characteristics**				
nAMD duration (months)*	21 [11-42]	23 [12-46]	21 [11-42]	23 [12-48]
Number of previous anti-VEGF injections	7 [5-10]	7 [5-10]	7 [5-10]	7 [5-10]
ETDRS visual acuity (letters score)*				
Lens status				
Aphakic	-	3 (4%)	-	3 (3%)
Pseudophakic	103 (31%)	34 (44%)	94 (29%)	43 (47%)
Phakic	231 (69%)	40 (52%)	225 (71%)	46 (50%)
Central subfield thickness (µm)	345·9 (118·3)	353·6 (126·6)	343·8 (112·6)	359·9 (142·1)
Total lesion size (mm^2)*	7 [4-11]	6 [4-10]	7 [4-11]	7 [4-12]
Total active lesion size (mm^2)*	7 [4-10]	6 [4-9]	6 [4-10]	6 [4-11]
Total macular volume (mm^3)	8·8 (1·1)	8·9 (1·5)	8·8 (1·1)	9·0 (1·5)
**Patient-reported quality of life**				
NEI VFQ-25 composite score*	87 [75-94]	88 [73-93]	87 [77-94]	86 [69-92]
EQ-5D-5L (VAS)*	90 [75-95]	85 [75-95]	90 [75-95]	85 [75-95]
EQ-5D-5L dimension				
Mobility				
No problems	225 (69%)	39 (51%)	220 (71%)	44 (48%)
Slight problems	47 (14%)	20 (26%)	46 (15%)	21 (23%)
Moderate problems	45 (14%)	11 (14%)	35 (11%)	21 (23%)
Severe problems	8 (2%)	6 (8%)	9 (3%)	5 (5%)
Extreme problems	2 (<1%)	-	2 (<1%)	-
Self-care				
No problems	303 (93%)	70 (92%)	290 (93%)	83 (91%)
Slight problems	15 (5%)	5 (7%)	13 (4%)	7 (8%)
Moderate problems	7 (2%)	-	7 (2%)	-
Severe problems	1 (<1%)	1 (1%)	1 (<1%)	1 (1%)
Extreme problems	1 (<1%)	-	1 (<1%)	-
Usual activities				
No problems	236 (72%)	49 (64%)	231 (74%)	54 (59%)
Slight problems	57 (17%)	14 (18%)	53 (17%)	18 (20%)
Moderate problems	29 (9%)	10 (13%)	23 (7%)	16 (18%)
Severe problems	3 (<1%)	3 (4%)	3 (<1%)	3 (3%)
Extreme problems	2 (<1%)	-	2 (<1%)	-
Pain/discomfort				
No problems	184 (56%)	36 (47%)	178 (57%)	42 (46%)
Slight problems	82 (25%)	23 (30%)	78 (25%)	27 (30%)
Moderate problems	46 (14%)	13 (17%)	42 (13%)	17 (19%)
Severe problems	14 (4%)	3 (4%)	13 (4%)	4 (4%)
Extreme problems	1 (<1%)	1 (1%)	1 (<1%)	1 (1%)
Anxiety/depression				
No problems	263 (80%)	67 (88%)	253 (81%)	77 (85%)
Slight problems	44 (13%)	7 (9%)	41 (13%)	10 (11%)
Moderate problems	19 (6%)	2 (3%)	17 (5%)	4 (4%)
Severe problems	1 (<1%)	-	1 (<1%)	-
Extreme problems	-	-	-	-

*Table S28: Data are n (%) or mean (SD) unless marked with an asterisk. All ophthalmic history variables relate to the study eye. Anti-VEGF=anti-vascular endothelial growth factor. EQ-5D-5L (VAS)=EuroQol-5D questionnaire with visual analogue scale. ETDRS=Early Treatment Diabetic Retinopathy Study. IPCV=idiopathic polypoidal choroidal vasculopathy. nAMD=neovascular age-related macular degeneration. NEI-VFQ-25=National Eye Institute (USA) 25 item visual function questionnaire. *Median (IQR).*

48. Table S29: Baseline characteristics of participants disaggregated by sex

	Male		Female	
	**SRT (N=116)**	**Sham SRT (N=55)**	**SRT (N=158)**	**Sham SRT (N=82)**
**Demographic characteristics**				
Age (years)	77·2 (7·1)	76·6 (7·0)	78·0 (6·9)	78·2 (7·6)
Ethnicity				
White	112 (97%)	50 (91%)	156 (99%)	79 (96%)
Black or Black British	-	-	1 (<1%)	-
Asian or Asian British	3 (3%)	4 (7%)	-	3 (4%)
Other	1 (<1%)	1 (2%)	1 (<1%)	-
Smoking status				
Current	11 (9%)	8 (15%)	16 (10%)	7 (9%)
Ex	66 (57%)	26 (47%)	48 (30%)	35 (43%)
Non	39 (34%)	21 (38%)	94 (59%)	40 (49%)
**Ophthalmic characteristics**				
AMD duration (months)	22 [11-46]	22 [17-48]	21 [11-43]	23 [12-37]
Number of previous anti-VEGF injections	6 [4-9]	8 [5-12]	7 [5-10]	6 [4-9]
ETDRS visual acuity (letters score)				
Lens status				
Aphakic	-	1 (2%)	1 (<1%)	1 (1%)
Pseudophakic	33 (28%)	17 (31%)	59 (37%)	28 (34%)
Phakic	83 (72%)	37 (67%)	98 (62%)	53 (65%)
Central subfield thickness (µm)	356·8 (111·5)	350·5 (151·3)	343·8 (116·9)	338·6 (113·9)
Total lesion size (mm^2)	7 [4-11]	6 [4-9]	7 [4-11]	8 [4-12]
Total active lesion size (mm^2)	6 [3-10]	6 [4-9]	7 [4-10]	8 [4-11]
Total macular volume (mm^3)	8·7 (0·9)	9·0 (1·5)	8·8 (1·3)	8·8 (1·1)
**Patient-reported quality of life**				
NEI VFQ-25 composite score	89 [79-95]	92 [85-95]	86 [74-94]	82 [58-91]
EQ-5D-5L (VAS)	90 [80-95]	90 [80-95]	85 [75-95]	80 [75-90]
EQ-5D-5L dimension				
Mobility				
No problems	88 (77%)	44 (80%)	93 (61%)	39 (48%)
Slight problems	13 (11%)	8 (15%)	24 (16%)	22 (27%)
Moderate problems	8 (7%)	3 (5%)	29 (19%)	16 (20%)
Severe problems	5 (4%)	-	5 (3%)	4 (5%)
Extreme problems	1 (<1%)	-	1 (<1%)	-
Self-care				
No problems	110 (96%)	54 (98%)	137 (90%)	72 (89%)
Slight problems	4 (3%)	1 (2%)	8 (5%)	7 (9%)
Moderate problems	1 (<1%)	-	4 (3%)	2 (2%)
Severe problems	-	-	2 (1%)	-
Extreme problems	-	-	1 (<1%)	-
Usual activities				
No problems	92 (80%)	46 (84%)	99 (65%)	48 (59%)
Slight problems	15 (13%)	5 (9%)	33 (22%)	18 (22%)
Moderate problems	6 (5%)	3 (5%)	16 (11%)	14 (17%)
Severe problems	2 (2%)	1 (2%)	3 (2%)	-
Extreme problems	-	-	1 (<1%)	1 (1%)
Pain/discomfort				

No problems	70 (61%)	40 (73%)	74 (49%)	36 (44%)
Slight problems	31 (27%)	9 (16%)	43 (28%)	22 (27%)
Moderate problems	10 (9%)	4 (7%)	28 (18%)	17 (21%)
Severe problems	3 (3%)	2 (4%)	6 (4%)	6 (7%)
Extreme problems	1 (<1%)	-	1 (<1%)	-
Anxiety/depression				
No problems	101 (88%)	45 (82%)	120 (79%)	64 (79%)
Slight problems	8 (7%)	9 (16%)	22 (14%)	12 (15%)
Moderate problems	6 (5%)	1 (2%)	9 (6%)	5 (6%)
Severe problems	-	-	1 (<1%)	-
Extreme problems	-	-	-	-

Table S29: Data are n (%), mean (standard deviation), or median [interquartile range] marked with an asterisk. All ophthalmic history variables relate to the study eye. Missingness is provided in Table S5-6, pp34-35. Abbreviations: anti-VEGF, anti- vascular endothelial growth factor; EQ-5D-5L (VAS), EuroQol-5D questionnaire with visual analogue scale; ETDRS, Early Treatment Diabetic Retinopathy Study; IQR, interquartile range; nAMD, neovascular age-related macular degeneration; NEI-VFQ-25, National Eye Institute (USA) 25-item visual function questionnaire; RAP, retinal angiomatous proliferation; IPCV, idiopathic polypoidal choroidal vasculopathy; SD, standard deviation; SRT, stereotactic radiotherapy; mm2, millimeter squared; mm3, millimeter cubed; µm, micrometer.*

49. **Study investigators**
- Salwa Abugreen: Royal Blackburn Hospital, Blackburn
- Rashi Arora: Salisbury District Hospital, Odstock
- Mandeep Bindra: Stoke Mandeville Hospital, Aylesbury
- Ben Burton: James Paget University Hospital, Great Yarmouth
- Indra Dias: Calderdale Royal Hospital, Halifax
- Christiana Dinah: London North West University Healthcare NHS Trust
- Ravikiran Gandhewar: Royal Derby Hospital, Derby
- Marianne Shiew and Athanasios Georgas: Hinchingbrooke Hospital, Hinchingbrooke
- Sheena George: Hillingdon Hospital, London
- Srinivas Goverdhan: Dorset County Hospital, Dorchester
- Eleni Vrizidou, Ramiro Salom and Ansari Gulrez: Queens Hospital, Romford
- Richard Haynes: Bristol Eye Hospital, Bristol
- Edward Hughes: Sussex Eye Hospital, Brighton
- Robert Petrarca and Shahrnaz Izadi: Epsom & St Helier's Hospital, Carshalton
- Timothy Jackson (Chief Invstigator): King's College Hospital, London
- Afsar Jafree: William Harvey Hospital, Ashford
- Sobha Joseph: University Hospitals Birmingham NHS Foundation Trust, Birmingham
- Tarek Kashab: Mid Cheshire Hospital, Crewe
- Luke Membrey: Maidstone Hospital, Maidstone
- Geeta Menon: Frimley Park Hospital, Frimley
- Aseema Misra: Norfolk & Norwich University Hospital, Norwich
- Niro Narendran: New Cross Hospital, Wolverhampton
- Douglas Newman: Cambridge University Hospitals, Cambridge
- Jignesh Patel: Essex County Hospital, Colchester
- Sudeshna Patra: Barts Health NHS Trust, London
- Priya Prakash and Silvana Madi: Princess Alexandra Hospital, Harlow
- Paritosh Shah: Yeovil Hospital, Yeovil
- Paul Tesha: United Lincolnshire Hospital Trust, Lincoln

50. **National Treatment Centres**

Lead clinician
- Chris Brand: Royal Hallamshire Hospital, Sheffield
- Timothy Jackson: King's College Hospital, London
- Ramesh Sivaraj: University Hospitals Birmingham NHS Foundation Trust, Birmingham
- Aseema Misra: Norfolk & Norwich University Hospital, Norwich
- Ramandeep Chhabra: Manchester Royal Eye Hospital, Manchester

Lead medical physicist or Radiation protection officer
- Giles D. Morrison: Sheffield Teaching Hospitals NHS Foundation Trust, Sheffield
- Cornelius Lewis and Patricia Clinch: King's College Hospital, London
- Jane Waller: Solihull Hospital, Birmingham
- Nattapon Boonarpha: Manchester Royal Eye Hospital, Manchester
- Amanda Webster: Norfolk & Norwich University Hospital, Norwich

51. **Trial Steering Committee members**
- Richard Wormald, Cochrane Eyes and Vision Group, International Centre for Eye Health, London School of Hygiene and Tropical Medicine (independent voting clinical chair)
- Winfried Amoaku, Associate Professor and Reader in Ophthalmology and Visual Sciences, University of Nottingham (independent voting clinician)
- Clare Bailey, Consultant Ophthalmic Surgeon, Bristol Eye Hospital (independent voting clinician)
- Professor Timothy Jackson, Professor of Retinal Research and Consultant Ophthalmic Surgeon, King's College London and King's College Hospital (voting clinical chief investigator)

- Luke Membrey, Consultant Ophthalmic Surgeon, Maidstone Hospital (non-voting principal investigators' representative)
- Professor Barnaby Reeves, Professor of Health Services Research, University of Bristol (non-voting trialist)
- Professor Mandeep Sagoo, Professor of Ophthalmology and Ocular Oncology and Consultant Ocular Oncologist, University College London and Moorfields and St Bartholomew's Hospital (independent voting clinician)
- Professor Yanzhong Wang, Professor of Medical Statistics, King's College London (voting trial statistician)
- Professor Robert West, Professor of Biostatistics, Leeds Institute of Health Sciences (independent voting statistician)
- Cathy Yelf, Chief Executive Officer, Macular Society (non-voting lay representative)

52. **Data Monitoring and Ethics Committee members**

- Professor Craig Ramsay, Professor of Health Care Evaluations, Health Services Research Unit, University of Aberdeen
- Professor Paulo Stanga, Professor of Ophthalmology and Retinal Regeneration and Consultant Ophthalmologist and Vitreoretinal Surgeon, University of Manchester and Manchester Royal Eye Hospital
- Professor Heinrich Heimann, Professor of Ophthalmology and Consultant Ocular Oncologist, University of Liverpool and Royal Liverpool University Hospital

53. **Study Reading Centre members**

- Belfast Ophthalmic Reading Centre: Tunde Peto, Usha Chakravarthy, Alan Sproule, Alyson Muldrew, Barbra Hamill, Catherine Jamison, Graham Young, Malcolm Brown, Michael Quinn, Peter Blows
- Liverpool Ophthalmic Reading Centre: Savita Madhusudhan, Pauline Lenfestey, Alia Ali, David Parry, Handan Akil, Sophie Leach
- Moorfields Ophthalmic Reading Centre: Daniela Florea, Irene Leung
- Central Administrative Research Facility (CARF): Vittorio Silvestri, Michelle McGaughey, Clare Newell, Karleigh Kelso, Sara Shields
